# Identification of recurrences in women diagnosed with early invasive breast cancer using routinely collected data in England

**DOI:** 10.1038/s44276-025-00154-1

**Published:** 2025-05-28

**Authors:** Jake Probert, David Dodwell, John Broggio, Robert Coleman, Helen Marshall, Sarah C. Darby, Gurdeep S. Mannu

**Affiliations:** 1 Nuffield Department of Population Health, University of Oxford, Oxford, UK; 2 National Cancer Registration and Analysis Service, National Disease Registration Service, Birmingham, UK; 3 Department of Oncology and Metabolism, University of Sheffield, Sheffield, UK; 4 Leeds Cancer Research Clinical Trial Unit, University of Leeds, Leeds, UK; 5 Nuffield Department of Surgical Sciences, University of Oxford, Oxford, UK

## Abstract

**Background:**

Breast cancer is the commonest cancer in the UK, with around 55,000 women diagnosed annually. Information is routinely available on breast cancer mortality but not on recurrence.

**Methods:**

We used a database compiled by the West Midlands Cancer Intelligence Unit during 1997–2011 to develop and train a deterministic algorithm to identify recurrences in routinely collected data (RCD) available within NHS England. We trained the algorithm further using 150 women with stage II-III breast cancer who were recruited into the AZURE trial during 2003–2006 and invited to approximately 24 clinic follow-up visits over ten years. We then evaluated its performance using data for the remaining 1930 women in England in the AZURE trial.

**Results:**

The sensitivity of the RCD to detect distant recurrences recorded in the AZURE trial during the ten years following randomisation was 95.6% and its sensitivity to detect any recurrence was 96.6%. The corresponding specificities were 91.9% for distant recurrence and 77.7% for any recurrence.

**Conclusions:**

These findings demonstrate the potential of routinely collected data to identify breast cancer recurrences in England. The algorithm may have a role in several settings and make long-term follow-up in randomised trials of breast cancer treatments more cost-effective.

## Introduction

Invasive breast cancer (IBC) is the commonest cancer in the UK, with around 55,000 women diagnosed annually. Incidence rates have been increasing since the mid-1970 s and it now accounts for thirty percent of cancers diagnosed in women [1, 2]. Although population-based information is available on incidence and mortality [3], information on recurrence of IBC is not collected reliably in England and it is available only via individual follow-up, for example in clinical trials [4]. Reliable recurrence information in routine care would be helpful for many purposes, including healthcare policy-making (e.g. guiding decisions on optimal follow-up), guiding clinical management (e.g. in clinical decision aids), making randomised trials more cost-effective (e.g. enabling low-cost long-term follow-up) and for descriptive and analytical epidemiology.

Recurrence of IBC can be divided into three types: locoregional (i.e. recurrence in the ipsilateral breast and/or regional lymph nodes only), distant (i.e. diagnosis of breast cancer metastasis elsewhere in the body that was not detected at the time of diagnosis) or contralateral (i.e. a new diagnosis of IBC in the opposite breast to the original cancer). Several registries have developed methods for identifying distant metastases via their healthcare systems [5, 6] and, while the SEER database in the USA does not include information on locoregional or distant recurrences, it does collect information on contralateral breast recurrences [7]. The reason that information on breast cancer recurrence is not routinely available in England is that cancer recurrence has not traditionally been included in the standard National Health Service (NHS) information flow. Some attempts to rectify this have been made [8], but the quality of the resulting data has, to date, proved inadequate, with many hospital trusts reporting only a small number of recurrences compared to the number of primary cancers in their area [9]. This is especially important for IBC where, although survival is good, recurrences may still occur many years after the diagnosis of the primary cancer.

If the routinely collected data (RCD) held by NHS England could identify IBC recurrences reliably and accurately, they could be used for the above purposes. The aims of this study were therefore: to collate all RCD available within NHS England that are potentially informative regarding a recurrence of IBC; to develop and train a deterministic algorithm based just on RCD to identify recurrences in women with IBC; and to examine the validity of the RCD-based algorithm by comparison with a randomised trial in which patients had been followed via individual clinic visits.

## Methods

### Training data and initial algorithm development

The former West Midlands Cancer Intelligence Unit (WMCIU) was the lead cancer registry for breast cancer in England for many years. In this capacity, it developed a system for collecting detailed recurrence and other clinical information through a meticulous process of contacting individual hospitals for women living within the West Midlands Regional Health Authority area and registered with IBC [10, 11]. The database in which the information from this process was stored was initiated in 1994 and was judged by staff of the former WMCIU to be complete from the beginning of 1997 until the end of 2011, after which time data collection by individual regional registries began to be replaced by nationwide systems, and the WMCIU database was no longer regularly updated.

A total of 52,446 women living in the West Midlands Regional Health Authority area were registered with IBC (ICD9 174; ICD10 C50) during the period 1 January 1997 to 31 December 2011. They were included in the training population unless they had received a diagnosis of IBC prior to 1 January 1997 (even if another IBC was registered after 1 January 1997) or they had been diagnosed with an invasive cancer of a site other than IBC prior to the initial diagnosis of IBC (apart from non-melanoma skin cancer [ICD9 173; ICD10 C44] which was ignored). Also excluded were women who, at the time of their initial diagnosis of IBC or within three months of it, were diagnosed with an invasive cancer in the other breast (i.e. had bilateral IBC), as were women who were registered with a primary invasive cancer of another site at the same time as their initial diagnosis of IBC, or within three months of it. After these exclusions, the final training population included 48,192 women. For each woman included in the training population, the period of time considered ran from the date of initial diagnosis of IBC until the earliest of: date of death, date of emigration or 31 December 2011.

For the present project, the relevant outcomes recorded in the WMCIU database were listed (see Supplementary Table S1). It was noted that two or more outcomes were sometimes recorded on the same date for a woman. When this happened, one of them was usually more relevant than the other for the purposes of the algorithm. For example, if a confirmed distant metastasis to the liver was recorded on the same day as a recurrence in the ipsilateral breast, then the endpoint that is usually most relevant is distant metastasis. Therefore an order of priority for events was created and the outcomes in the WMCIU study were placed in this order such that, when two or more outcomes were recorded on the same date, the one recorded was the one of higher priority. Further details of the process are given elsewhere [12, 13]. The clinical outcomes recorded in the WMCIU database were then grouped into outcomes relevant to the present project: locoregional recurrence, distant recurrence, recurrence but type unknown, contralateral IBC, breast cancer death, non-breast-cancer malignancy, and non-breast-cancer death. The RCD sources available within NHS England with data fields relevant to these outcomes comprise: Cancer Outcomes and Services Dataset (COSD) [8, 14], Hospital Episode Statistics (HES) [15], Radiotherapy Dataset (RTDS) [16], Diagnostic Imaging Dataset (DIDS) [17], Cancer Waiting Times (CWT) [18], and Systemic Anti-Cancer Therapy dataset (SACT) [19] (Table 1). Within each data source, potentially informative items were identified, including type, date, and duration of various therapies, and pathological and radiological factors relevant to treatment decisions. We examined each of these data items and, taking into account standard clinical pathways (e.g. Supplementary Fig. S1 panel a), we developed an initial version of the algorithm that comprised a set of deterministic rules to identify each outcome as accurately as possible.

**Table 1:** Data sources and items considered during the training and validation of the recurrence algorithm.

Data Sources	Data Items
**Training data for initial development of algorithm**	
West Midlands Cancer Intelligence Unit project (WMCIU) [10, 11]	Database with detailed information on recurrences and other clinical outcomes for women living in the West Midlands Regional Health Authority and diagnosed with invasive breast cancer during the period 1 January 1997-31 December 2011
**Data collected routinely by NHS England for use in constructing the recurrence algorithm**	
Cancer Outcomes and Services Dataset (COSD): the national cancer registration dataset for England [8, 14]	*Patient*: Pseudonymized patient identifier, NHS number, date of birth, gender
	*Tumour*: Pseudonymized tumour identifier, site, morphology and behaviour of tumour, laterality
	*Diagnosis*: Date of diagnosis, basis of diagnosis, route to diagnosis, cancer care plan intent.
	*Treatment*: Event identifier, type of treatment event (surgery/radiotherapy/chemotherapy), date of event, details (dependent on type of event)
	*Death*: Date of death, ICD codes for underlying cause of death and any other causes mentioned on death certificate.
Hospital Episode Statistics (HES) [15]	*Diagnosis*: ICD diagnosis codes and dates of any hospital admissions
	*Treatment*: OPCS4 codes and dates of any procedures
Radiotherapy Dataset (RTDS) [16]	Date of recorded radiotherapy
Diagnostic Imaging Dataset (DIDS) [17]	Dates and type of imaging tests
Cancer Waiting Times (CWT) [18]	Dates and type of local and systemic treatment
Systemic Anti-Cancer Therapy (SACT) [19]	Dates and types of treatment available from COSD, HES and WMCIU from 2004 and from CWT from 2009. The SACT database started in 2014
**External validation data**	
AZURE randomised trial [20]	Date and type of recurrence
**Further Training data**	
150 women selected using stratified random sampling from AZURE randomised trial	Date and type of recurrence
**Internal validation data**	
AZURE randomised trial (excluding 150 women used for training)	Date and type of recurrence

ICD, International Classification of Diseases; OPCS4, statistical classification for clinical coding of hospital interventions and procedures undertaken by the NHS. The classification is mandatory for use by health care providers in England to support various forms of data collections for secondary uses.

### External validation, further training and subsequent internal validation of the algorithm

Independent data from the AZURE randomised trial [20] were obtained to undertake validation of the algorithm. This trial recruited women with stage II–III IBC during 2003–2006 and randomised them either to the intervention arm, where they received standard treatments plus 5 years of intravenous zoledronic acid, or to the control arm, where they received standard treatments only with no placebo. All women were invited to approximately 19 clinic visits over a 5-year period and then for 5 further annual clinic visits, so that the trial had 10-year follow-up overall [21]. After each visit, detailed follow-up forms were returned to the trial office. Any date of recurrence recorded in the trial was the date that it was first suspected, rather than the date it was confirmed.

**Fig. 1:**
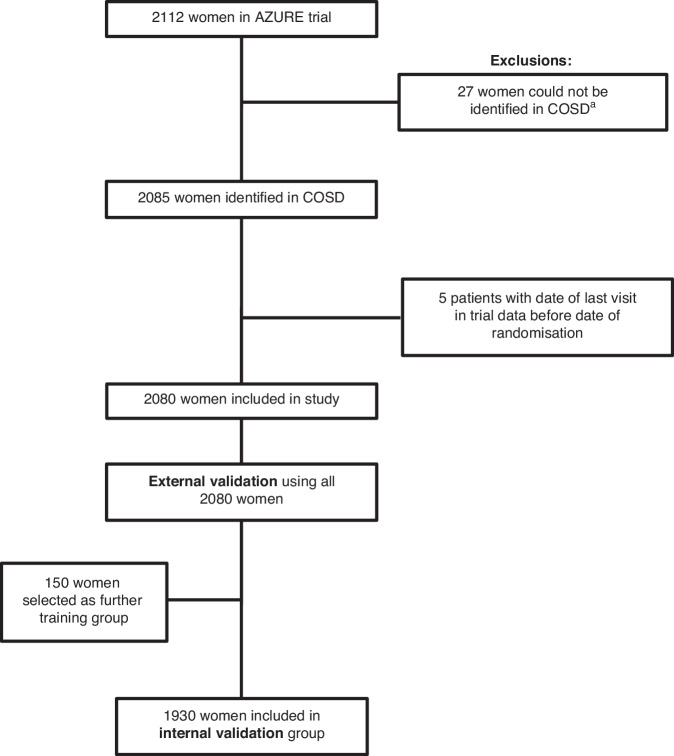
Derivation of validation and further training groups. ^a^ 9 were born in Wales; 1 could not be matched to COSD via NHS number or date of birth and name; 1 was not registered with invasive breast cancer (ICD-10 code C50); and 16 had registrations of invasive breast cancer that were classified as provisional status at the time of the initial data request. COSD Cancer Outcomes and Services Dataset.

For the present study, each of the 2112 women in the AZURE trial who were diagnosed in England was allocated a pseudonymised ID by the trial office. Information on dates, outcomes and pseudonymised IDs for these women was forwarded to Oxford, whilst identifying information and pseudonymised IDs, but no outcome information, were forwarded to NHS England. 2085 of the 2112 women were identified in COSD by NHS England and a further 5 were excluded because the recorded date of last follow-up visit in the trial data preceded the recorded date of randomisation (Fig. 1). For the remaining 2080, the clinical outcomes in the RCD were identified using the algorithm and forwarded to Oxford.

External validation of the algorithm was then carried out by comparing the outcomes derived from the RCD with those recorded in AZURE for the 2080 women. Outcomes identified in the RCD were included if they occurred between the date of randomisation in AZURE and the date of last follow-up in AZURE plus a 3-month window. The outcome of this exercise revealed differences between the outcomes reported in the RCD and those reported in AZURE for a considerable number of women. Therefore, further internal training of the algorithm was conducted. Three random samples of 50 women were selected: 50 where the first event in the RCD differed from that in AZURE; 50 where no outcome was reported in AZURE but an event was identified in the RCD; and 50 where the first event identified was the same in both methods. For this selection, an outcome of ‘recurrence type unknown’ in the RCD was taken to differ from the outcome in the AZURE data, as there were no recurrences of unknown type in the AZURE data. For each of these 150 women, all the information in the RCD was examined and, where appropriate, the algorithm was amended. Internal validation of the algorithm was then carried out by comparing the outcomes derived from the RCD with those recorded in AZURE for the remaining 1930 (i.e. 2080–150) women. The final algorithm rules are summarised in the Supplementary Materials (Supplementary Text S1 and Supplementary Tables S2-S3).

Analyses were carried out for the following outcomes: distant recurrence, locoregional recurrence, contralateral breast cancer, any recurrence, breast cancer mortality and all-cause mortality. Rules for including, censoring and ignoring different events are in Supplementary Table S4. Cumulative risks were calculated using the Kaplan-Meier method and the risks for the RCD and the AZURE trial were compared. The performance of the algorithm versus the data recorded in the AZURE trial was assessed using sensitivity, specificity, positive predictive value and negative predictive value [22]. When an outcome was identified in both the trial and the RCD, the dates in the two sources were compared. Calculations were conducted using Stata version 18.

This work was undertaken following research ethics committee approval (REC reference: 16/YH/0209) and approval from the Office of Data Release [23].

## Results

### Initial training

Among the 48,192 women diagnosed with early IBC and included in the WMCIU database, 26,028 had no event recorded and were not considered further. Of the 22,164 who did have an event recorded in the WMCIU, all but 1.5% [(160 + 24 + 152 + 1)/22,164] also had an event identified in the RCD (Table 2a). Of those whose first WMCIU event was locoregional recurrence, 93.4% [(4993 + 426)/5799] had either locoregional recurrence or recurrence of unknown type as first RCD event; of those whose first WMCIU event was distant recurrence 97.9% [(4927 + 31)/5063] had either distant recurrence or recurrence of unknown type as first RCD event; and of those whose first WMCIU event was recurrence of unknown type 92.0% [(2436 + 55 + 50)/2761] had locoregional or distant recurrence or recurrence of unknown type as first RCD event. Of the 8541 [(556 + 1921 + 2276 + 3788)] women whose first WMCIU event was contralateral IBC, death from breast cancer, non-breast malignancy, or non-breast-cancer death, 99.9% [(552 + 1921 + 2274 + 3788)/8541] had the same first RCD event.

**Table 2:** Comparison of first outcome events: (a) as recorded in the West Midlands Cancer Intelligence Unit (WMCIU) database project and as identified in the initial version of the algorithm based on routinely collected data (RCD); (b) as recorded in the AZURE external validation cohort of 2080 women and as identified by algorithm using the RCD after initial training with WMCIU data; and (c) as recorded in the AZURE internal validation group of 1930 women and as identified by algorithm using the RCD after further training using 150 women from the AZURE trial.

	Outcome from algorithm based on RCD								Total
	Locoregional recurrence	Distant recurrence	Recurrence, type unknown	Contralateral breast cancer	Death from breast cancer	Non-breast cancer malignancy	Non-breast-cancer death	No event recorded	
**a) Initial training**									
**Outcome in WMCIU database after initial development of algorithm using WMCIU database**									
Locoregional recurrence	**4993**	84	**426**	5	75	17	39	160	5799
Distant recurrence	3	**4927**	**31**	1	67	2	8	24	5063
Recurrence, type unknown	**55**	**50**	**2436**	8	40	5	15	152	2761
Contralateral breast cancer	3	0	0	**552**	0	0	0	1	556
Death from breast cancer	0	0	0	0	**1921**	0	0	0	1921
Non-breast cancer malignancy	2	0	0	0	0	**2274**	0	0	2276
Non-breast-cancer death	0	0	0	0	0	0	**3788**	0	3788
No event recorded^a^	—	—	—	—	—	—	—	26,028^a^	26,028
*Total*	*5056*	*5061*	*2893*	*566*	*2103*	*2298*	*3850*	*26,365*	*48,192*
**b) External validation**									
**Outcome in 2080 women in AZURE trial after initial development of algorithm using WMCIU database**									
Locoregional recurrence	**45**	26	**30**	4	0	0	1	3	109
Distant recurrence	78	**252**	**146**	21	14	1	2	6	520
Contralateral breast cancer	4	1	11	**23**	0	0	0	1	40
Death from breast cancer	1	9	1	0	**2**	0	0	0	13
Non-breast cancer malignancy	6	12	11	3	0	**35**	0	11	78
Non-breast-cancer death	3	5	7	1	1	1	**21**	1	40
No event recorded	129	135	339	43	0	3	0	**631**	1280
*Total*	*266*	*440*	*545*	*95*	*17*	*40*	*24*	*653*	*2080*
**c) Internal validation**									
**Outcome in 1930 women in AZURE trial after further training using 150 women from AZURE trial**									
Locoregional recurrence	**35**	20	**12**	10	3	2	1	5	88
Distant recurrence	56	**306**	**55**	12	18	3	1	6	457
Contralateral breast cancer	5	2	0	**26**	0	0	0	1	34
Death from breast cancer	1	8	0	0	**3**	0	1	0	13
Non-breast cancer malignancy	5	11	1	1	0	**40**	0	14	72
Non-breast-cancer death	3	5	2	2	3	0	**20**	1	36
No event recorded	93	72	70	31	0	8	0	**956**	1230
*Total*	*198*	*424*	*140*	*82*	*27*	*53*	*23*	*983*	*1930*

Outcomes shown are first outcome event of any type. Entries in the table in bold type indicate that the outcome using the algorithm based on the RCD and the outcome based on the WMCIU database or on data from the AZURE trial were judged to be in agreement.

^a^RCD information in women with no event recorded in the WMCIU database was not investigated.

### External validation

Among the 2080 women included in the AZURE trial, 68.8% [(45 + 30)/109] of women whose first AZURE event was locoregional recurrence had locoregional recurrence or recurrence of unknown type as first RCD event (Table 2 panel b); 76.5% [(252 + 146)/520] of women whose first AZURE event was distant recurrence had distant recurrence or recurrence of unknown type as first RCD event. There were no recurrences of unknown type in the AZURE data, and 57.5% (23/40) of women whose first AZURE event was contralateral breast cancer had contralateral breast cancer as their first event in the RCD database. 1280 women had no events in AZURE; 49.3% (631/1280) of them had no events in the RCD but, for 50.7% [(129 + 135 + 339 + 43 + 3)/1280], the algorithm suggested that an event had occurred. Cumulative risks and associated metrics for the external validation exercise are included in Supplementary Figures S2–S6 and Tables S5–S14.

### Further training

As there were considerable discrepancies between the outcomes recorded in the RCD after its initial development, further training was carried out using 150 women from the AZURE trial. Considering recurrence of unknown type in the RCD to be in agreement with locoregional and distant recurrences recorded in the AZURE data, the algorithm and the trial data were in disagreement for 77 women (Supplementary Table S15a, panel c). For these 77 women, the RCD data items were examined individually. Two main findings emerged. First, cosmetic and reconstructive surgery after the initial cancer surgery were incorrectly indicating locoregional or contralateral recurrence. The algorithm was therefore refined with the creation of new rules to prevent this. Second, the algorithm was incorrectly interpreting the administration of zoledronic acid in the trial intervention arm as palliative chemotherapy, indicating recurrence (Supplementary Table S15a, panel b). The extent to which this occurred was reduced by creating rules based on the zoledronic acid administration protocol in AZURE. After completion of this further training, the algorithm was re-run on the same sample of 150 women. The number of women where the RCD and the trial data were not in agreement had reduced from 77 to 39 (i.e. 26% Supplementary Table S15b, panel c).

### Internal Validation: distant recurrence

**Fig. 2:**
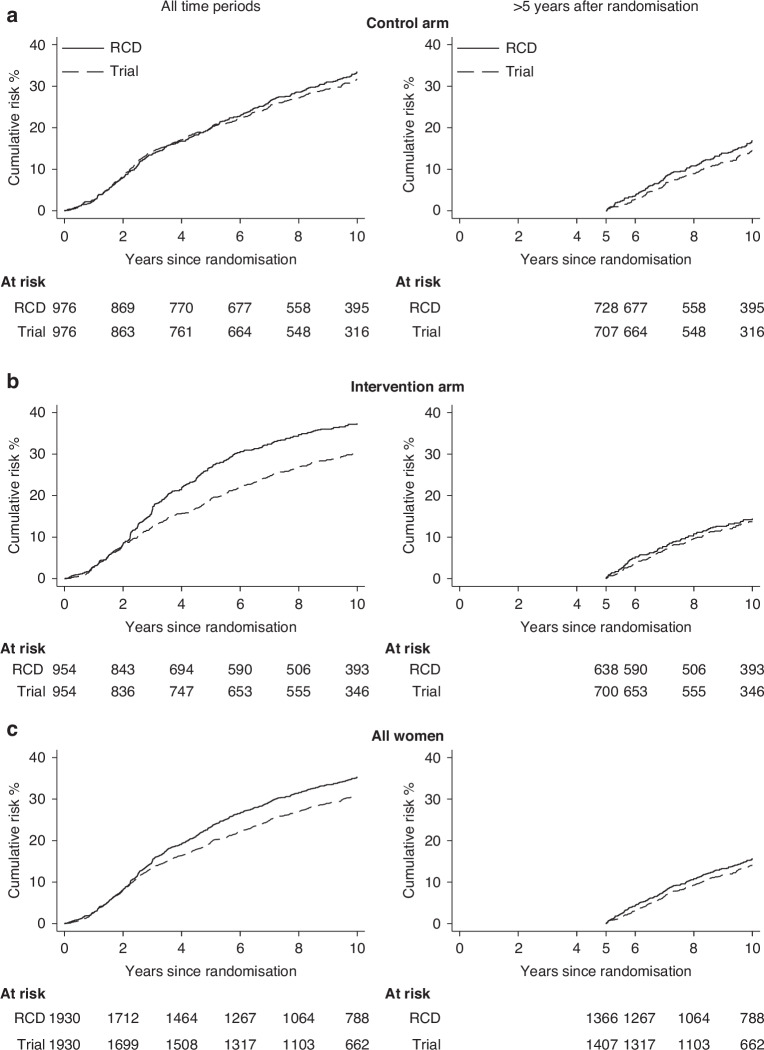
Internal validation exercise: Cumulative risk of distant recurrence in AZURE trial (dashed lines) and in the routinely collected data (RCD) (solid lines). Analyses are by separate randomisation arm (**a** and **b**), and for all 1930 women included in the internal validation group (**c**). The outcome of distant recurrence is defined in Table S4. Plotted values at 1-, 3-, 5- and 10-years (and 95% confidence intervals) are in Table S17.

Based on the remaining 1930 women in the AZURE trial, the RCD and the trial data were in agreement for 75.3% (i.e. 1453/1930) of women (Supplementary Table S16, panel c). The cumulative risks of distant recurrence were 23.2% and 35.8% at 5 and 10 years respectively using the RCD-based algorithm, whilst in the trial data risks were lower, at 19.5% and 32.1% respectively (Fig. 2c left panel; Supplementary Table S17). When the trial arms were examined separately, the difference was smaller for the control arm (5 years: RCD 19.9%, trial 20.0%; 10 years: RCD 33.8%, trial 32.7%. Figure 2a left panel) than for the intervention arm (5 years: RCD: 26.7%, trial 19.0%; 10 years: RCD 37.9%, trial 31.4%. Figure 2b left panel).

The trial intervention lasted 5 years and, after this, the cumulative risk curves for the RCD and the trial data were approximately parallel, indicating that annual rates were similar in the two datasets (Fig. 2b left panel). The difference between the curves during years 0–5 suggested that, despite further training of the algorithm, administration of zoledronic acid in the trial intervention arm was still being incorrectly identified as palliative chemotherapy for some women and interpreted as distant recurrence. When the analysis was repeated omitting the first 5 years after randomisation the cumulative risk of distant recurrence during the period 5–10 years after randomisation was similar in the RCD and the trial data both overall (RCD 16.4%, trial 15.6%) and separately by trial arm (control: RCD 17.4%, trial 15.8%; intervention: RCD 15.2%, trial 15.4%; Fig. 2 right panels Supplementary Table S17).

The sensitivity of the RCD-based algorithm for distant recurrence was 95.6% (95% CI 93.8–97.3) overall (Table 3), with little difference between trial arms (control: 95.7%, 95% CI 93.3–98.1; intervention: 95.4%, 95% CI 92.9–98.0). Considering just the period >5 years from randomisation, sensitivity decreased slightly (overall: 89.4%, 95% CI 84.7–94.2; control: 89.9%, 95% CI 83.6–96.2; intervention: 88.9%, 95% CI 81.6–96.1). Overall, the specificity of the RCD-based algorithm for distant recurrence was 91.9% (95% CI 90.5–93.4), and was higher in the control arm (95.4%, 95% CI 93.9–97.0) than the intervention arm (88.4%, 95% CI 86.0–90.8). More than 5 years from randomisation, the overall specificity increased to 98.0% (95% CI 97.2–98.8), and was similar in both trial arms (control: 98.2%, 95% CI 97.1–99.2; intervention: 97.8%, 95% CI 96.6–99.0). Results were very similar when the data were subdivided by age at randomisation, stage, or grade (Supplementary Figs S7–S9). Overall, 73.8% of distant recurrences identified by the RCD were within 6 months of those recorded in the trial and 84.6% were within 12 months. Considering just events >5 years after randomisation these percentages rose to 87.5% and 94.4%.

**Table 3:** Internal validation exercise: Agreement of routinely collected data (RCD) and AZURE trial data for distant recurrence.

Distant recurrence	Analysis period and trial arm					
	All time periods			>5 years after randomisation^a^		
	Control (*N* = 976)	Intervention (*N* = 954)	Total (*N* = 1930)	Control (*N* = 976)	Intervention (*N* = 954)	Total (*N* = 1930)
			**No.**			
Event in both datasets	267	251	518	80	64	144
Event only in trial data	12	12	24	9	8	17
Event only in RCD	32	80	112	11	12	23
No event in either dataset	665	611	1276	593	536	1129
**Censored before analysis period**						
Only in trial data	0	0	0	35	18	53
Only in RCD	0	0	0	14	80	94
In both datasets	0	0	0	234	236	470
**Time difference when event present in both datasets**			**No. (%)**			
<6 months	207 (77.5%)	175 (69.7%)	382 (73.8%)	69 (86.2%)	57 (89.1%)	126 (87.5%)
6–12 months	26 (9.8%)	30 (12.0%)	56 (10.8%)	8 (10.0%)	2 (3.1%)	10 (6.9%)
>1 year	34 (12.7%)	46 (18.3%)	80 (15.4%)	3 (3.8%)	5 (7.8%)	8 (5.6%)
**Performance measures**^**b**^			**% (95% CI)**			
*All time periods*						
Sensitivity	95.7 (93.3, 98.1)	95.4 (92.9, 98.0)	95.6 (93.8, 97.3)	89.9 (83.6, 96.2)	88.9 (81.6, 96.1)	89.4 (84.7, 94.2)
Specificity	95.4 (93.9, 97.0)	88.4 (86.0, 90.8)	91.9 (90.5, 93.4)	98.2 (97.1, 99.2)	97.8 (96.6, 99.0)	98.0 (97.2, 98.8)
PPV	89.3 (85.8, 92.8)	75.8 (71.2, 80.4)	82.2 (79.2, 85.2)	87.9 (81.2, 94.6)	84.2 (76.0, 92.4)	86.2 (81.0, 91.5)
NPV	98.2 (97.2, 99.2)	98.1 (97.0, 99.2)	98.2 (97.4, 98.9)	98.5 (97.5, 99.5)	98.5 (97.5, 99.5)	98.5 (97.8, 99.2)
*Within 6 months of trial data*						
Sensitivity	74.2 (69.1, 79.3)	66.5 (60.8, 72.2)	70.5 (66.6, 74.3)	77.5 (68.9, 86.2)	79.2 (69.8, 88.5)	78.3 (71.9, 84.6)
Specificity	95.4 (93.9, 97.0)	88.4 (86.0, 90.8)	91.9 (90.5, 93.4)	98.2 (97.1, 99.2)	97.8 (96.6, 99.0)	98.0 (97.2, 98.8)
PPV	86.6 (82.3, 90.9)	68.6 (62.9, 74.3)	77.3 (73.6, 81.0)	86.2 (78.7, 93.8)	82.6 (73.7, 91.6)	84.6 (78.8, 90.4)
NPV	90.2 (88.1, 92.4)	87.4 (85.0, 89.9)	88.9 (87.2, 90.5)	96.7 (95.3, 98.1)	97.3 (95.9, 98.6)	97.0 (96.0, 98.0)

Calculations performed for all 1930 women in the internal validation group. The outcome of distant recurrence is defined in Table S4.

PPV, Positive predictive value; NPV, Negative predictive value.

^a^Analysis period starts at 5 years after randomisation.

^b^Sensitivity defined as percentage of women with the outcome who are correctly identified as such in the RCD. Specificity defined as percentage of women without the outcome who are correctly identified as such in the RCD. PPV defined as percentage of women identified in the RCD as having the outcome who do in fact have it. NPV defined as percentage of women identified in the RCD as not having the outcome who do not in fact have it. Women censored before analysis period excluded.

### Internal validation: Locoregional recurrence and contralateral breast cancer

Based on 1930 women, the cumulative risks of locoregional recurrence being recorded before any distant recurrence were 10.2% and 12.3% at 5 and 10 years respectively using the RCD-based algorithm, whilst for the trial data these risks were much lower, at 4.2% and 5.7% respectively, with large differences occurring in both control and intervention arms (Supplementary Fig. S10 and Table S18). These big differences were mainly due to differences occurring during the first year following randomisation and differences were much smaller when cumulative risks starting from either one year or 5 years after randomisation were considered. The specificity of the RCD to detect locoregional recurrence was 91.0% for follow-up starting on date of randomisation, and 97.2% and 98.7% for follow-up starting at one and 5 years following randomisation. The corresponding sensitivities were 45.5%, 47.1% and 58.3% respectively (Table S19).

For contralateral breast cancer, the cumulative risks were 3.8% and 6.1% at 5 and 10 years using the RCD-based algorithm, and 0.9% and 2.8% in the trial with little difference in agreement between the RCD and trial by trial arm (Supplementary Fig. S11 and Table S20). As for locoregional recurrence, the discrepancy was higher in the early period following randomisation, and agreement improved in the period >5 years following randomisation, with sensitivity and specificity of 88.2% and 99.4% after 5 years, compared with 76.5% and 97.0% for all time periods after randomisation (Supplementary Table S21).

### Internal validation: any recurrence

**Fig. 3:**
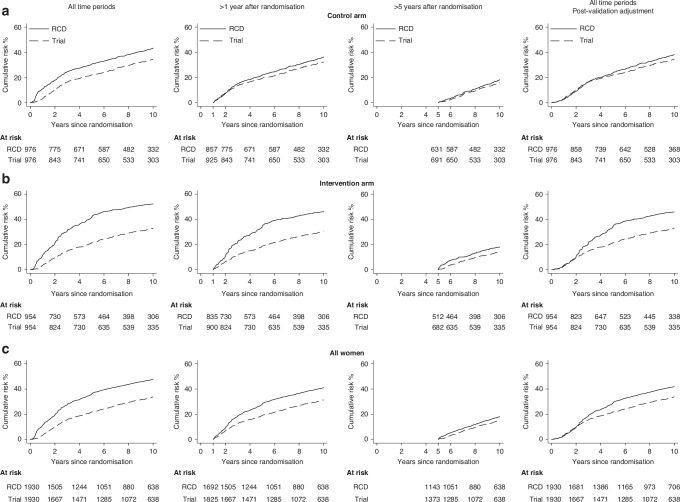
Internal validation exercise: Cumulative risk of any recurrence in AZURE trial (dashed lines) and in routinely collected data (RCD) (solid lines). Analyses are by separate randomisation arm (**a**, **b**), and for all 1930 women included in the internal validation group (**c**). The outcome of any recurrence is defined in Table S4. Post-validation adjustment ignores events reported in the RCD as locoregional recurrence, recurrence of unknown type or contralateral breast cancer during the first year after diagnosis. Plotted values at 1-, 3-, 5- and 10-years (and 95% confidence intervals) are given in Table S22.

Based on the same 1930 women in the AZURE trial, the cumulative risk of any recurrence increased more rapidly during the first year after randomisation in the RCD than in the trial data (Fig. 3 first column). After this first year, the absolute difference between the cumulative risks in the two datasets remained constant with increasing time since randomisation for the control arm but, for the intervention arm, the difference increased until 5 years after randomisation. When the analysis was repeated omitting the first year after randomisation, the 1–10-year cumulative risk of any recurrence for women in the control arm was 36.6% using the RCD and 33.4% in the trial data, while for the intervention arm, the corresponding risks were 46.8% using the RCD and 31.6% in the trial data (Fig. 3 second column; Supplementary Table S22). Repeating the analysis omitting the first 5 years after randomisation, the 5–10-year cumulative risks of any recurrence for all women were 19.0% using the RCD and 16.6% in the trial data, with similar values for the two randomisation arms (Fig. 3 third column).

Considering all 1930 women and follow-up from date of randomisation, the sensitivity of the RCD-based algorithm for any recurrence was 96.6% (95% CI 95.2–98.1) (Table 4), with little difference between trial arms (control: 96.7%, 95% CI 94.7–98.7; intervention: 96.5%, 95% CI 94.4–98.6). Sensitivity was similar when considering follow-up from one year or from 5 years since randomisation. Considering follow-up from date of randomisation, the specificity of the RCD-based algorithm for any recurrence was 77.7% (95% CI 75.4–79.9) overall, and it was higher in the control (85.2%, 95% CI 82.6–87.9) than the intervention arm (70.0%, 95% CI 66.5–73.5). Considering just follow-up from one year after randomisation, specificity was greater, but there was still a difference between the trial arms (control: 92.2, 95% CI 90.1–94.4; intervention: 77.1, 95% CI 73.7–80.5). However, when considering just 5 years from randomisation, specificity was high in both trial arms (control: 97.0%, 95% CI 95.5–98.4; intervention: 94.3%, 95% CI 92.1–96.5). Considering follow-up from time of randomisation, 71.0% of all recurrences identified by the RCD were within 6 months of those recorded in the trial, rising to 78.3% within 12 months. Considering follow-up just from 5 years after randomisation these proportions increased to 93.4% and 97.0%.

**Table 4:** Internal validation exercise: Agreement of routinely collected data (RCD) and AZURE trial data for any recurrence.

Any recurrence	Analysis period and trial arm											
	All time periods			>1 year after randomisation^a^			>5 years after randomisation^a^			All time periods with post-validation adjustment^b^		
	Control (*N* = 976)	Intervention (*N* = 954)	Total (N = 1930)	Control (*N* = 976)	Intervention (*N* = 954)	Total (*N* = 1930)	Control (*N* = 976)	Intervention (*N* = 954)	Total (*N* = 1930)	Control (*N* = 976)	Intervention (*N* = 954)	Total (*N* = 1930)
						**No**.						
Event in both datasets	295	277	572	230	217	447	79	58	137	294	276	570
Event only in trial data	10	10	20	8	9	17	5	2	7	11	11	22
Event only in RCD	99	200	299	47	135	182	16	25	41	68	169	237
No event in either dataset	572	467	1039	558	455	1013	509	414	923	603	498	1101
**Censored before analysis period**												
Only in trial data	0	0	0	14	19	33	22	13	35	0	0	0
Only in RCD	0	0	0	82	84	166	82	183	265	0	0	0
In both datasets	0	0	0	37	35	72	263	259	522	0	0	0
**Time difference when event present in both datasets**						**No. (%)**						
<6 months	217 (73.5%)	189 (68.2%)	406 (71.0%)	189 (82.2%)	165 (76.0%)	354 (79.2%)	73 (92.4%)	55 (94.8%)	128 (93.4%)	237 (80.6%)	198 (71.7%)	435 (76.3%)
6–12 months	20 (6.8%)	22 (8.0%)	42 (7.3%)	16 (6.9%)	15 (6.9%)	31 (6.9%)	3 (3.8%)	2 (3.5%)	5 (3.6%)	19 (6.5%)	21 (7.6%)	40 (7.0%)
>1 year	58 (19.7%)	66 (23.8%)	124 (21.7%)	25 (10.9%)	37 (17.1%)	62 (13.9%)	3 (3.8%)	1 (1.7%)	4 (3.0%)	38 (12.9%)	57 (20.7%)	95 (16.7%)
**Performance measures**^**c**^						**% (95% CI)**						
*All time periods*												
Sensitivity	96.7 (94.7, 98.7)	96.5 (94.4, 98.6)	96.6 (95.2, 98.1)	96.6 (94.3, 98.9)	96.0 (93.5, 98.6)	96.3 (94.6, 98.0)	94.0 (89.0, 99.1)	96.7 (92.1, 100)	95.1 (91.6, 98.7)	96.4 (94.3, 98.5)	96.2 (93.9, 98.4)	96.3 (94.8, 97.8)
Specificity	85.2 (82.6, 87.9)	70.0 (66.5, 73.5)	77.7 (75.4, 79.9)	92.2 (90.1, 94.4)	77.1 (73.7, 80.5)	84.8 (82.7, 86.8)	97.0 (95.5, 98.4)	94.3 (92.1, 96.5)	95.7 (94.5, 97.0)	89.9 (87.6, 92.1)	74.7 (71.4, 78.0)	82.3 (80.2, 84.3)
PPV	74.9 (70.6, 79.2)	58.1 (53.6, 62.5)	65.7 (62.5, 68.8)	83.0 (78.6, 87.5)	61.6 (56.6, 66.7)	71.1 (67.5, 74.6)	83.2 (75.6, 90.7)	69.9 (60.0, 79.7)	77.0 (70.8, 83.2)	81.2 (77.2, 85.2)	62.0 (57.5, 66.5)	70.6 (67.5, 73.8)
NPV	98.3 (97.2, 99.3)	97.9 (96.6, 99.2)	98.1 (97.3, 98.9)	98.6 (97.6, 99.6)	98.1 (96.8, 99.3)	98.3 (97.6, 99.1)	99.0 (98.2, 99.9)	99.5 (98.9, 100)	99.2 (98.7, 99.8)	98.2 (97.2, 99.3)	97.8 (96.6, 99.1)	98.0 (97.2, 98.9)
*Within 6 months of trial data*												
Sensitivity	71.1 (66.1, 76.2)	65.9 (60.4, 71.3)	68.6 (64.8, 72.3)	79.4 (74.3, 84.5)	73.0 (67.2, 78.8)	76.3 (72.4, 80.2)	86.9 (79.7, 94.1)	91.7 (84.7, 98.7)	88.9 (83.8, 94.0)	77.7 (73.0, 82.4)	69.0 (63.6, 74.3)	73.5 (69.9, 77.0)
Specificity	85.2 (82.6, 87.9)	70.0 (66.5, 73.5)	77.7 (75.4, 79.9)	92.2 (90.1, 94.4)	77.1 (73.7, 80.5)	84.8 (82.7, 86.8)	97.0 (95.5, 98.4)	94.3 (92.1, 96.5)	95.7 (94.5, 97.0)	89.9 (87.6, 92.1)	74.7 (71.4, 78.0)	82.3 (80.2, 84.3)
PPV	68.7 (63.6, 73.8)	48.6 (43.6, 53.6)	57.6 (53.9, 61.2)	80.1 (75.0, 85.2)	55.0 (49.4, 60.6)	66.0 (62.0, 70.1)	82.0 (74.0, 90.0)	68.8 (58.6, 78.9)	75.7 (69.3, 82.2)	77.7 (73.0, 82.4)	54.0 (48.9, 59.1)	64.7 (61.1, 68.3)
NPV	86.7 (84.1, 89.3)	82.7 (79.5, 85.8)	84.8 (82.8, 86.8)	91.9 (89.8, 94.1)	88.2 (85.4, 91.0)	90.2 (88.5, 91.9)	97.9 (96.6, 99.1)	98.8 (97.8, 99.8)	98.3 (97.5, 99.1)	89.9 (87.6, 92.1)	84.8 (81.9, 87.7)	87.5 (85.7, 89.3)

Calculations performed for all 1930 women in the internal validation group. The outcome of any recurrence is defined in Table S4.

PPV, Positive predictive value; NPV, Negative predictive value.

^a^Analysis period starts at 1 or 5 years after randomisation.

^b^Post-validation adjustment ignores events reported in the RCD as locoregional recurrence, recurrence of unknown type or contralateral breast cancer during the first year after diagnosis.

^c^Performance measures defined in footnote of Table 3.

### Internal validation: breast cancer mortality and all-cause mortality

**Fig. 4:**
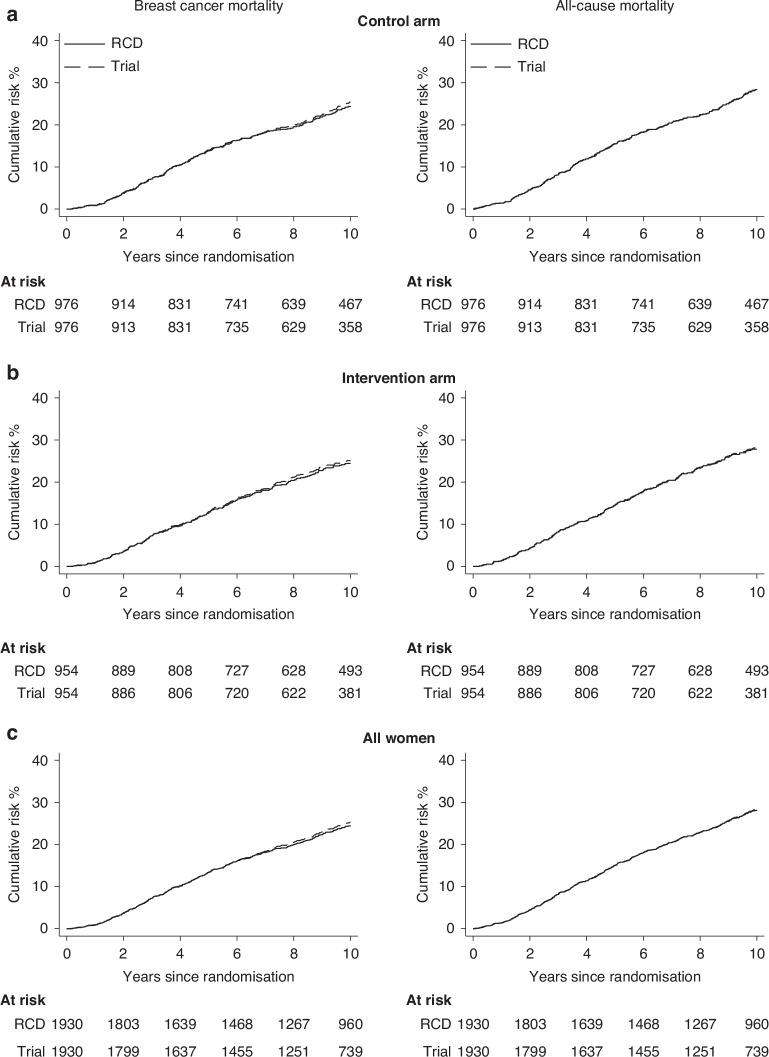
Internal validation exercise: Cumulative risks of breast cancer mortality (left) and all-cause mortality (right) in AZURE trial (dashed lines) and in routine data (solid lines). Analyses are by randomisation arm (**a**, **b**), and for all 1930 women included in the internal validation group (**c**). The outcomes of breast cancer mortality and all-cause mortality are defined in Table S4. Plotted values at 1-, 3-, 5- and 10-years (and 95% confidence intervals) are in Table S23.

For breast cancer mortality and all-cause mortality, cumulative risks were almost identical in the RCD and the trial data, regardless of trial arm (Fig. 4). Breast cancer mortality risks at 5 and 10 years after randomisation were 13.3% and 24.7% in the RCD and 13.4% and 26.6% in the trial data respectively (Supplementary Table S23). Of the 445 women recorded as dying from breast cancer in the AZURE trial, 400 were identified as having a distant recurrence prior to their death based on the RCD, compared with 418 women in the trial data. The distribution of times from randomisation to distant metastasis and from distant metastasis to death were similar in the RCD and in the AZURE trial data (Supplementary Fig. S12). The all-cause mortality risks at 5 and 10 years after randomisation were 15.0% and 28.5% in the RCD and 15.0% and 29.8% in the trial data respectively. Sensitivities for breast cancer mortality and all-cause mortality were 94.4% and 99.8% respectively, while the specificity was >99% for both endpoints (Supplementary Table S24).

### Post-validation adjustment: any recurrence

After completing both external and internal validation exercises, the individual events reported in the RCD were examined and compared with those in the trial. In the RCD, irrespective of randomisation arm, cosmetic and symmetrising surgical procedures occurring up to a year after IBC diagnosis were being incorrectly interpreted as locoregional recurrences or contralateral breast cancers. The analysis of any recurrence was therefore repeated ignoring events reported in the RCD as locoregional recurrence, recurrence of unknown type or contralateral breast cancer during this period. With this post-validation adjustment, the cumulative risks in the control arm at 5 and 10 years after randomisation were similar in the RCD and trial data (RCD: 23.7% and 38.6%; trial 22.1% and 35.6%) (Fig. 3 right column; Supplementary Table S22). The sensitivity of the RCD to detect any recurrence in the control arm remained high, at 96.4% while its specificity improved to 89.9% (Table 4). Examination of the events in the RCD also revealed that, in the intervention arm, misinterpretation of the trial intervention as distant recurrence, as described above, was the likely reason for the difference between the two datasets up to 5 years following randomisation.

## Discussion

In this study, an algorithm to identify recurrences among women diagnosed with early IBC using just the RCD that are currently available within NHS England has been developed and its performance evaluated using data from the AZURE trial, which enrolled women shortly after a diagnosis of IBC and followed them by means of clinic visits for ten years. For distant recurrence, the algorithm had good sensitivity and specificity, while for any recurrence sensitivity was good but specificity was lower. This was mainly due to the fact that during the first five years of follow-up the algorithm identified irrelevant events incorrectly as locoregional recurrences and contralateral breast cancers. During the second five years of follow-up the algorithm performed well for both distant recurrence and any recurrence. The ability of the routinely collected data to identify deaths from breast cancer and from all causes was satisfactory throughout the ten-year follow-up period.

In its present form, the algorithm could be used as an aid to randomised trials in either of two ways. Firstly, active follow-up of all trial participants could be conducted for the first 5 years of the trial. Secondly, for the first five years after randomisation, events identified in the RCD could be checked with the relevant hospital. With either of these options, the RCD could be used on its own for follow-up beyond five years. Whilst neither solution automates trial follow-up entirely, the implementation of either one would make the follow-up in trials of breast cancer treatments substantially cheaper and more efficient than it is at the moment, where active follow-up needs to be carried out for all participating individuals throughout a trial. This is particularly important in breast cancer, where recurrences can occur for at least two decades after the original primary, and so follow-up also needs to last for several decades.

### Distant recurrence

During the first five years of follow-up, the algorithm performed well for distant recurrence among women randomised to the control arm in the AZURE trial (i.e. standard treatments only). In the intervention arm, however, women received bisphosphonate in the form of zoledronic acid intravenously for a period of five years and, based on HES data, the algorithm could not distinguish this from chemotherapy. Bisphosphonates are no longer delivered intravenously for most patients with early IBC, but several newer systemic treatments are used and others may be introduced over time and approved into national treatment guidelines. From 2014, specific drug names have been recorded in the SACT database. Their inclusion in the algorithm should be straightforward and can be expected to improve the algorithm for the endpoint of distant recurrence compared with its performance in the present study, thus enabling reliable information on distant recurrence in routine care to be produced on a nationwide basis in the near future.

### Locoregional recurrence and contralateral breast cancer

Although the RCD-based algorithm had adequate specificity for locoregional recurrences and contralateral breast cancers in the first few years after diagnosis, its sensitivity was poor. This mainly arose from the difficulty of differentiating reconstructive operations and oncoplastic procedures (e.g. symmetrising contralateral surgery) from true locoregional recurrences or contralateral cancers. Future work to improve the identification of locoregional and contralateral breast cancers is needed, using training and validation datasets where locoregional and contralateral recurrences are fully recorded.

### Any recurrence

Despite the difficulties in the accurate identification of locoregional recurrences, the RCD-based algorithm had high sensitivity for women in both trial arms throughout follow-up. Its specificity was much higher for women in the control arm of the AZURE trial than in the intervention arm, especially after the first year of follow-up. Given the likely improvement in the specificity for identifying distant recurrences mentioned above, the performance of the endpoint of any recurrence may be sufficient for many epidemiological purposes in the near future.

### The growing need for information on recurrence

Three factors have influenced the recent landscape of breast cancer clinical research. Firstly, women entering clinical trials are surviving for longer than previously, and therefore, longer follow-up is needed, with greater emphasis on endpoints determining quality of life, such as breast cancer recurrence. Second, several side-effects of IBC treatments only become clinically important over a decade following cancer diagnosis. Hence, in modern clinical studies, follow-up needs to be long enough for the incidence of these side effects to be compared with the rate at which recurrence occurs, so that the long-term net benefit of the various treatment options can be evaluated. A third consideration is that improvement in IBC survival has been achieved through a series of small advances in treatment. It is likely that this approach will continue to be informative in the future and so larger trials will be needed to provide adequate power to detect small treatment improvements. For all these reasons, use of RCD for endpoint ascertainment, as described in this study, could help make future randomised trials in IBC more cost-effective in England, enabling larger and longer-term studies to be conducted.

### Applications of recurrence information from routinely collected data

Due to the lack of reliable recurrence information, population-level descriptive and analytical epidemiology in women with breast cancer in England has been confined to examining factors associated with survival. This means that policies on optimal follow-up surveillance after breast cancer and contemporary clinical decision-aid tools used for clinical management make recommendations centred on survival information alone. However, survival from breast cancer has improved markedly in recent years [3], making other concerns of survivorship, such as cancer recurrence, more important than previously. The approach described in this paper offers a potential mechanism to provide this information in the future, albeit with certain caveats at present.

Algorithms have been proposed to identify breast cancer recurrences from RCD in cancer registries in several other countries [24–29], but the present study represents the most comprehensive approach in England to date. Previous work in England, published in 2017, comparing RCD with randomised trial data did not yield satisfactory results (see Supplementary Table S25 for details) [4] and concluded that routine data quality required improvement before it could be used for randomised trials. Another concern raised in relation to using RCD in England for such purposes has been administrative delays in processing data before it becomes available for clinical research [4]. However, NHS DigiTrials [30], which was developed during the COVID pandemic, has recently processed and delivered data efficiently to approved researchers and provides an example of a platform through which timely recurrence information generated by the algorithm could be made available to researchers in the future.

### Future work

As mentioned above, it should require only a minor change in the algorithm to include the names of specific drugs from 2014, thereby improving its performance substantially with respect to distant recurrences. There is also the potential to improve the algorithm’s performance with respect to locoregional recurrences and contralateral breast cancers with further training and validation using additional datasets. It may also be possible to include more data items from newer datasets, such as DIDS, and to incorporate new datasets such as the Primary Care Prescription Database [31]. In principle, the addition of any new future population level database or changes to treatment rules in the algorithm could well improve its performance across all recurrence types. Implementation of any such changes would, however, require additional training of the algorithm and, if possible, an independent validation exercise to review its performance with these changes. New population-level datasets or treatments are not introduced frequently, so once the algorithm is working satisfactorily, repeat validation is not likely to be required often.

## Conclusion

This study has demonstrated the potential of routinely collected data to provide information on recurrences in women diagnosed with early breast cancer. It thus provides evidence that routinely collected data can be used in descriptive epidemiological studies and in clinical trials throughout England. Further work is, however, required to incorporate drug names from the SACT database into the algorithm and to improve the ascertainment of locoregional recurrences and contralateral breast cancers in the first few years after diagnosis.

## Supplementary information

Supplementary Materials

## Data Availability

De-personalised study data may be made available on request to accredited researchers who submit a proposal that is approved by NHS England’s Data Access Request Service (DARS). Further information on the use of the algorithm is available at https://www.ctsu.ox.ac.uk/research/benefits-and-risks-of-cancer-treatment/research/identification-of-recurrences-in-women-with-early-breast-cancer.
