# Supplementary material for: Identification of recurrences in women diagnosed with early invasive breast cancer using routinely collected data in England
Source: BJC Rep. 2025 May 28;3:39. doi: 10.1038/s44276-025-00154-1 (PMC12119862; doi:10.1038/s44276-025-00154-1)
Supplement: Supplementary file 1 — Supplementary Materials [file 44276_2025_154_MOESM1_ESM.pdf]

Identification of recurrences in women diagnosed with early invasive breast cancer using routinely collected data in England

Supplementary Materials

Derivation of algorithm

Table S1	Clinical outcomes recorded in West Midlands Cancer Intelligence Unit (WMCIU) database and outcomes for algorithm based on routinely collected data (RCD).	3
Figure S1	Example of coding rule used to help differentiate breast cancer recurrence from standard clinical care pathway based on the period of time between certain procedures.	4
Text S1	Summary of classifications used to derive outcomes for algorithm based on routinely collected data.	5
Tables S2a-S2g	Summaries of code groupings used to derive outcomes for algorithm based on routinely collected data.	12
Table S3	For women registered as stage VI (unknown stage), ICD-10 codes C77-C79 from HES data (within 3 months of diagnosis of breast cancer) were used to determine cancer stage.	21
Table S4	Summary of the clinical events used for each of the different analysis outcomes considered.	22

External validation

Figure S2	External validation exercise: Cumulative risk of distant recurrence	23
Table S5	External validation exercise: Number of events and cumulative risks of distant recurrence	24
Table S6	External validation exercise: Agreement measures between RCD and AZURE trial data for distant recurrence	25
Figure S3	External validation exercise: Cumulative risk of any recurrence	26
Table S7	External validation exercise: Number of events and cumulative risks of any recurrence	27
Table S8	External validation exercise: Agreement measures between RCD and AZURE trial data for any recurrence	28
Figure S4	External validation exercise: Cumulative risk of locoregional recurrence	29
Table S9	External validation exercise: Number of events and cumulative risks of locoregional recurrence	30
Table S10	External validation exercise: Agreement measures between RCD and AZURE trial data for locoregional recurrence	31
Figure S5	External validation exercise: Cumulative risk of contralateral breast cancer	32
Table S11	External validation exercise: Number of events and cumulative risks of contralateral breast cancer	33

Table S12	External validation exercise: Agreement measures between RCD and AZURE trial data for contralateral breast cancer	34
Figure S6	External validation exercise: Cumulative risk of breast cancer mortality and all-cause mortality	35
Table S13	External validation exercise: Number of events and cumulative risks of breast cancer mortality and all-cause mortality	36
Table S14	External validation exercise: Agreement measures between RCD and AZURE trial data for breast cancer mortality and all-cause mortality	37

Further training

Tables S15a-b	Training exercise before and after further training: Comparison of first outcome events recorded in the AZURE training sample of 150 women and as identified by algorithm using routinely collected data, by trial arm.	38

Internal validation

Table S16	Internal validation exercise: Comparison of first outcome events recorded in AZURE trial and as identified by algorithm using routinely collected data.	40
Table S17	Internal validation exercise: Number of events and cumulative risks of distant recurrence	41
Figure S7	Internal validation exercise: Cumulative risk of distant recurrence by age at diagnosis	42
Figure S8	Internal validation exercise: Cumulative risk of distant recurrence by stage	43
Figure S9	Internal validation exercise: Cumulative risk of distant recurrence by grade	44
Figure S10	Internal validation exercise: Cumulative risk of locoregional recurrence	45
Table S18	Internal validation exercise: Number of events and cumulative risks of locoregional recurrence	46
Table S19	Internal validation exercise: Agreement measures between RCD and AZURE trial data for locoregional recurrence	47
Figure S11	Internal validation exercise: Cumulative risk of contralateral breast cancer	48
Table S20	Internal validation exercise: Number of events and cumulative risks of contralateral breast cancer	49
Table S21	Internal validation exercise: Agreement measures between RCD and AZURE trial data for contralateral breast cancer	50
Table S22	Internal validation exercise: Number of events and cumulative risks of any recurrence	51
Table S23	Internal validation exercise: Number of events and cumulative risks of breast cancer mortality and all-cause mortality	52
Figure S12	Internal validation exercise: Distribution of time until distant metastasis and then to death from breast cancer for 445 women recorded as dying from breast cancer in trial data.	53
Table S24	Internal validation exercise: Agreement measures between RCD and AZURE trial data for breast cancer mortality and all-cause mortality	54

Previous work

Table S25	Previous work in England comparing outcomes in routine data held by the former National Cancer Data Repository with those reported in the TACT (Taxotere and Adjuvant ChemoTherapy) trial.	55

Table S1. Clinical outcomes recorded in West Midlands Cancer Intelligence Unit (WMCIU) database and outcomes for algorithm based on routinely collected data (RCD).

**Outcomes in WMCIU database** listed in order of priority for algorithm development	**Code**	**Outcomes for algorithm based on RCD**
Not eligible for study (e.g. not resident in WMCIU region)	A[a]	-
Death certificate registration only or date of diagnosis same as date of death	B2[a]	-
Stage 4 disease, metastases or palliative treatment <3 months after diagnosis	B1[a]	-
Death from breast cancer with previous breast specific surgery	C01a	Death from breast cancer
Death from breast cancer without known breast specific surgery	C01b	
Confirmed distant metastases to liver/lung/bone/brain	C03a(i)	Distant recurrence
Confirmed distant metastasis to other locations	C03a(ii)	
Record of palliative care	C03a(iii)	
Unconfirmed distant metastasis to liver/lung/bone/brain	C03b	
Contralateral breast cancer	C07	Contralateral breast cancer
Recurrence in ipsilateral regional lymph nodes	C04a	Locoregional recurrence
Recurrence in regional lymph nodes with no laterality recorded	C04b	
Recurrence in ipsilateral breast	C05	
Recurrence, type unknown	C06	Recurrence, type unknown
Microscopically confirmed primary cancer of liver/lung/brain/bone	C08a	Non-breast cancer malignancy
Microscopically unconfirmed primary cancer of liver/lung/brain/bone	C08b	
New primary non-breast cancer	C08c	
Death from non-breast cancer	C09	Non-breast-cancer death
Death with no cause recorded in the registry with previous breast surgery	C10a	
Death with no cause recorded in the registry without previous breast surgery	C10b	
Death from cause other than cancer	C11	
Embarkation	D01	No event recorded
No events of any type during study period	E	

[a] Women with these outcomes were excluded from further consideration during the comparison with routinely collected data.

Where two or more outcomes occurred on the same day, the one listed first in the table took priority

(a) Initial algorithm rule

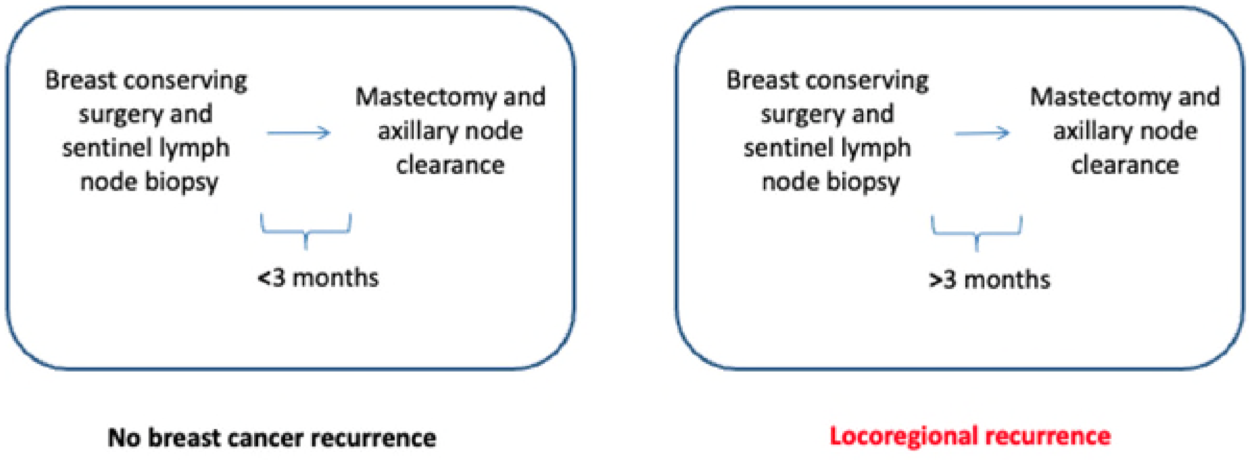

(b) Algorithm rule refined after examination of data from 150 women from the AZURE cohort

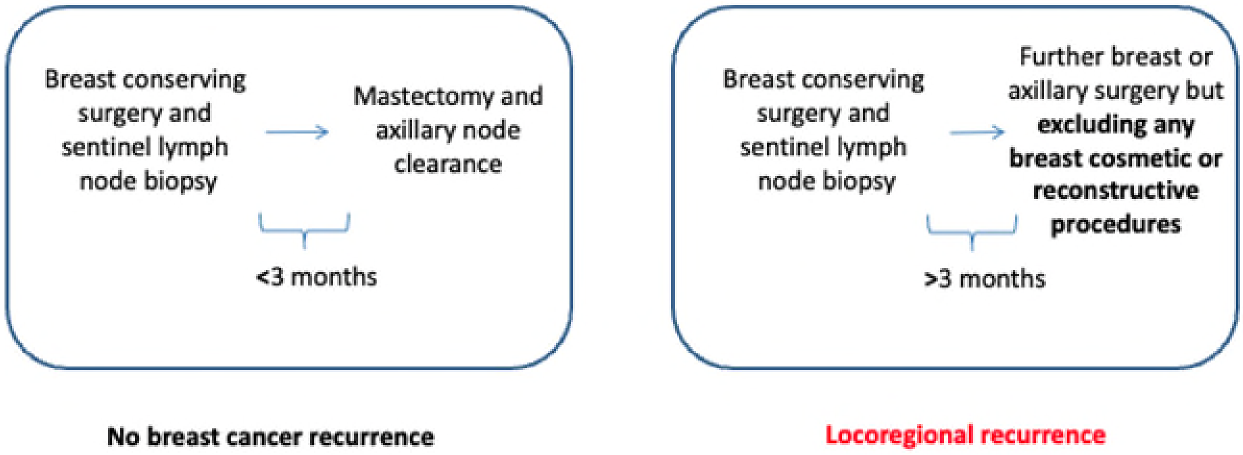

Figure S1. (a) Example of initial coding rule used to help differentiate breast cancer recurrence from standard clinical care pathway based on the time period between certain procedures. (b) Example of refinement of initial coding rule following findings from the training exercise using 150 women from the AZURE trial.

Text S1: Summary of classifications used to derive outcomes for algorithm based on routinely collected data.

The following text describes, for each of the outcomes recorded in the WMCIU database (listed in Table S1), the items in the routinely collected data (RCD) that were taken to indicate that the outcome had occurred. It necessarily refers to other coding schemes (OPCS4, ICD-10, ICD-9, SNOMED, NICIP). The relevant codes from these schemes are listed in Tables S2a-S2g & S3 and references to the manuals for the coding schemes are listed at the end of Table S2g. Additional abbreviations used in the text below are: COSD, Cancer Outcomes and Services Dataset; CWT, Cancer Waiting Times; HES, Hospital Episode Statistics; NMSC, Non-melanoma skin cancer; NEC, not elsewhere classified; RTDS, Radiotherapy Dataset; SACT, Systemic Anti-Cancer Therapy; DID, Diagnostic Imaging Dataset

---

C01 – Death from breast cancer

Breast cancer (ICD-10 code: C50, ICD-9 code: 174) entered in any of the following death certification cause of death fields: 1a, 1b, 1c or 'underlying'. Breast cancer (ICD-10 code: C50, ICD-9 code: 174), 'Breast unspecified' or 'malignant neoplasm' that only appears in cause of death field 2 is not included.

This code is further subdivided based on whether there is evidence that the patient underwent breast surgery (into codes ***C01a – Death from breast cancer, having had breast surgery***; and ***C01b – Death from breast cancer but has never had breast surgery***). For this subdivision, breast surgery is determined if the patient has code '01' (first definitive treatment for a new primary cancer) in the cancer treatment event type variable in COSD/CWT or any of the OPCS4 codes in Supplementary Table S2a from HES.

C03 – Distant metastases

Distant metastases classifications are subdivided into 4 categories:

C03a(i): Distant metastases of liver/lung/bone/brain after 3 months

Sites of secondary cancer defined as

Cancer site	ICD-10 codes
Liver	C78.7
Lung	C78.0, C78.1, C78.2
Bone	C79.5
Brain	C79.3, C79.4

Indicated by one or more of the following occurring at least 3 months after date of original cancer diagnosis in these 4 anatomical sites:

- COSD: Type of recurrence indicates metastatic disease ('05')

- CWT: if there is any information regarding metastases site or the cancer treatment event type is one of the following:

'05' Treatment for a distant recurrence of cancer (metastatic disease)

'07' First treatment for metastatic disease following an unknown primary

'08' Second or subsequent treatment for metastatic disease following an unknown primary

- HES: All women regarded as stage IV from the HES diagnosis codes (ICD-10 codes C77-C79) using criteria listed in Supplementary Table S3.

C03a(ii): Distant metastases in other locations after 3 months

There is a registration of a new secondary malignancy in an anatomical site not covered by C03a that occurs at least 93 days after the date of the original diagnosis.

C03a(iii): Palliative care after 3 months

Rationale: Palliative care is only given to cancer patients with distant metastases, so anyone receiving it after 3 months from diagnosis can be classified as C3a (i.e. confirmed distant metastasis).

Palliative care indicated if any of the following event codes are recorded for the cancer treatment modality (CWT/COSD):

'07' – specialist palliative care (CWT),

'09' – non-specialist palliative care (CWT).

C03b: Distant metastases of liver/lung/bone/brain (unconfirmed) after 3 months

Women who do not have any other invasive cancer recorded, excluding NMSC, (ICD-10 codes: C00-C97, including C50, but excluding C44; ICD-9 codes: 140-209, including 174, but excluding 173) but have one or more of the OPCS4 codes listed in Supplementary Table S2b, recorded in COSD/HES after 12 months of diagnosis, irrespective of the laterality of the procedure. However, with the following exceptions:

**Exception**	**Rationale**
If OPCS4 codes ‘L912’ (Insertion of central venous catheter NEC) or ‘L913’ (Attention to central venous catheter NEC) occur within 400 days (approximately 13 months) of June 2005	These codes are likely to be generated by Trastuzumab treatment, which was introduced around June 2005 and is administered every 3 weeks with a standard treatment duration of 12 months.
If OPCS4 code W085 (Partial excision of bone NEC) is combined with HES diagnosis code M751 (Rotator cuff syndrome) or M77 (Other enthesopathies)	Likely to be a routine removal of bone as part of orthopaedic surgery for musculoskeletal conditions and not a distant recurrence

Women in C03b may either have (1) one or more of the cancers in C08a that was not registered, (2) an occult breast cancer recurrence or (3) no second cancer. The

classification algorithm will be re-run on these women after excluding the 'first event' to see which category they subsequently fall into.

C07 – Cancer in the contralateral breast or the contralateral lymph nodes

This is also referred to as the woman having had bilateral breast cancer. Indicated by one or more of the following:

- COSD: Another, later breast tumour with different laterality

- HES: OPCS4 code for sentinel lymph node biopsy (see exception below), sampling or axillary dissection (see Supplementary Table S2a) **AND** the laterality relating to the event is not the same as recorded for the tumour.

- HES/COSD: OPCS4 code for breast conserving surgery or mastectomy (see Supplementary Table S2a) **AND** the laterality relating to the event is not the same as recorded for the tumour.

However, with the following exception:

**Exception**	**Rationale**
An excision or biopsy of axillary lymph node (i.e. HES OPCS4 code 'T873') is not considered to indicate a contralateral breast cancer.	A biopsy without any further surgery/treatment is likely to be benign.
If a code for bilateral procedure (i.e. HES OPCS4 code 'Z941') is present and accompanied by any of the breast reconstruction OPCS4 codes listed in Supplementary Table S2c, then this is not counted as a contralateral breast cancer.	Breast reconstruction should not indicate a recurrence.
If the term 'prophylactic' surgery (i.e. ICD-10 codes listed in Supplementary Table S2d) from the HES data is present and in the absence of a new diagnosis of invasive breast cancer, then this will not be considered a contralateral breast recurrence.	Risk-reducing surgery (e.g. for genetic predisposition) with a cancer diagnosis, follow-up appointments relating to reconstructive or cosmetic procedures and wound-care related follow-up should not indicate a recurrence.

C04 – Recurrence of cancer in regional lymph nodes

Classifications for recurrences in the regional lymph nodes are subdivided into 2 categories, based on whether laterality is recorded:

C04a: Recurrence of cancer in the ipsilateral regional lymph nodes

Indicated by one or more of the following occurring at least 6 months after date of original cancer diagnosis:

- HES/COSD: OPCS4 code indicating surgery for nodes and metastases: i.e. OPCS4 codes for sentinel lymph node biopsy, sampling or axillary dissection

(see Supplementary Table S2a) **AND** the laterality relating to the event is the same as recorded for the tumour

- COSD/CWT: Treatment event type ‘04’: Treatment for a regional recurrence of cancer

C04b: Recurrence of cancer in the regional lymph nodes with no laterality recorded (assumed to be ipsilateral)

Indicated by one or more of the events listed in category ‘**C04a**’, occurring at least 6 months after date of original cancer diagnosis, but with no laterality recorded in any routinely collected dataset. In this setting it is assumed that the recurrence of cancer is in the ipsilateral regional lymph nodes, in the absence of evidence to the contrary.

However, with the following exception:

Exception	Rationale
If there is a reconstructive surgery code (and not oncological surgery) on the same date, even if accompanied by mastectomy codes ‘B274’, ‘B278’ or ‘B275’. The OPCS4 codes used to define reconstructive surgery are listed in Supplementary Table S2c.	In this situation the mention of nodal involvement was likely attributable to the incident surgery/encounter (and the diagnosis has been duplicated in subsequent encounters) rather than a formal new diagnosis

C05 – Recurrence of cancer in the ipsilateral breast

Indicated by one or more of the following occurring at least 3 months after date of original cancer diagnosis:

- COSD: Another, later breast tumour with same laterality

- COSD/CWT: event type of ‘03’, i.e. Treatment for a local recurrence of a primary cancer.

- HES/COSD: OPCS4 code for breast conserving surgery or mastectomy (see Supplementary Table S2a) **AND** the laterality relating to the event is the same as recorded for the original tumour, **AND** surgery is not within 6 months of the last date of neo-adjuvant chemotherapy.

However, with the following exception:

Exception	Rationale
If there is a reconstructive surgery code (and not oncological surgery) on the ipsilateral side of previous breast surgery, even if accompanied by mastectomy codes ‘B274’, ‘B278’ or ‘B275’. The OPCS4 codes used to define reconstructive surgery are listed in Supplementary Table S2c.	In this situation the mention of nodal involvement was likely attributable to the incident surgery/encounter (and the diagnosis has been duplicated in subsequent encounters) rather than a formal new diagnosis

C06 – Breast cancer recurrence of unknown location

Indicated by one or more of the following:

- CWT: Variable for cancer or symptomatic breast referral patient status indicates that recurrent cancer is suspected or confirmed, i.e. one of the following is present:

  ‘15’ Suspected recurrent cancer

  ‘16’ Diagnosis of recurrent cancer confirmed - first NHS funded treatment not yet planned

  ‘17’ Diagnosis of recurrent cancer confirmed - NHS funded first treatment planned

  ‘18’ Diagnosis of recurrent cancer confirmed - no NHS funded treatment planned

  ‘19’ Diagnosis of recurrent cancer confirmed – subsequent NHS funded treatment not yet planned

  ‘20’ Diagnosis of recurrent cancer confirmed - subsequent NHS funded treatment planned

- COSD/CWT: Variable for cancer treatment event type has one of the following values:

  '06' Treatment for multiple recurrence of cancer (local/regional/distant)

  '09' 2nd or subsequent treatment for relapse of primary cancer

  '10' 2nd or subsequent treatment for progression of primary cancer

  '99'/'U' Unknown recurrence treatment

- COSD: recurrence flag = 'Y'

- COSD/CWT/RTDS: Initiation of radiotherapy ≥1 year after diagnosis (see Supplementary Table S2e for codes used to identify radiotherapy)

- COSD/HES/CWT/SACT: Initiation of chemotherapy ≥ 1 year after diagnosis (see Supplementary Table S2e for codes used to identify chemotherapy)

- COSD/CWT/SACT: Initiation of hormone therapy at any time after diagnosis following an absence of hormone therapy of ≥ 1 year (see Supplementary Table S2e for codes used to identify hormone therapy)

- COSD: Two or more of the following types of scan, within a 1 year time period, at least one year following diagnosis of breast cancer: ‘CT scan with contrast’, ‘CT scan with unspecified contrast’, ‘MRI scan with contrast’, ‘MRI scan with unspecified contrast’, ‘PET scan’, ‘Radio-isotope bone scan’.

- DID: Two or more of the types of scan listed in Supplementary Table S2f, within a 1 year time period, at least one year following diagnosis of breast cancer, OR one or more of the codes listed in Supplementary Table S2g after 1 year of diagnosis.

However, with the following exceptions:

Exception	Rationale
If there is a 21 day repeating cycle of treatment **AND** the first ‘21 day event’ starts within 400 days (approx. 13m) of first surgery/diagnosis or June 2005,<br><br>**AND** the total of ’21 day events’ ≥ 13<br><br>**AND** is within a 450 day period from first ’21 day event’.	This treatment regimen is likely to be Trastuzumab. It was only until after June, 2005, Trastuzumab for HER2+ disease, was given in accordance with standard protocols.
The first SACT event is within 183 days (6m) of first surgery/diagnosis<br><br>**AND** there are ≥ 5 SACT events separated by <31 days in the first year since the first SACT event<br><br>**AND** ≥ 3 SACT events separated by 2-4 months in the second year since first SACT event.	This treatment pattern indicates possible zoledronic acid treatment but was stopped early.
One or two SACT or HES chemotherapy events occurring in the same week without another chemotherapy event in the preceding 5 months or subsequent 5 months	Likely artefactual
If a **C06** code is indicated along with the occurrence of any other invasive cancer (ICD-10 codes: C00-C97, including C50, but excluding C44; ICD-9 codes: 140-209, including 174, but excluding 173), then the **C08** code should take precedent.	

C08 – New primary cancer after 3 months

A new primary cancer is defined as the occurrence of any other invasive cancer (ICD-10 codes: C00-C97, including C50, but excluding C44; ICD-9 codes: 140-209, including 174, but excluding 173) as recorded in COSD.

New primary cancers are subdivided into three groups:

C08a: New microscopically confirmed primary cancer of liver/lung/bone/brain after 3 months

Site of new cancer defined as

Cancer site	ICD-9 code	ICD-10 code
Liver	155	C22
Lung	162	C33-34
Bone	170	C40-41
Brain	191-192	C70-72

AND the cancer is considered microscopically confirmed if one or more of the following codes are recorded within ‘basis of diagnosis’:

- specific tumour markers
- cytology
- histology of metastasis
- histology of primary

C08b: New microscopically unconfirmed primary cancer of liver/lung/bone/brain after 3 months

Site of new cancer is defined as in **C08a**.

AND the cancer is considered microscopically unconfirmed if one or more of the following codes are recorded within ‘basis of diagnosis’:

- death certificate
- clinical
- clinical investigation
- x not known

C08c: New primary cancer in other locations after 3 months

Women falling into category **C08** who have invasive cancers other than those listed in **C08a** irrespective of the ‘basis of diagnosis’.

C09 – Death from cancer other than breast cancer

Any malignant cancer code (ICD-10 codes: C00-C97, ICD-9 codes: 140-209) excluding breast cancer (ICD-10 code: C50, ICD-9 code: 174) entered in any cause of death field 1a, 1b, 1c or ‘underlying’. Codes in cause of death field 2 are not taken into account.

C10 – Death where no cause has been attributed

When there is no information entered in any cause of death field 1a, 1b, 1c, 2 or ‘underlying’.

C11 – Death from cause other than cancer

No mention of cancer (ICD-10 codes: C00-C97, ICD-9 codes: 140-209) entered in any cause of death field 1a, 1b, 1c or ‘underlying’. Codes in cause of death field 2 are not taken into account.

Tables S2a-g: Summaries of code groupings used to derive outcomes for algorithm based on routinely collected data.

Table S2a: OPSC4[1] codes used to identify types of breast cancer surgery

Type of breast surgery	OPCS4 code	OPCS4 code description
Breast conserving surgery	B281	Quadrantectomy of breast
	B282	Partial excision of breast NEC
	B283	Excision of lesion of breast NEC
	B285	Wire guided partial excision of breast
	B286	Excision of accessory breast tissue
	B287	Wire guided excision of lesion of breast
	B288	Other specified other excision of breast
	B289	Unspecified other excision of breast
	B34	Operations on duct of breast
	B341	Subareolar excision of mammary duct
	B342	Excision of mammary duct NEC
	B343	Excision of lesion of mammary duct
	B344	Microdochotomy
	B345	Exploration of mammary duct NEC
	B348	Other specified operations on duct of breast
	B349	Unspecified operations on duct of breast
	B35	Operations on nipple
	B351	Transposition of nipple
	B352	Excision of nipple
	B353	Extirpation of lesion of nipple
	B354	Plastic operations on nipple
	B355	Biopsy of lesion of nipple
	B356	Eversion of nipple
	B358	Other specified operations on nipple
	B359	Unspecified operations on nipple
Mastectomy	B27	Total excision of breast
	B271	Total mastectomy and excision of both pectoral muscles and part of chest wall
	B272	Total mastectomy and excision of both pectoral muscles NEC
	B273	Total mastectomy and excision of pectoralis minor muscle
	B274	Total mastectomy NEC
	B275	Subcutaneous mastectomy
	B276	Skin sparing mastectomy
	B278	Other specified total excision of breast
	B279	Unspecified total excision of breast

(continues on next page)

(Table S2a cont.)

SLNB	O142	Sentinel lymph node
	T87	Excision or biopsy of lymph node
	T873	Excision or biopsy of axillary lymph node
	T878	Other specified excision or biopsy of lymph node
	T879	Unspecified excision or biopsy of lymph node
	T91	Operations on sentinel lymph node
	T911	Biopsy of sentinel lymph node NEC
	T918	Other specified operations on sentinel lymph node
	T919	Unspecified operations on sentinel lymph node
	T928	Other specified other operations on lymphatic tissue
	Z613	Axillary lymph node
Sampling	T86	Sampling of lymph nodes
	T862	Sampling of axillary lymph nodes
	T869	Unspecified sampling of lymph nodes
Axillary Dissection	Z613	Axillary lymph node
	T852	Block dissection of axillary lymph nodes
	T859	Unspecified block dissection of lymph nodes

Table S2b: OPSC4[1] codes for procedures that are likely a result of distant metastases of liver/lung/bone/brain

Operation site or type	OPCS4 code	OPCS4 code description
Liver	J023	Resection of segment of liver
	J024	Wedge excision of liver
	J027	Extended left hemihepatectomy
	J029	Unspecified partial excision of liver
	J031	Excision of lesion of liver NEC
	J032	Destruction of lesion of liver NEC
	J124	Percutaneous radiofrequency ablation of lesion of liver
Lung	T013	Excision of lesion of chest wall
	T018	Other specified partial excision of chest wall
	T019	Unspecified partial excision of chest wall
	T078	Other specified open excision of pleura
	T079	Unspecified open excision of pleura
	T094	Chemical open pleurodesis
	T095	Open pleurodesis NEC
	T101	Endoscopic extirpation of lesion of pleura
	T102	Endoscopic pleurodesis using talc
	T103	Endoscopic pleurodesis NEC
	T121	Drainage of lesion of pleura NEC
	T122	Drainage of pleural cavity NEC
	T124	Insertion of tube drain into pleural cavity
	T131	Insufflation of talc into pleural cavity NEC
	E541	Total pneumonectomy
	E542	Bilobectomy of lung
	E543	Lobectomy of lung
	E544	Excision of segment of lung
	E545	Partial lobectomy of lung NEC
	E548	Other specified excision of lung
	E552	Open excision of lesion of lung
	E558	Other specified open extirpation of lesion of lung
Bone	W085	Partial excision of bone NEC
	W088	Other specified other excision of bone
	W089	Unspecified other excision of bone
	W091	Excision of lesion of bone NEC
	W093	Curettage of lesion of bone NEC
	W096	Curettage of tumour of bone NEC
	W097	Excision of tumour of bone
	W098	Other specified extirpation of lesion of bone
	W099	Unspecified extirpation of lesion of bone
	V257	Primary anterior corpectomy of lumbar spine and reconstruction HFQ
	V431	Excision of lesion of cervical vertebra
	V432	Excision of lesion of thoracic vertebra

(continues on next page)

(Table S2b cont.)

Brain	A021	Excision of lesion of tissue of frontal lobe of brain
	A022	Excision of lesion of tissue of temporal lobe of brain
	A023	Excision of lesion of tissue of parietal lobe of brain
	A024	Excision of lesion of tissue of occipital lobe of brain
	A025	Excision of lesion of tissue of cerebellum
	A026	Excision of lesion of tissue of brain stem
	A028	Other specified excision of lesion of tissue of brain
	A029	Unspecified excision of lesion of tissue of brain
	A178	Other specified therapeutic endoscopic operations on ventricle of brain
	V051	Extirpation of lesion of cranium
Other	B343	Excision of lesion of mammary duct
	B401	Interstitial laser destruction of lesion of breast
	Q241	Salpingo ophorectomy NEC
	S049	Unspecified other excision of skin
	T882	Drainage of lesion of axillary lymph node
	L912	Insertion of central venous catheter NEC
	L913	Attention to central venous catheter NEC
	L915	Insertion of tunnelled venous catheter
	L943	Percutaneous transluminal insertion of subcutaneous port

Table S2c: OPSC4[1] codes used to identify types of breast reconstructive surgery

OPCS4 code	OPCS4 code description
B291	Reconstruction of breast using myocutaneous flap of latissimus dorsi muscle
B293	Reconstruction of breast using flap of skin of abdomen NEC
B295	Revision of reconstruction of breast
B298	Other specified reconstruction of breast
B299	Unspecified reconstruction of breast
B301	Insertion of prosthesis for breast
B302	Revision of prosthesis for breast
B303	Removal of prosthesis for breast
B304	Renewal of prosthesis for breast
B308	Other specified prosthesis for breast
B309	Unspecified prosthesis for breast
B311	Reduction mammoplasty
B312	Augmentation mammoplasty
B313	Mastopexy
B314	Revision of mammoplasty
B318	Other specified other plastic operations on breast
B319	Unspecified other plastic operations on breast
B354	Plastic operations on nipple
B358	Other specified operations on nipple
B374	Capsulectomy of breast
B375	Lipofilling of breast
B378	Other specified other operations on breast
B379	Unspecified other operations on breast
B381	Reconstruction of breast using free superior gluteal artery perforator flap
B382	Reconstruction of breast using free inferior gluteal artery perforator flap
B388	Other specified reconstruction of breast using flap of skin of buttock
B389	Unspecified reconstruction of breast using flap of skin of buttock
B391	Reconstruction of breast using free transverse rectus abdominis myocutaneous flap
B392	Reconstruction of breast using pedicled transverse rectus abdominis myocutaneous flap
B393	Reconstruction of breast using free deep inferior epigastric perforator flap
B394	Reconstruction of breast using pedicled omental flap
B395	Reconstruction of breast using free omental flap
B398	Other specified reconstruction of breast using abdominal flap
B399	Unspecified reconstruction of breast using abdominal flap

Table S2d: ICD-10 codes[2] used to identify types of prophylactic surgery

ICD-10 code	ICD-10 code description
Z400	Prophylactic surgery for risk-factors related to malignant neoplasms
Z421	Follow-up care involving plastic surgery of breast
Z422	Follow-up care involving plastic surgery of other parts of trunk
Z429	Follow-up care involving plastic surgery, unspecified
Z443	Fitting and adjustment of external breast prosthesis
Z418	Other procedures for purposes other than remedying health state
Z419	Procedure for purposes other than remedying health state, unspecified
Z480	Attention to surgical dressings and sutures
Z488	Other specified surgical follow-up care
Z489	Surgical follow-up care, unspecified

Table S2e: Codes used to identify types of breast cancer treatment.

Treatment type	Dataset and code type	Code	Code description
Chemo-therapy	COSD/HES - OPCS4 codes	X70	Procurement of drugs for chemotherapy for neoplasm in Bands 1-5
		X71	Procurement of drugs for chemotherapy for neoplasm in Bands 6-10
		X72	Delivery of chemotherapy for neoplasm
		X73	Delivery of oral chemotherapy for neoplasm
		X74	Other chemotherapy drugs
		X28	Intermittent infusion of therapeutic substance
		X29	Continuous Infusion of therapeutic substance
		X35	Other intravenous injection
		X37	Intramuscular injection
		X38	Subcutaneous injection
		X89	High cost immunosuppressant drugs
	HES - ICD-10 diagnosis code	Z511	Chemotherapy session for neoplasm
	CWT/COSD-Cancer treatment modality variable	02	Anti-cancer drug regimen (Cytotoxic Chemotherapy)
		14	Anti-cancer drug regimen (other)
Radio-therapy	COSD/HES - OPCS4 codes	X65	Radiotherapy delivery
	CWT/COSD-Cancer treatment modality variable	05	Teletherapy (Beam Radiation excluding Proton Therapy)
		06	Brachytherapy
		22	Radiosurgery
Hormone therapy	CWT/COSD-Cancer treatment modality variable	03	Anti-cancer drug regimen (Hormone Therapy)
Immuno-therapy	CWT/COSD-Cancer treatment modality variable	15	Anti-cancer drug regimen (Immunotherapy)

Table S2f: DID procedure codes (SNOMED[3] and NICIP[4]) within the first year after diagnosis.

Procedure (SNOMED description)	SNOMED code (SCT-ID)	NICIP code
Computed tomography of abdomen	169070004	CABDO
Computed tomography and biopsy of abdomen	419940006	CABDOB
Computed tomography of abdomen and pelvis	419394006	CABPE
Computed tomography of chest and abdomen	418891003	CCABD
Computed tomography of chest, abdomen and pelvis	418023006	CCHAP
Computed tomography of chest	169069000	CCHES
Computed tomography of cervical spine	241578008	CCSPN
Computed tomography of liver	241549007	CLIVE
Computed tomography and biopsy of liver	418749009	CLIVEB
Computed tomography of lumbar spine	241580002	CLSPN
Computed tomography of mediastinum	241543008	CMEDM
Computed tomography of pituitary fossa	241519005	CPITF
Computed tomography of posterior fossa	241518002	CPOSF
Computed tomography of sacral spine	241581003	CSACM
Computed tomography of thoracic spine	241579000	CTSPN
Virtual computed tomography bronchoscopy	418419008	CVBRY
Magnetic resonance imaging of abdomen	241621009	MABDO
Ablation of lesion of liver using computed tomography guidance	432226009	CLMAAA
Radiofrequency ablation of lesion of liver using computed tomography guidance	431475009	CLMAFA
Laser ablation of lesion of liver using computed tomography guidance	433866007	CLMASA
Computed tomography of neck and thorax	430448007	CNECH
Computed tomography of neck and thorax with contrast	429927002	CNECHC
Computed tomography of neck with contrast	431326009	CNECKC
Computed tomography of sacral spine with contrast	430452007	CSACMC
Computed tomography of sternum	429874005	CSTRM
Magnetic resonance imaging of chest with contrast	432815006	MCHESC
Magnetic resonance imaging of liver with contrast	431839003	MLIVEC

Table S2g: DID procedure codes (SNOMED[3] and NICIP[4]) at least one year following diagnosis.

Procedure (SNOMED description)	SNOMED code (SCT-ID)	NICIP code
Computed tomography of breast for radiotherapy planning	429866009	CRTMAR
Computed tomography of head for radiotherapy planning	429867000	CRTSKR
Computed tomography of spine for radiotherapy planning	430438005	CRTSPR
Magnetic resonance imaging of chest for radiotherapy planning	431766006	MRTCHR
Magnetic resonance imaging of breast for radiotherapy planning	430243000	MRTMAR
Radiofrequency ablation using ultrasound guidance	433058002	URAFGA
Wire guided localisation of lesion using ultrasonography guidance	915381000000106	UWLLE
Wire guided localisation of breast lesion using magnetic resonance imaging guidance	911831000000104	MWLBL
Wire guided localisation of breast lesion using magnetic resonance imaging guidance	911831000000104	MWLBR
Wire guided localisation of breast lesion using magnetic resonance imaging guidance	911831000000104	MWLBB
Biopsy of femur using fluoroscopic guidance	431455000	FTHIBB
Biopsy of lung using computed tomography guidance	429932001	CLUNGB
Biopsy of brain using computed tomography guidance	432666003	CSKUHB

References

1. **OPCS4:** NHS Digital, *National Clinical Coding Standards OPCS-4*. Accurate data for quality information, 2022. Version: [9.1].

2. **ICD:** World Health Organization. *International Statistical Classification of Diseases and Related Health Problems*
10th Revision. 2019 [cited 2024 23/07/2024]; Available from: https://icd.who.int/browse10/2019/en.
9th Revision. 1975. World Health Organisation: Geneva

3. **SNOMED:** International Health Terminology Standards Development Organisation. *SNOMED Clinical Terms*. 2007 [cited 2024 23/07/2024]; Available from: https://www.snomed.org/.

4. **NICIP:** *National Interim Clinical Imaging Procedure (NICIP) Code Set. NICIP code set* 2016 [cited 2024 23/07/2024]; Available from: https://digital.nhs.uk/services/terminology-and-classifications/national-interim-clinical-imaging-procedure-nicip-code-set.

Table S3: For women registered as stage VI (unknown stage), ICD-10 codes C77-C79 from HES data (within 3 months of diagnosis of breast cancer) were used to determine cancer stage.

ICD-10 code	Sub-classification	Cancer stage to be allocated
C77 Secondary and unspecified malignant neoplasm of lymph nodes	C77.0 Lymph nodes of head, face and neck Supraclavicular lymph nodes	II
	C77.1 Intrathoracic lymph nodes	II
	C77.2 Intra-abdominal lymph nodes	VI (Not staged)
	C77.3 Axillary and upper limb lymph nodes Pectoral lymph nodes	II
	C77.4 Inguinal and lower limb lymph nodes	VI (Not staged)
	C77.5 Intrapelvic lymph nodes	VI (Not staged)
	C77.8 Lymph nodes of multiple regions	II
	C77.9 Lymph node, unspecified	II
C78 Secondary malignant neoplasm of respiratory and digestive organs	C78.0 Secondary malignant neoplasm of lung	IV
	C78.1 Secondary malignant neoplasm of mediastinum	IV
	C78.2 Secondary malignant neoplasm of pleura Malignant pleural effusion NOS	IV
	C78.3 Secondary malignant neoplasm of other and unspecified respiratory organs	IV
	C78.4 Secondary malignant neoplasm of small intestine	VI (Not staged)
	C78.5 Secondary malignant neoplasm of large intestine and rectum	VI (Not staged)
	C78.6 Secondary malignant neoplasm of retroperitoneum and peritoneum, Malignant ascites NOS	IV
	C78.7 Secondary malignant neoplasm of liver and intrahepatic bile duct	IV
	C78.8 Secondary malignant neoplasm of other and unspecified digestive organs	IV
C79 Secondary malignant neoplasm of other and unspecified sites	C79.0 Secondary malignant neoplasm of kidney and renal pelvis	VI (Not staged)
	C79.1 Secondary malignant neoplasm of bladder and other and unspecified urinary organs	VI (Not staged)
	C79.2 Secondary malignant neoplasm of skin	IV
	C79.3 Secondary malignant neoplasm of brain and cerebral meninges	IV
	C79.4 Secondary malignant neoplasm of other and unspecified parts of nervous system	IV
	C79.5 Secondary malignant neoplasm of bone and bone marrow	IV
	C79.6 Secondary malignant neoplasm of ovary	VI (Not staged)
	C79.7 Secondary malignant neoplasm of adrenal gland	VI (Not staged)
	C79.8 Secondary malignant neoplasm of other specified sites	IV
	C79.9 Secondary malignant neoplasm, unspecified site, Carcinomatosis (secondary), Disseminated (secondary): cancer NOS, malignancy NOS, Generalized (secondary): cancer NOS, Malignancy NOS, Multiple secondary cancer NOS, Sarcomatosis (secondary) NOS	IV

Table S4: Summary of the clinical events used for each of the different analysis outcomes considered. Clinical events grouped according to Table S1.

Analysis outcome	Events considered for analyses		
	Outcome events	Censored events[a]	Ignored events
**Distant recurrence**	Distant metastases<br>Death from breast cancer	Non-breast-cancer malignancy<br>Non-breast-cancer death	Locoregional recurrence<br>Recurrence, type unknown[b]<br>Contralateral breast cancer
**Locoregional recurrence**	Locoregional recurrence	Distant metastases<br>Contralateral breast cancer<br>Non-breast-cancer malignancy<br>Any death	Recurrence, type unknown[b]
**Contralateral breast cancer**	Contralateral breast cancer	Distant metastases<br>Locoregional recurrence<br>Non-breast-cancer malignancy<br>Any death	Recurrence, type unknown[b]
**Any recurrence**	Locoregional recurrence<br>Distant metastases<br>Recurrence, type unknown[b]<br>Contralateral breast cancer<br>Death from breast cancer	Non-breast-cancer malignancy<br>Non-breast-cancer death	-
**Breast cancer mortality**	Death from breast cancer	Non-breast-cancer death	Locoregional recurrence<br>Recurrence, type unknown[b]<br>Contralateral breast cancer<br>Distant metastases<br>Non-breast-cancer malignancy
**All-cause mortality**	Death from breast cancer<br>Non-breast-cancer death	-	Locoregional recurrence<br>Recurrence, type unknown[b]<br>Contralateral breast cancer<br>Distant metastases<br>Non-breast-cancer malignancy
**Any recurrence: Post-validation adjustment**	***All time periods:***<br>Distant metastases<br>Death from breast cancer<br>***After first year from diagnosis:***<br>Locoregional recurrence<br>Recurrence, type unknown[b]<br>Contralateral breast cancer	Non-breast-cancer malignancy<br>Non-breast-cancer death	***Within first year after diagnosis:***<br>Locoregional recurrence<br>Recurrence, type unknown[b]<br>Contralateral breast cancer

[a] Women were removed from the analysis when a censored event occurred
[b] Event code not present in AZURE trial data

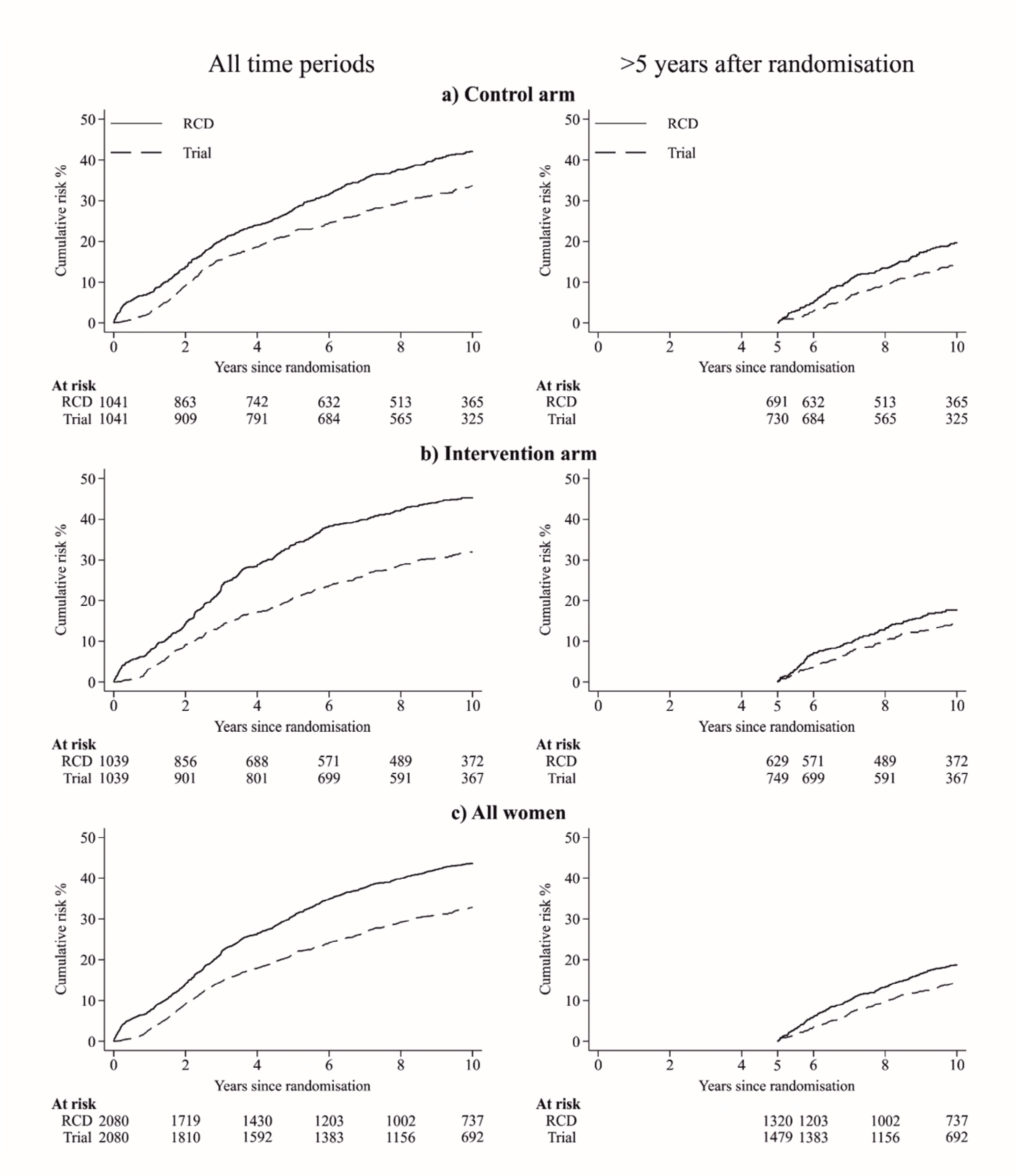

Figure S2. External validation exercise: Cumulative risk of distant recurrence in AZURE trial (dashed lines) and in the routinely collected data (RCD) (solid lines). Analyses are by separate randomisation arm (a and b), and for all 2080 women included in the external validation group (c). The outcome of distant recurrence is defined in Table S4. Plotted values at 1-, 3-, 5- and 10-years (and 95% confidence intervals) are in Table S5.

Table S5. External validation exercise: Number of events and cumulative risks of distant recurrence by time since randomisation and AZURE trial arm, for routinely collected data (RCD) and AZURE trial data. Calculated for all 2080 women in the external validation cohort.

Trial Arm	Dataset	Time since randomisation (years)	All time periods: No. women at risk[a]	All time periods: No. events[b]	All time periods: Cumulative risk[c] % (95% CI)	>5 years after randomisation: No. women at risk[a]	>5 years after randomisation: No. events[b]	>5 years after randomisation: Cumulative risk[c] % (95% CI)
**Control**	RCD	0-1	1041	76	7.3 (5.9, 9.1)	-	-	-
		1-3	952	131	20.3 (18.0, 22.9)	-	-	-
		3-5	789	73	27.8 (25.1, 30.7)	-	-	-
		5-10	691	121	42.6 (39.5, 45.9)	691	121	20.6 (17.5, 24.1)
	Trial	0-1	1041	25	2.4 (1.6, 3.6)	-	-	-
		1-3	995	131	15.4 (13.3, 17.8)	-	-	-
		3-5	840	65	22.2 (19.7, 24.9)	-	-	-
		5-10	730	93	34.8 (31.7, 38.1)	730	93	16.2 (13.4, 19.5)
**Intervention**	RCD	0-1	1039	77	7.5 (6.0, 9.3)	-	-	-
		1-3	941	159	23.3 (20.8, 26.1)	-	-	-
		3-5	757	99	33.6 (30.7, 36.6)	-	-	-
		5-10	629	103	46.0 (42.8, 49.2)	629	103	18.7 (15.7, 22.2)
	Trial	0-1	1039	33	3.2 (2.3, 4.5)	-	-	-
		1-3	981	106	13.8 (11.8, 16.1)	-	-	-
		3-5	847	65	20.6 (18.2, 23.2)	-	-	-
		5-10	749	97	33.3 (30.2, 36.5)	749	97	16.0 (13.3, 19.2)
**Total**	RCD	0-1	2080	153	7.4 (6.4, 8.6)	-	-	-
		1-3	1893	290	21.8 (20.1, 23.7)	-	-	-
		3-5	1546	172	30.7 (28.7, 32.7)	-	-	-
		5-10	1320	224	44.3 (42.0, 46.6)	1320	224	19.6 (17.5, 22.1)
	Trial	0-1	2080	58	2.8 (2.2, 3.6)	-	-	-
		1-3	1976	237	14.6 (13.2, 16.2)	-	-	-
		3-5	1687	130	21.4 (19.6, 23.2)	-	-	-
		5-10	1479	190	34.0 (31.8, 36.3)	1479	190	16.1 (14.1, 18.3)

[a] Number of women at risk at start of time category
[b] Number of events during time category
[c] Cumulative risk at end of time category

Table S6. External validation exercise: Agreement of routinely collected data (RCD) and AZURE trial data for distant recurrence. Calculations performed for all 2080 women in external validation cohort. The outcome of distant recurrence is defined in Table S4.

Distant recurrence[a]	Analysis period and trial arm					
	All time periods			>5 years after randomisation[b]		
	Control (N=1041)	Intervention (N=1039)	Total (N=2080)	Control (N=1041)	Intervention (N=1039)	Total (N=2080)
			No.			
Event in both datasets	294	289	583	71	65	136
Event only in trial data	23	15	38	14	10	24
Event only in RCD	110	149	259	32	26	58
No event in either dataset	614	586	1200	537	509	1046
**Censored before analysis period**						
Only in trial data	0	0	0	37	19	56
Only in RCD	0	0	0	76	139	215
In both datasets	0	0	0	274	271	545
**Time difference when event present in both datasets**			No. (%)			
<6 months	199 (67.7%)	181 (62.6%)	380 (65.2%)	58 (81.7%)	55 (84.6%)	113 (83.1%)
6-12 months	37 (12.6%)	39 (13.5%)	76 (13.0%)	9 (12.7%)	5 (7.7%)	14 (10.3%)
>1 year	58 (19.7%)	69 (23.9%)	127 (21.8%)	4 (5.6%)	5 (7.7%)	9 (6.6%)
**Performance measures[c]**			% (95% CI)			
All time periods						
Sensitivity	92.7 (89.9, 95.6)	95.1 (92.6, 97.5)	93.9 (92.0, 95.8)	83.5 (75.6, 91.4)	86.7 (79.0, 94.4)	85.0 (79.5, 90.5)
Specificity	84.8 (82.2, 87.4)	79.7 (76.8, 82.6)	82.2 (80.3, 84.2)	94.4 (92.5, 96.3)	95.1 (93.3, 97.0)	94.7 (93.4, 96.1)
PPV	72.8 (68.4, 77.1)	66.0 (61.5, 70.4)	69.2 (66.1, 72.4)	68.9 (60.0, 77.9)	71.4 (62.1, 80.7)	70.1 (63.7, 76.5)
NPV	96.4 (94.9, 97.8)	97.5 (96.3, 98.8)	96.9 (96.0, 97.9)	97.5 (96.1, 98.8)	98.1 (96.9, 99.3)	97.8 (96.9, 98.6)
Within 6 months of trial data						
Sensitivity	62.8 (57.5, 68.1)	59.5 (54.0, 65.1)	61.2 (57.4, 65.0)	68.2 (58.3, 78.1)	73.3 (63.3, 83.3)	70.6 (63.6, 77.7)
Specificity	84.8 (82.2, 87.4)	79.7 (76.8, 82.6)	82.2 (80.3, 84.2)	94.4 (92.5, 96.3)	95.1 (93.3, 97.0)	94.7 (93.4, 96.1)
PPV	64.4 (59.1, 69.7)	54.8 (49.5, 60.2)	59.5 (55.7, 63.3)	64.4 (54.6, 74.3)	67.9 (57.7, 78.1)	66.1 (59.0, 73.2)
NPV	83.9 (81.2, 86.5)	82.7 (79.9, 85.4)	83.3 (81.3, 85.2)	95.2 (93.5, 97.0)	96.2 (94.6, 97.8)	95.7 (94.5, 96.9)

PPV, Positive predictive value; NPV, Negative predictive value

[a] Women are censored at non-breast cancer malignancies or non-breast-cancer deaths

[b] Analysis period starts at 5 years after randomisation

[c] Performance measures defined in footnote of Table 3

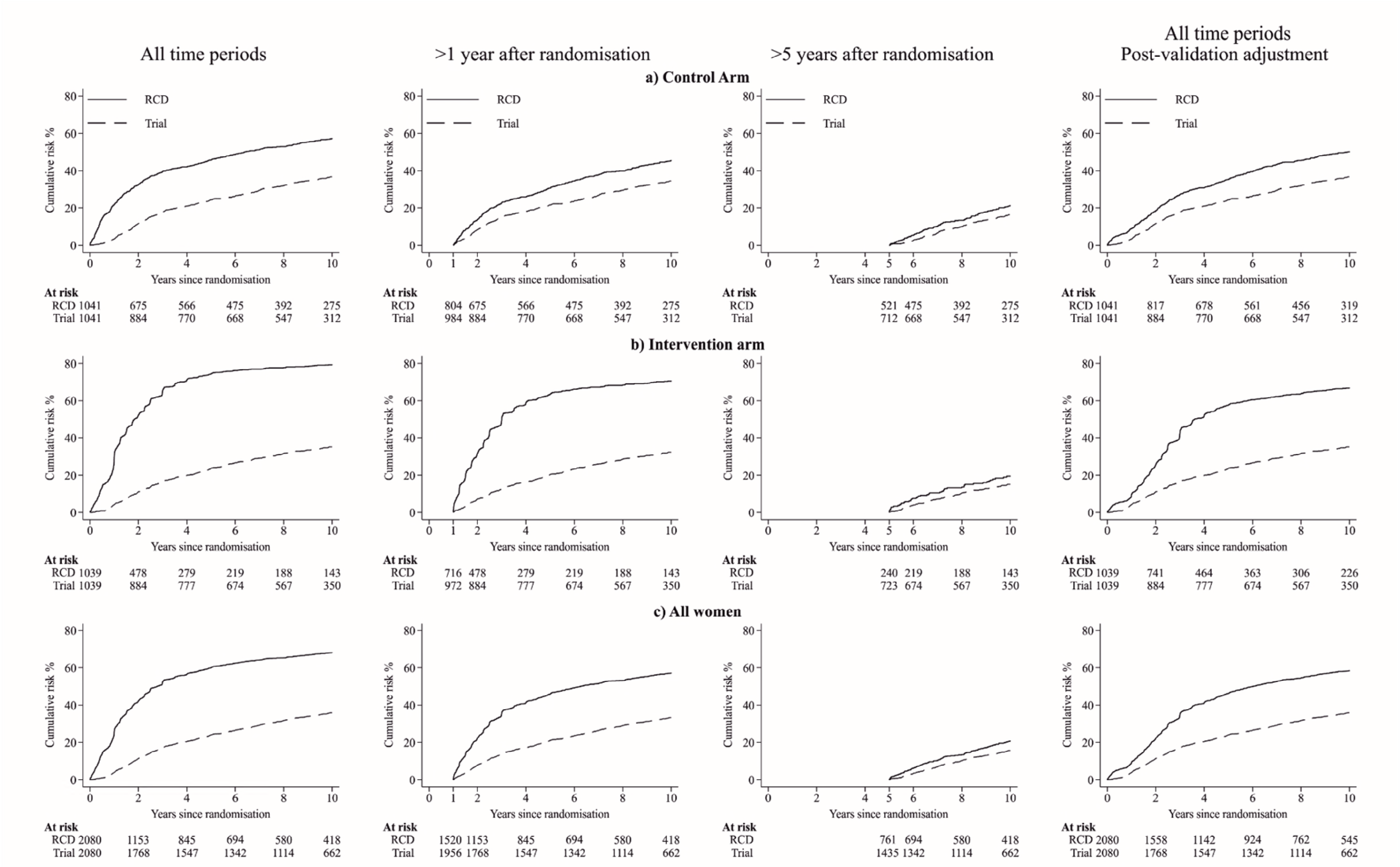

Figure S3. External validation exercise: Cumulative risk of any recurrence in AZURE trial (dashed lines) and in routinely collected data (RCD) (solid lines). Analyses are by separate randomisation arm (a and b), and for all 2080 women included in external validation group (c). The outcome of any recurrence is defined in Table S4. Plotted values at 1-, 3-, 5- and 10-years (and 95% confidence intervals) are in Table S7.

Table S7. External validation exercise: Number of events and cumulative risks of any recurrence by time since randomisation and AZURE trial arm, for routinely collected data (RCD) and AZURE trial data. Calculated for all 2080 women in the external validation cohort.

Trial Arm and Dataset	Time since random-isation (years)	All time periods			>1 year after randomisation			>5 years after randomisation			All time periods with post-validation adjustment		
		No. women at risk[a]	No. events[b]	Cumulative risk[c] % (95% CI)	No. women at risk[a]	No. events[b]	Cumulative risk[c] % (95% CI)	No. women at risk[a]	No. events[b]	Cumulative risk[c] % (95% CI)	No. women at risk[a]	No. events[b]	Cumulative risk[c] % (95% CI)
**Control**													
RCD	0-1	1041	225	21.7 (19.3, 24.4)	-	-	-	-	-	-	1041	93	9.0 (7.4, 10.9)
	1-3	804	180	39.5 (36.6, 42.6)	804	180	22.8 (20.0, 25.8)	-	-	-	935	184	27.2 (24.5, 30.0)
	3-5	598	60	45.7 (42.7, 48.8)	598	60	30.6 (27.5, 34.0)	-	-	-	723	79	35.3 (32.4, 38.3)
	5-10	521	98	57.6 (54.5, 60.8)	521	98	45.8 (42.2, 49.6)	521	98	21.9 (18.4, 26.1)	621	128	50.8 (47.6, 54.1)
Trial	0-1	1041	36	3.5 (2.5, 4.8)	-	-	-	-	-	-	1041	36	3.5 (2.5, 4.8)
	1-3	984	143	17.7 (15.5, 20.2)	984	143	14.7 (12.6, 17.1)	-	-	-	984	143	17.7 (15.5, 20.2)
	3-5	817	64	24.3 (21.8, 27.1)	817	64	21.6 (19.1, 24.3)	-	-	-	817	64	24.3 (21.8, 27.1)
	5-10	712	100	37.8 (34.6, 41.1)	712	100	35.5 (32.4, 38.9)	712	100	17.8 (14.9, 21.2)	712	100	37.8 (34.6, 41.1)
**Intervention**													
RCD	0-1	1039	308	29.9 (27.2, 32.8)	-	-	-	-	-	-	1039	100	9.7 (8.1, 11.7)
	1-3	716	353	64.9 (61.9, 67.8)	716	353	49.9 (46.3, 53.7)	-	-	-	918	329	42.5 (39.5, 45.6)
	3-5	345	90	74.2 (71.5, 76.9)	345	90	63.3 (59.7, 66.9)	-	-	-	564	144	57.5 (54.4, 60.6)
	5-10	240	44	79.6 (76.9, 82.1)	240	44	70.8 (67.3, 74.3)	240	44	20.6 (15.8, 26.7)	399	80	67.1 (64.1, 70.1)
Trial	0-1	1039	43	4.2 (3.1, 5.6)	-	-	-	-	-	-	1039	43	4.2 (3.1, 5.6)
	1-3	972	122	16.4 (14.2, 18.8)	972	122	12.7 (10.8, 15.0)	-	-	-	972	122	16.4 (14.2, 18.8)
	3-5	825	70	23.6 (21.1, 26.4)	825	70	20.3 (17.8, 23.0)	-	-	-	825	70	23.6 (21.1, 26.4)
	5-10	723	98	36.4 (33.3, 39.7)	723	98	33.6 (30.5, 36.9)	723	98	16.7 (13.9, 20.0)	723	98	36.4 (33.3, 39.7)
**Total**													
RCD	0-1	2080	533	25.8 (24.0, 27.7)	-	-	-	-	-	-	2080	193	9.4 (8.2, 10.7)
	1-3	1520	533	52.2 (50.0, 54.4)	1520	533	35.6 (33.2, 38.1)	-	-	-	1853	513	34.8 (32.8, 36.9)
	3-5	943	150	59.9 (57.8, 62.1)	943	150	46.0 (43.5, 48.6)	-	-	-	1287	223	46.3 (44.1, 48.5)
	5-10	761	142	68.5 (66.4, 70.6)	761	142	57.6 (55.0, 60.2)	761	142	21.5 (18.6, 24.8)	1020	208	58.9 (56.7, 61.2)
Trial	0-1	2080	79	3.8 (3.1, 4.8)	-	-	-	-	-	-	2080	79	3.8 (3.1, 4.8)
	1-3	1956	265	17.0 (15.5, 18.7)	1956	265	13.7 (12.3, 15.3)	-	-	-	1956	265	17.0 (15.5, 18.7)
	3-5	1642	134	24.0 (22.1, 25.9)	1642	134	20.9 (19.2, 22.8)	-	-	-	1642	134	24.0 (22.1, 25.9)
	5-10	1435	198	37.1 (34.9, 39.4)	1435	198	34.6 (32.3, 36.9)	1435	198	17.3 (15.2, 19.6)	1435	198	37.1 (34.9, 39.4)

[a] Number of women at risk at start of time category [b] Number of events during time category [c] Cumulative risk at end of time category

Table S8. External validation exercise: Agreement of routinely collected data (RCD) and AZURE trial data for any recurrence. Calculations performed for all 2080 women in external validation cohort. The outcome of any recurrence is defined in Table S4.

	**Analysis period and trial arm**											
**Any recurrence**	**All time periods**			**>1 year after randomisation[a]**			**>5 years after randomisation[a]**			**All time periods with post-validation adjustment[b]**		
	**Control (N=1041)**	**Intervention (N=1039)**	**Total (N=2080)**	**Control (N=1041)**	**Intervention (N=1039)**	**Total (N=2080)**	**Control (N=1041)**	**Intervention (N=1039)**	**Total (N=2080)**	**Control (N=1041)**	**Intervention (N=1039)**	**Total (N=2080)**
	**No.**											
Event in both datasets	340	328	668	227	185	412	65	27	92	338	327	665
Event only in trial data	6	8	14	6	7	13	4	4	8	8	9	17
Event only in RCD	226	469	695	103	286	389	28	14	42	190	451	641
No event in either dataset	469	234	703	454	219	673	407	187	594	505	252	757
**Censored before analysis period**												
Only in trial data	0	0	0	14	19	33	17	8	25	0	0	0
Only in RCD	0	0	0	194	275	469	208	491	699	0	0	0
In both datasets	0	0	0	43	48	91	312	308	620	0	0	0
**Time difference when event present in both datasets**	**No. (%)**											
<6 months	211 (62.1%)	146 (44.5%)	357 (53.4%)	178 (78.4%)	111 (60.0%)	289 (70.1%)	57 (87.7%)	27 (100%)	84 (91.3%)	241 (71.3%)	157 (48.0%)	398 (59.8%)
6-12 months	23 (6.8%)	32 (9.8%)	55 (8.2%)	12 (5.3%)	17 (9.2%)	29 (7.0%)	3 (4.6%)	0 (0.0%)	3 (3.3%)	19 (5.6%)	34 (10.4%)	53 (8.0%)
>1 year	106 (31.2%)	150 (45.7%)	256 (38.3%)	37 (16.3%)	57 (30.8%)	94 (22.8%)	5 (7.7%)	0 (0.0%)	5 (5.4%)	78 (23.1%)	136 (41.6%)	214 (32.2%)
**Performance measures[c]**	**% (95% CI)**											
All time periods												
Sensitivity	98.3 (96.9, 99.6)	97.6 (96.0, 99.2)	97.9 (96.9, 99.0)	97.4 (95.4, 99.5)	96.4 (93.7, 99.0)	96.9 (95.3, 98.6)	94.2 (88.7, 99.7)	87.1 (75.3, 98.9)	92.0 (86.7, 97.3)	97.7 (96.1, 99.3)	97.3 (95.6, 99.0)	97.5 (96.3, 98.7)
Specificity	67.5 (64.0, 71.0)	33.3 (29.8, 36.8)	50.3 (47.7, 52.9)	81.5 (78.3, 84.7)	43.4 (39.0, 47.7)	63.4 (60.5, 66.3)	93.6 (91.3, 95.9)	93.0 (89.5, 96.6)	93.4 (91.5, 95.3)	72.7 (69.3, 76.0)	35.8 (32.3, 39.4)	54.1 (51.5, 56.8)
PPV	60.1 (56.0, 64.1)	41.2 (37.7, 44.6)	49.0 (46.4, 51.7)	68.8 (63.8, 73.8)	39.3 (34.9, 43.7)	51.4 (48.0, 54.9)	69.9 (60.6, 79.2)	65.9 (51.3, 80.4)	68.7 (60.8, 76.5)	64.0 (59.9, 68.1)	42.0 (38.6, 45.5)	50.9 (48.2, 53.6)
NPV	98.7 (97.7, 99.7)	96.7 (94.4, 98.9)	98.0 (97.0, 99.1)	98.7 (97.7, 99.7)	96.9 (94.6, 99.2)	98.1 (97.1, 99.1)	99.0 (98.1, 100)	97.9 (95.9, 99.9)	98.7 (97.8, 99.6)	98.4 (97.4, 99.5)	96.6 (94.3, 98.8)	97.8 (96.8, 98.8)
Within 6 months of trial data												
Sensitivity	61.0 (55.8, 66.1)	43.5 (38.2, 48.8)	52.3 (48.6, 56.1)	76.4 (70.9, 81.8)	57.8 (50.8, 64.8)	68.0 (63.6, 72.4)	82.6 (73.7, 91.6)	87.1 (75.3, 98.9)	84.0 (76.8, 91.2)	69.7 (64.8, 74.5)	46.7 (41.4, 52.1)	58.4 (54.7, 62.1)
Specificity	67.5 (64.0, 71.0)	33.3 (29.8, 36.8)	50.3 (47.7, 52.9)	81.5 (78.3, 84.7)	43.4 (39.0, 47.7)	63.4 (60.5, 66.3)	93.6 (91.3, 95.9)	93.0 (89.5, 96.6)	93.4 (91.5, 95.3)	72.7 (69.3, 76.0)	35.8 (32.3, 39.4)	54.1 (51.5, 56.8)
PPV	48.3 (43.6, 53.0)	23.7 (20.4, 27.1)	33.9 (31.1, 36.8)	63.3 (57.7, 69.0)	28.0 (23.5, 32.4)	42.6 (38.9, 46.3)	67.1 (57.1, 77.1)	65.9 (51.3, 80.4)	66.7 (58.4, 74.9)	55.9 (51.2, 60.6)	25.8 (22.3, 29.3)	38.3 (35.4, 41.3)
NPV	77.6 (74.3, 81.0)	55.2 (50.5, 59.9)	68.4 (65.5, 71.2)	89.2 (86.5, 91.9)	73.0 (68.0, 78.0)	83.2 (80.6, 85.8)	97.1 (95.5, 98.7)	97.9 (95.9, 99.9)	97.4 (96.1, 98.6)	82.8 (79.8, 85.8)	58.5 (53.8, 63.1)	72.7 (70.0, 75.4)

PPV, Positive predictive value; NPV, Negative predictive value. [a] Analysis period starts at 1 or 5 years after randomisation. [b] Post-validation adjustment ignores events reported in the RCD as locoregional recurrence, recurrence of unknown type or contralateral breast cancer during the first year after diagnosis. [c] Performance measures defined in footnote of Table 3

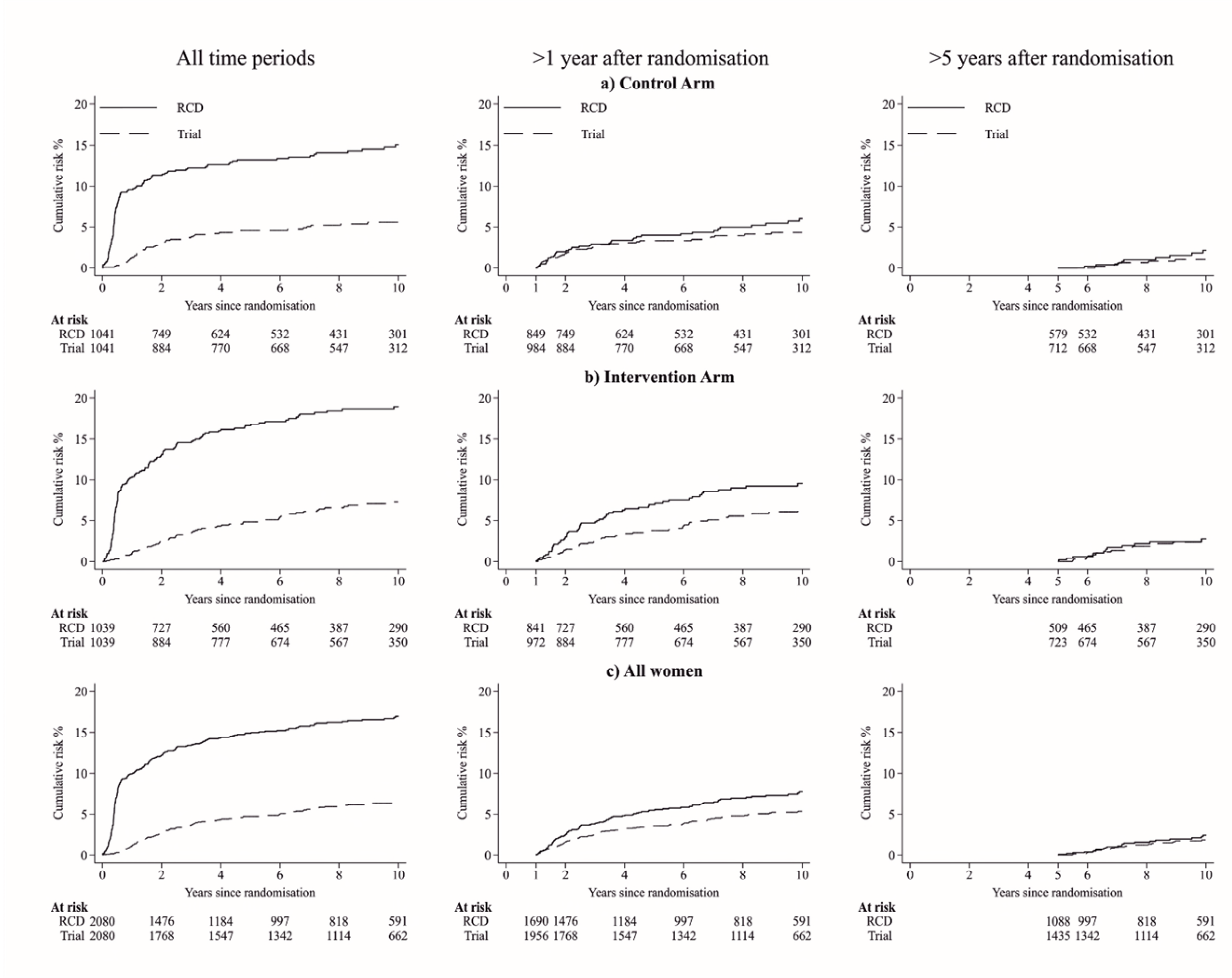

Figure S4. External validation exercise: Cumulative risk of locoregional recurrence in AZURE trial (dashed lines) and in routinely collected data (RCD) (solid lines). Analyses are by separate randomisation arm (a and b), and for all 2080 women included in external validation group (c). The outcome of locoregional recurrence is defined in Table S4. Plotted values at 1-, 3-, 5- and 10-years (and 95% confidence intervals) are in Table S9.

Table S9. External Validation exercise: Number of events and cumulative risks of locoregional recurrence by time since randomisation and AZURE trial arm, for routinely collected data (RCD) and AZURE trial data. Calculated for all 2080 women in the external validation cohort.

			All time periods			>1 year after randomisation			>5 years after randomisation		
**Trial Arm**	**Dataset**	**Time since randomisation (years)**	**No. women at risk[a]**	**No. events[b]**	**Cumulative risk[c] % (95% CI)**	**No. women at risk[a]**	**No. events[b]**	**Cumulative risk[c] % (95% CI)**	**No. women at risk[a]**	**No. events[b]**	**Cumulative risk[c] % (95% CI)**
**Control**	RCD	0-1	1041	94	9.5 (7.8, 11.5)	-	-	-	-	-	-
		1-3	849	23	12.2 (10.3, 14.4)	849	23	3.0 (2.0, 4.5)	-	-	-
		3-5	668	7	13.2 (11.1, 15.5)	668	7	4.1 (2.9, 5.8)	-	-	-
		5-10	579	9	14.9 (12.7, 17.5)	579	9	6.0 (4.4, 8.2)	579	9	2.0 (1.1, 3.9)
	Trial	0-1	1041	13	1.3 (0.7, 2.2)	-	-	-	-	-	-
		1-3	984	24	3.9 (2.8, 5.3)	984	24	2.6 (1.8, 3.9)	-	-	-
		3-5	817	6	4.6 (3.4, 6.2)	817	6	3.4 (2.4, 4.8)	-	-	-
		5-10	712	6	5.7 (4.3, 7.6)	712	6	4.5 (3.2, 6.3)	712	6	1.2 (0.5, 2.6)
**Intervention**	RCD	0-1	1039	102	10.3 (8.6, 12.4)				-	-	-
		1-3	841	36	14.6 (12.5, 17.0)	841	36	4.8 (3.5, 6.6)	-	-	-
		3-5	625	14	16.7 (14.4, 19.3)	625	14	7.1 (5.4, 9.3)	-	-	-
		5-10	509	12	19.1 (16.6, 22.1)	509	12	9.9 (7.7, 12.6)	509	12	3.0 (1.7, 5.2)
	Trial	0-1	1039	11	1.1 (0.6, 2.0)	-	-	-	-	-	-
		1-3	972	22	3.5 (2.5, 4.9)	972	22	2.4 (1.6, 3.7)	-	-	-
		3-5	825	11	4.8 (3.6, 6.5)	825	11	3.8 (2.7, 5.3)	-	-	-
		5-10	723	16	7.6 (5.9, 9.8)	723	16	6.6 (5.0, 8.7)	723	16	2.9 (1.8, 4.8)
**Total**	RCD	0-1	2080	196	9.9 (8.6, 11.3)	-	-	-	-	-	-
		1-3	1690	59	13.4 (11.9, 15.0)	1690	59	3.9 (3.0, 5.0)	-	-	-
		3-5	1293	21	14.9 (13.3, 16.6)	1293	21	5.6 (4.5, 6.9)	-	-	-
		5-10	1088	21	17.0 (15.3, 18.9)	1088	21	7.9 (6.5, 9.6)	1088	21	2.5 (1.6, 3.8)
	Trial	0-1	2080	24	1.2 (0.8, 1.8)	-	-	-	-	-	-
		1-3	1956	46	3.7 (2.9, 4.6)	1956	46	2.5 (1.9, 3.4)	-	-	-
		3-5	1642	17	4.7 (3.9, 5.8)	1642	17	3.6 (2.8, 4.6)	-	-	-
		5-10	1435	22	6.7 (5.6, 8.1)	1435	22	5.6 (4.5, 6.9)	1435	22	2.1 (1.4, 3.1)

[a] Number of women at risk at start of time category
[b] Number of events during time category
[c] Cumulative risk at end of time category

Table S10. External validation exercise: Agreement of routinely collected data (RCD) and AZURE trial data for locoregional recurrence. Calculations performed for all 2080 women in external validation cohort.

Locoregional recurrence[a]	Analysis period and trial arm								
	All time periods			>1 year after randomisation[b]			>5 years after randomisation[b]		
	Control (N=1041)	Intervention (N=1039)	Total (N=2080)	Control (N=1041)	Intervention (N=1039)	Total (N=2080)	Control (N=1041)	Intervention (N=1039)	Total (N=2080)
					No.				
Event in both datasets	23	31	54	16	21	37	2	4	6
Event only in trial data	26	29	55	15	19	34	3	3	6
Event only in RCD	110	133	243	22	39	61	6	8	14
No event in either dataset	882	846	1728	780	740	1520	545	477	1022
**Censored before analysis period**									
Only in trial data	0	0	0	16	22	38	23	17	40
Only in RCD	0	0	0	151	153	304	156	231	387
In both datasets	0	0	0	41	45	86	306	299	605
**Time difference when event present in both datasets**					No. (%)				
<6 months	15 (65.2%)	24 (77.4%)	39 (72.2%)	13 (81.2%)	19 (90.5%)	32 (86.5%)	2 (100%)	4 (100%)	6 (100%)
6-12 months	5 (21.7%)	0 (0.0%)	5 (9.3%)	2 (12.5%)	0 (0.0%)	2 (5.4%)	0 (0.0%)	0 (0.0%)	0 (0.0%)
>1 year	3 (13.0%)	7 (22.6%)	10 (18.5%)	1 (6.2%)	2 (9.5%)	3 (8.1%)	0 (0.0%)	0 (0.0%)	0 (0.0%)
**Performance measures[c]**					% (95% CI)				
All time periods									
Sensitivity	46.9 (33.0, 60.9)	51.7 (39.0, 64.3)	49.5 (40.2, 58.9)	51.6 (34.0, 69.2)	52.5 (37.0, 68.0)	52.1 (40.5, 63.7)	40.0 (0.0, 82.9)	57.1 (20.5, 93.8)	50.0 (21.7, 78.3)
Specificity	88.9 (87.0, 90.9)	86.4 (84.3, 88.6)	87.7 (86.2, 89.1)	97.3 (96.1, 98.4)	95.0 (93.5, 96.5)	96.1 (95.2, 97.1)	98.9 (98.0, 99.8)	98.4 (97.2, 99.5)	98.6 (97.9, 99.4)
PPV	17.3 (10.9, 23.7)	18.9 (12.9, 24.9)	18.2 (13.8, 22.6)	42.1 (26.4, 57.8)	35.0 (22.9, 47.1)	37.8 (28.2, 47.4)	25.0 (0.0, 55.0)	33.3 (6.7, 60.0)	30.0 (9.9, 50.1)
NPV	97.1 (96.1, 98.2)	96.7 (95.5, 97.9)	96.9 (96.1, 97.7)	98.1 (97.2, 99.1)	97.5 (96.4, 98.6)	97.8 (97.1, 98.5)	99.5 (98.8, 100)	99.4 (98.7, 100)	99.4 (99.0, 99.9)
Within 6 months of trial data									
Sensitivity	30.6 (17.7, 43.5)	40.0 (27.6, 52.4)	35.8 (26.8, 44.8)	41.9 (24.6, 59.3)	47.5 (32.0, 63.0)	45.1 (33.5, 56.6)	40.0 (0.0, 82.9)	57.1 (20.5, 93.8)	50.0 (21.7, 78.3)
Specificity	88.9 (87.0, 90.9)	86.4 (84.3, 88.6)	87.7 (86.2, 89.1)	97.3 (96.1, 98.4)	95.0 (93.5, 96.5)	96.1 (95.2, 97.1)	98.9 (98.0, 99.8)	98.4 (97.2, 99.5)	98.6 (97.9, 99.4)
PPV	12.0 (6.3, 17.7)	15.3 (9.7, 20.9)	13.8 (9.8, 17.9)	37.1 (21.1, 53.2)	32.8 (20.7, 44.8)	34.4 (24.8, 44.1)	25.0 (0.0, 55.0)	33.3 (6.7, 60.0)	30.0 (9.9, 50.1)
NPV	96.3 (95.1, 97.5)	95.9 (94.6, 97.2)	96.1 (95.2, 97.0)	97.7 (96.7, 98.8)	97.2 (96.1, 98.4)	97.5 (96.7, 98.3)	99.5 (98.8, 100)	99.4 (98.7, 100)	99.4 (99.0, 99.9)

PPV, Positive predictive value; NPV, Negative predictive value

[a] Women are censored at contralateral breast cancer, distant metastases, non-breast cancer malignancies and death (Table S4)

[b] Analysis period starts at 1 or 5 years after randomisation

[c] Performance measures defined in footnote of Table 3

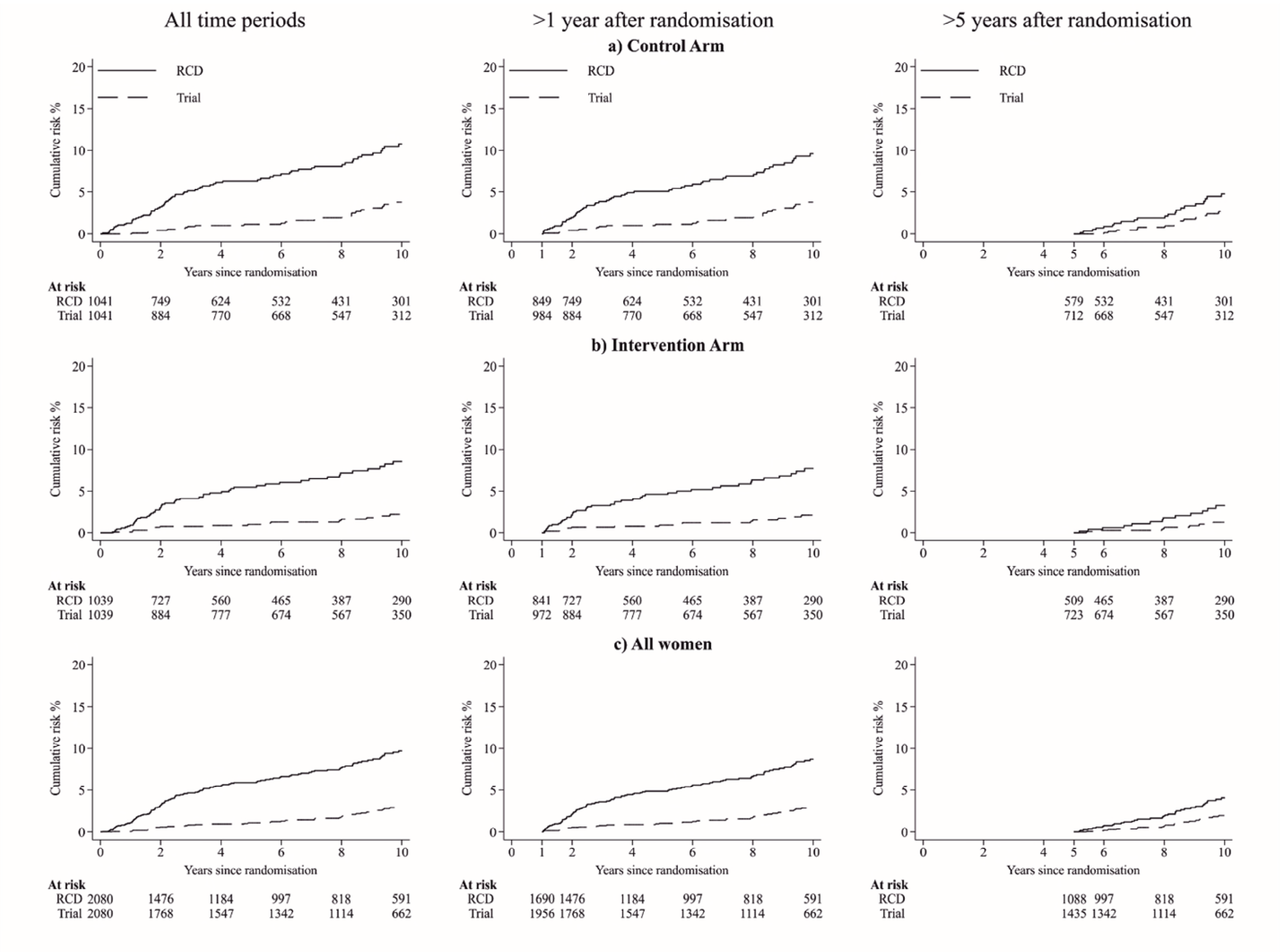

Figure S5. External validation exercise: Cumulative risk of contralateral breast cancer in AZURE trial (dashed lines) and in routinely collected data (RCD) (solid lines). Analyses are by separate randomisation arm (a and b), and for all 2080 women included in external validation group (c). The outcome of contralateral breast cancer is defined in Table S4. Plotted values at 1-, 3-, 5- and 10-years (and 95% confidence intervals) are in Table S11.

Table S11. External validation exercise: Number of events and cumulative risks of contralateral breast cancer by time since randomisation and AZURE trial arm, for routinely collected data (RCD) and trial data. Calculated for all 2080 women in the external validation cohort.

			All time periods			>1 year after randomisation			>5 years after randomisation		
**Trial Arm**	**Dataset**	**Time since randomisation (years)**	**No. women at risk[a]**	**No. events[b]**	**Cumulative risk[c] % (95% CI)**	**No. women at risk[a]**	**No. events[b]**	**Cumulative risk[c] % (95% CI)**	**No. women at risk[a]**	**No. events[b]**	**Cumulative risk[c] % (95% CI)**
**Control**	RCD	0-1	1041	12	1.3 (0.7, 2.2)	-	-	-	-	-	-
		1-3	849	30	5.1 (3.8, 6.8)	849	30	3.9 (2.7, 5.5)	-	-	-
		3-5	668	8	6.3 (4.8, 8.2)	668	8	5.1 (3.7, 7.0)	-	-	-
		5-10	579	21	10.7 (8.5, 13.4)	579	21	9.5 (7.4, 12.2)	579	21	4.7 (3.1, 7.1)
	Trial	0-1	1041	0	0.0 (NA[d])	-	-	-	-	-	-
		1-3	984	8	0.9 (0.4, 1.8)	984	8	0.9 (0.4, 1.8)	-	-	-
		3-5	817	2	1.1 (0.6, 2.1)	817	2	1.1 (0.6, 2.1)	-	-	-
		5-10	712	14	3.8 (2.5, 5.7)	712	14	3.8 (2.5, 5.7)	712	14	2.7 (1.6, 4.5)
**Intervention**	RCD	0-1	1039	8	0.8 (0.4, 1.7)	-	-	-	-	-	-
		1-3	841	25	4.2 (3.0, 5.8)	841	25	3.4 (2.3, 4.9)	-	-	-
		3-5	625	8	5.5 (4.1, 7.4)	625	8	4.7 (3.4, 6.6)	-	-	-
		5-10	509	13	8.5 (6.5, 11.1)	509	13	7.8 (5.8, 10.3)	509	13	3.2 (1.9, 5.5)
	Trial	0-1	1039	1	0.1 (0.0, 0.7)	-	-	-	-	-	-
		1-3	972	6	0.8 (0.4, 1.6)	972	6	0.7 (0.3, 1.5)	-	-	-
		3-5	825	2	1.0 (0.5, 2.0)	825	2	0.9 (0.5, 1.8)	-	-	-
		5-10	723	7	2.3 (1.4, 3.8)	723	7	2.2 (1.3, 3.7)	723	7	1.3 (0.6, 2.7)
**Total**	RCD	0-1	2080	20	1.1 (0.7, 1.6)	-	-	-	-	-	-
		1-3	1690	55	4.6 (3.7, 5.8)	1690	55	3.6 (2.8, 4.7)	-	-	-
		3-5	1293	16	5.9 (4.8, 7.2)	1293	16	4.9 (3.9, 6.2)	-	-	-
		5-10	1088	34	9.6 (8.1, 11.5)	1088	34	8.7 (7.2, 10.5)	1088	34	4.0 (2.9, 5.5)
	Trial	0-1	2080	1	0.0 (0.0, 0.4)	-	-	-	-	-	-
		1-3	1956	14	0.8 (0.5, 1.4)	1956	14	0.8 (0.5, 1.3)	-	-	-
		3-5	1642	4	1.1 (0.7, 1.7)	1642	4	1.0 (0.7, 1.6)	-	-	-
		5-10	1435	21	3.0 (2.2, 4.2)	1435	21	3.0 (2.2, 4.1)	1435	21	2.0 (1.3, 3.0)

[a] Number of women at risk at start of time category [b] Number of events during time category [c] Cumulative risk at end of time category
[d] Unable to calculate confidence interval when no events are observed

Table S12. External validation exercise: Agreement of routinely collected data (RCD) and AZURE trial data for contralateral breast cancer. Calculations performed for all 2080 women in external validation cohort.

Contralateral breast cancer[a]	Analysis period and trial arm								
	All time periods			>1 year after randomisation[b]			>5 years after randomisation[b]		
	Control (N=1041)	Intervention (N=1039)	Total (N=2080)	Control (N=1041)	Intervention (N=1039)	Total (N=2080)	Control (N=1041)	Intervention (N=1039)	Total (N=2080)
					No.				
Event in both datasets	18	11	29	18	10	28	13	4	17
Event only in trial data	6	5	11	3	4	7	1	1	2
Event only in RCD	53	43	96	41	35	76	8	7	15
No event in either dataset	964	980	1944	771	770	1541	534	480	1014
**Censored before analysis period**									
Only in trial data	0	0	0	16	22	38	23	17	40
Only in RCD	0	0	0	151	153	304	156	231	387
In both datasets	0	0	0	41	45	86	306	299	605
**Time difference when event present in both datasets**					No. (%)				
<6 months	18 (100%)	11 (100%)	29 (100%)	18 (100%)	10 (100%)	28 (100%)	13 (100%)	4 (100%)	17 (100%)
6-12 months	0 (0.0%)	0 (0.0%)	0 (0.0%)	0 (0.0%)	0 (0.0%)	0 (0.0%)	0 (0.0%)	0 (0.0%)	0 (0.0%)
>1 year	0 (0.0%)	0 (0.0%)	0 (0.0%)	0 (0.0%)	0 (0.0%)	0 (0.0%)	0 (0.0%)	0 (0.0%)	0 (0.0%)
**Performance measures[c]**					% (95% CI)				
All time periods									
Sensitivity	75.0 (57.7, 92.3)	68.8 (46.0, 91.5)	72.5 (58.7, 86.3)	85.7 (70.7, 100)	71.4 (47.8, 95.1)	80.0 (66.7, 93.3)	92.9 (79.4, 100)	80.0 (44.9, 100)	89.5 (75.7, 100)
Specificity	94.8 (93.4, 96.2)	95.8 (94.6, 97.0)	95.3 (94.4, 96.2)	95.0 (93.4, 96.5)	95.7 (94.2, 97.1)	95.3 (94.3, 96.3)	98.5 (97.5, 99.5)	98.6 (97.5, 99.6)	98.5 (97.8, 99.3)
PPV	25.4 (15.2, 35.5)	20.4 (9.6, 31.1)	23.2 (15.8, 30.6)	30.5 (18.8, 42.3)	22.2 (10.1, 34.4)	26.9 (18.4, 35.4)	61.9 (41.1, 82.7)	36.4 (7.9, 64.8)	53.1 (35.8, 70.4)
NPV	99.4 (98.9, 99.9)	99.5 (99.0, 99.9)	99.4 (99.1, 99.8)	99.6 (99.2, 100)	99.5 (99.0, 100)	99.5 (99.2, 99.9)	99.8 (99.4, 100)	99.8 (99.4, 100)	99.8 (99.5, 100)
Within 6 months of trial data									
Sensitivity	75.0 (57.7, 92.3)	68.8 (46.0, 91.5)	72.5 (58.7, 86.3)	85.7 (70.7, 100)	71.4 (47.8, 95.1)	80.0 (66.7, 93.3)	92.9 (79.4, 100)	80.0 (44.9, 100)	89.5 (75.7, 100)
Specificity	94.8 (93.4, 96.2)	95.8 (94.6, 97.0)	95.3 (94.4, 96.2)	95.0 (93.4, 96.5)	95.7 (94.2, 97.1)	95.3 (94.3, 96.3)	98.5 (97.5, 99.5)	98.6 (97.5, 99.6)	98.5 (97.8, 99.3)
PPV	25.4 (15.2, 35.5)	20.4 (9.6, 31.1)	23.2 (15.8, 30.6)	30.5 (18.8, 42.3)	22.2 (10.1, 34.4)	26.9 (18.4, 35.4)	61.9 (41.1, 82.7)	36.4 (7.9, 64.8)	53.1 (35.8, 70.4)
NPV	99.4 (98.9, 99.9)	99.5 (99.0, 99.9)	99.4 (99.1, 99.8)	99.6 (99.2, 100)	99.5 (99.0, 100)	99.5 (99.2, 99.9)	99.8 (99.4, 100)	99.8 (99.4, 100)	99.8 (99.5, 100)

PPV, Positive predictive value; NPV, Negative predictive value

[a] Women are censored at locoregional recurrence, distant metastases, non-breast cancer malignancies and death (Table S4)

[b] Analysis period starts at 1 or 5 years after randomisation

[c] Performance measures defined in footnote of Table 3

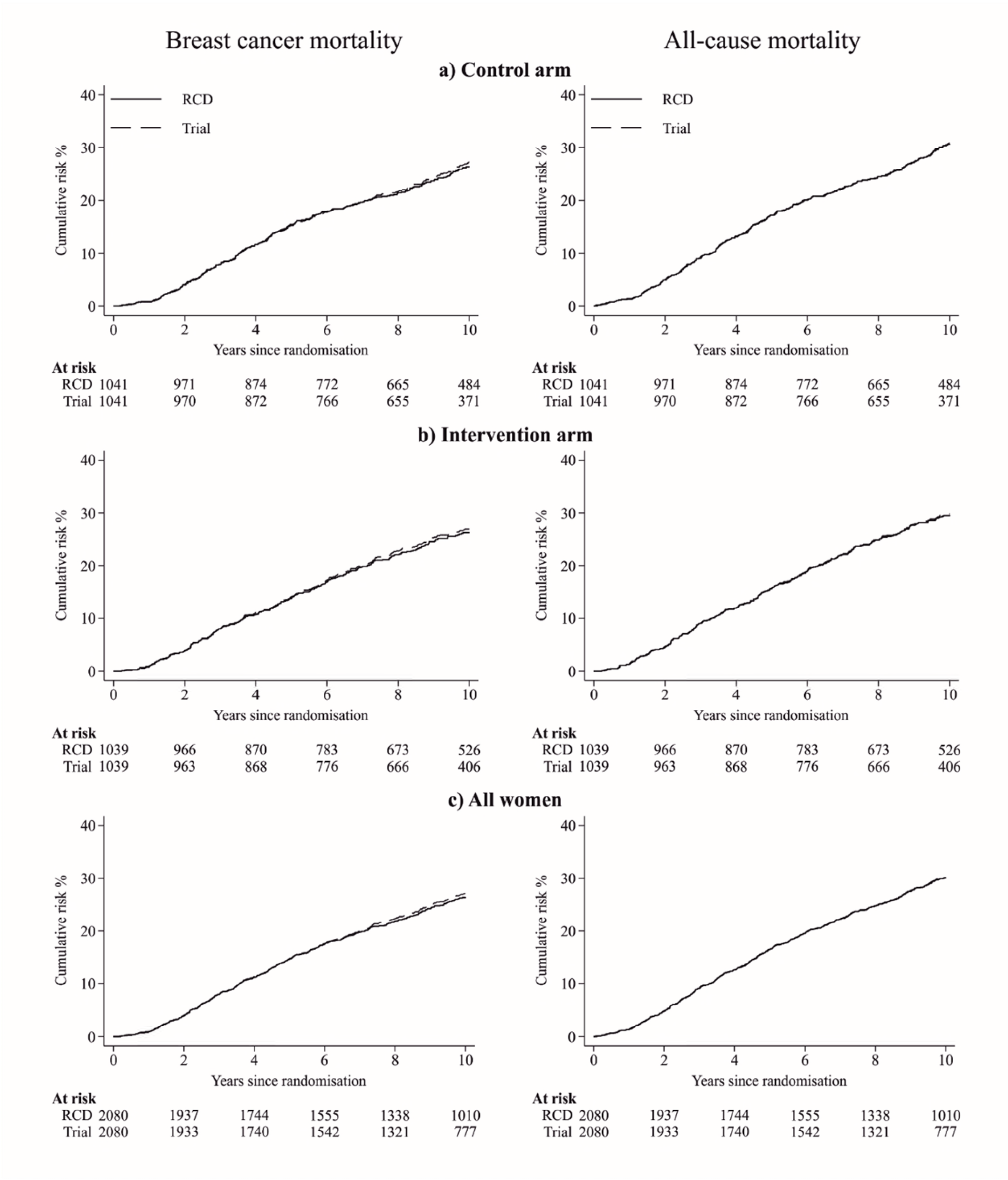

Figure S6. External validation exercise: Cumulative risk of breast cancer mortality and all-cause mortality in AZURE trial (dashed lines) and in routinely collected data (RCD) (solid lines). Analyses are by separate randomisation arm (a and b), and for all 2080 women included in the external validation group (c). The outcomes of breast cancer mortality and all-cause mortality are defined in Table S4. Plotted values at 1-, 3-, 5- and 10-years (and 95% confidence intervals) are in Table S13.

Table S13. External validation exercise: Number of events and cumulative risks of breast cancer mortality and all-cause mortality by time since randomisation and AZURE trial arm, for routinely collected data (RCD) and AZURE trial data. Calculated for all 2080 women in the external validation cohort.

Trial Arm	Dataset	Time since randomisation (years)	Breast cancer mortality: No. women at risk[a]	Breast cancer mortality: No. events[b]	Breast cancer mortality: Cumulative risk[c] % (95% CI)	All-cause mortality: No. women at risk[a]	All-cause mortality: No. events[b]	All-cause mortality: Cumulative risk[c] % (95% CI)
**Control**	RCD	0-1	1041	9	0.9 (0.5, 1.7)	1041	14	1.4 (0.8, 2.3)
		1-3	1019	71	7.8 (6.4, 9.7)	1019	79	9.0 (7.4, 11.0)
		3-5	927	75	15.4 (13.3, 17.8)	927	82	17.2 (15.0, 19.7)
		5-10	821	92	26.6 (23.8, 29.6)	821	117	30.8 (28.0, 33.9)
	Trial	0-1	1041	8	0.8 (0.4, 1.5)	1041	14	1.4 (0.8, 2.3)
		1-3	1015	73	8.0 (6.5, 9.8)	1015	80	9.2 (7.6, 11.1)
		3-5	925	72	15.3 (13.2, 17.7)	925	80	17.2 (15.0, 19.6)
		5-10	813	99	28.4 (25.5, 31.5)	813	117	32.1 (29.1, 35.3)
**Intervention**	RCD	0-1	1039	10	1.0 (0.5, 1.8)	1039	14	1.4 (0.8, 2.3)
		1-3	1014	71	8.0 (6.5, 9.9)	1014	78	9.0 (7.4, 11.0)
		3-5	914	57	13.8 (11.8, 16.1)	914	66	15.7 (13.6, 18.1)
		5-10	827	110	26.8 (24.0, 29.8)	827	126	30.0 (27.2, 33.1)
	Trial	0-1	1039	8	0.8 (0.4, 1.5)	1039	14	1.4 (0.8, 2.3)
		1-3	1012	73	8.0 (6.5, 9.9)	1012	78	9.0 (7.4, 11.0)
		3-5	912	59	14.1 (12.1, 16.4)	912	66	15.7 (13.6, 18.1)
		5-10	826	113	28.5 (25.6, 31.7)	826	127	31.5 (28.5, 34.6)
**Total**	RCD	0-1	2080	19	0.9 (0.6, 1.4)	2080	28	1.4 (0.9, 2.0)
		1-3	2033	142	7.9 (6.8, 9.2)	2033	157	9.0 (7.9, 10.4)
		3-5	1841	132	14.6 (13.2, 16.3)	1841	148	16.4 (14.9, 18.1)
		5-10	1648	202	26.7 (24.7, 28.8)	1648	243	30.4 (28.4, 32.6)
	Trial	0-1	2080	16	0.8 (0.5, 1.3)	2080	28	1.4 (0.9, 2.0)
		1-3	2027	146	8.0 (6.9, 9.3)	2027	158	9.1 (7.9, 10.4)
		3-5	1837	131	14.7 (13.2, 16.3)	1837	146	16.4 (14.9, 18.1)
		5-10	1639	212	28.4 (26.4, 30.7)	1639	244	31.8 (29.6, 34.0)

[a] Number of women at risk at the start of the time category
[b] Number of events during time category
[c] Cumulative risk at end of time category

Table S14. External validation exercise: Agreement of routinely collected data (RCD) and AZURE trial data for breast cancer mortality and all-cause mortality, by AZURE trial arm. Calculations performed for all 2080 women in external validation cohort.

	**Outcome and trial arm**					
	**Breast cancer mortality[a]**			**All-cause mortality**		
	**Control (N=1041)**	**Intervention (N=1039)**	**Total (N=2080)**	**Control (N=1041)**	**Intervention (N=1039)**	**Total (N=2080)**
			**No.**			
Event in both datasets	242	247	489	299	289	588
Event only in trial data	17	8	25	0	1	1
Event only in RCD	11	4	15	1	1	2
No event in either dataset	771	780	1551	741	748	1489
**Time difference when event present in both datasets**			**No. (%)**			
<6 months	242 (100%)	246 (99.6%)	488 (99.8%)	299 (100%)	288 (99.7%)	587 (99.8%)
6-12 months	0 (0.0%)	0 (0.0%)	0 (0.0%)	0 (0.0%)	0 (0.0%)	0 (0.0%)
>1 year	0 (0.0%)	1 (0.4%)	1 (0.2%)	0 (0.0%)	1 (0.3%)	1 (0.2%)
**Performance measures[b]**			**% (95% CI)**			
All time periods						
Sensitivity	93.4 (90.4, 96.5)	96.9 (94.7, 99.0)	95.1 (93.3, 97.0)	100 (100, 100)	99.7 (99.0, 100)	99.8 (99.5, 100)
Specificity	98.6 (97.8, 99.4)	99.5 (99.0, 100)	99.0 (98.6, 99.5)	99.9 (99.6, 100)	99.9 (99.6, 100)	99.9 (99.7, 100)
PPV	95.7 (93.1, 98.2)	98.4 (96.9, 100)	97.0 (95.5, 98.5)	99.7 (99.0, 100)	99.7 (99.0, 100)	99.7 (99.2, 100)
NPV	97.8 (96.8, 98.9)	99.0 (98.3, 99.7)	98.4 (97.8, 99.0)	100 (100, 100)	99.9 (99.6, 100)	99.9 (99.8, 100)
Within 6 months of trial data						
Sensitivity	93.4 (90.4, 96.5)	96.5 (94.2, 98.7)	94.9 (93.0, 96.8)	100 (100, 100)	99.3 (98.4, 100)	99.7 (99.2, 100)
Specificity	98.6 (97.8, 99.4)	99.5 (99.0, 100)	99.0 (98.6, 99.5)	99.9 (99.6, 100)	99.9 (99.6, 100)	99.9 (99.7, 100)
PPV	95.7 (93.1, 98.2)	98.4 (96.8, 100)	97.0 (95.5, 98.5)	99.7 (99.0, 100)	99.7 (99.0, 100)	99.7 (99.2, 100)
NPV	97.8 (96.8, 98.9)	98.9 (98.1, 99.6)	98.4 (97.7, 99.0)	100 (100, 100)	99.7 (99.4, 100)	99.9 (99.7, 100)

PPV, Positive predictive value; NPV, Negative predictive value

[a] Women are censored at non-breast-cancer deaths (Table S4)

[b] Performance measures defined in footnote of Table 3

Table S15a. Training exercise after initial development of algorithm using WMCIU database: Comparison of first outcome events identified by algorithm using routinely collected data (RCD) with first events recorded in the AZURE training sample of 150 women, by trial arm.

AZURE	Algorithm based on routinely collected data								
	Loco-regional recurrence	Distant recurrence	Recurrence, type unknown	Contra-lateral breast cancer	Death from breast cancer	Non-breast cancer malignancy	Non-breast-cancer death	No event recorded	Total
a) **Control arm**									
Locoregional recurrence	**3**	3	**2**	0	0	0	0	0	8
Distant recurrence	2	**16**	**9**	2	2	0	0	0	31
Contralateral breast cancer	1	1	0	**0**	0	0	0	0	2
Death from breast cancer	0	0	0	0	**0**	0	0	0	0
Non-breast cancer malignancy	0	1	0	0	0	**3**	0	0	4
Non-breast-cancer death	0	0	0	0	0	0	**3**	0	3
No event recorded	3	5	8	1	0	0	0	**0**	17
Total	9	26	19	3	2	3	3	0	65
b) **Intervention arm**									
Locoregional recurrence	**8**	3	**2**	0	0	0	0	0	13
Distant recurrence	5	**13**	**10**	1	2	0	1	0	32
Contralateral breast cancer	0	0	1	**3**	0	0	0	0	4
Death from breast cancer	0	0	0	0	**0**	0	0	0	0
Non-breast cancer malignancy	1	0	0	0	0	**1**	0	0	2
Non-breast-cancer death	0	0	0	0	0	1	**0**	0	1
No event recorded	3	3	27	0	0	0	0	**0**	33
Total	17	19	40	4	2	2	1	0	85
c) **All women**									
Locoregional recurrence	**11**	6	**4**	0	0	0	0	0	21
Distant recurrence	7	**29**	**19**	3	4	0	1	0	63
Contralateral breast cancer	1	1	1	**3**	0	0	0	0	6
Death from breast cancer	0	0	0	0	**0**	0	0	0	0
Non-breast cancer malignancy	1	1	0	0	0	**4**	0	0	6
Non-breast-cancer death	0	0	0	0	0	1	**3**	0	4
No event recorded	6	8	35	1	0	0	0	**0**	50
Total	26	45	59	7	4	5	4	0	150

Entries in the table in bold type and with shading indicate that the outcome using the algorithm based on the RCD and the outcome based on the AZURE Training Sample were judged to be in agreement.

Table S15b. Training exercise after further training using 150 women from AZURE trial: Comparison of first outcome events identified by algorithm using routinely collected data (RCD) with first events recorded in the AZURE training sample of 150 women, by trial arm.

AZURE	Algorithm based on routinely collected data								Total
	Loco-regional recurrence	Distant recurrence	Recurrence, type unknown	Contra-lateral breast cancer	Death from breast cancer	Non-breast cancer malignancy	Non-breast-cancer death	No event recorded	
a) **Control arm**									
Locoregional recurrence	**3**	3	**2**	0	0	0	0	0	8
Distant recurrence	3	**20**	**6**	1	2	0	0	0	31
Contralateral breast cancer	1	1	0	**0**	0	0	0	0	2
Death from breast cancer	0	0	0	0	**0**	0	0	0	0
Non-breast cancer malignancy	0	1	0	0	0	**3**	0	0	4
Non-breast-cancer death	0	0	0	0	0	0	**3**	0	3
No event recorded	1	0	2	1	0	0	0	**13**	17
Total	7	25	10	2	2	3	3	13	65
b) **Intervention arm**									
Locoregional recurrence	**7**	2	**2**	2	0	0	0	0	13
Distant recurrence	5	**16**	**4**	3	4	0	0	0	32
Contralateral breast cancer	0	0	0	**4**	0	0	0	0	4
Death from breast cancer	0	0	0	0	**0**	0	0	0	0
Non-breast cancer malignancy	1	0	0	0	0	**1**	0	0	2
Non-breast-cancer death	0	0	0	0	0	1	**0**	0	1
No event recorded	2	2	2	0	0	0	0	**27**	33
Total	15	20	8	9	4	2	0	27	85
c) **All women**									
Locoregional recurrence	**10**	5	**4**	2	0	0	0	0	21
Distant recurrence	7	**36**	**10**	4	6	0	0	0	63
Contralateral breast cancer	1	1	0	**4**	0	0	0	0	6
Death from breast cancer	0	0	0	0	**0**	0	0	0	0
Non-breast cancer malignancy	1	1	0	0	0	**4**	0	0	6
Non-breast-cancer death	0	0	0	0	0	1	**3**	0	4
No event recorded	3	2	4	1	0	0	0	**40**	50
Total	22	45	18	11	6	5	3	40	150

Entries in the table in bold type and with shading indicate that the outcome using the algorithm based on the RCD and the outcome based on the AZURE Training Sample were judged to be in agreement.

Table S16. Internal validation exercise: Comparison of first outcome events identified by algorithm using routinely collected data (RCD) with first events recorded in the AZURE trial, for women in the control arm (a), intervention arm (b), and for all women in the internal validation group (c).

AZURE	Algorithm based on routinely collected data								Total
	**Locoregional recurrence**	**Distant recurrence**	**Recurrence, type unknown**	**Contralateral breast cancer**	**Death from breast cancer**	**Non-breast cancer malignancy**	**Non-breast-cancer death**	**No event recorded**	
a) **Control arm**									
Locoregional recurrence	**17**	7	**6**	5	2	1	0	3	41
Distant recurrence	29	**157**	**26**	11	8	0	0	4	235
Contralateral breast cancer	3	0	0	**18**	0	0	0	1	22
Death from breast cancer	1	4	0	0	**1**	0	1	0	7
Non-breast cancer malignancy	2	7	0	0	0	**25**	0	7	41
Non-breast-cancer death	0	1	0	1	2	0	**12**	0	16
No event recorded	34	18	16	18	0	5	0	**523**	614
Total	86	194	48	53	13	31	13	538	976
b) **Intervention arm**									
Locoregional recurrence	**18**	13	**6**	5	1	1	1	2	47
Distant recurrence	27	**149**	**29**	1	10	3	1	2	222
Contralateral breast cancer	2	2	0	**8**	0	0	0	0	12
Death from breast cancer	0	4	0	0	**2**	0	0	0	6
Non-breast cancer malignancy	3	4	1	1	0	**15**	0	7	31
Non-breast-cancer death	3	4	2	1	1	0	**8**	1	20
No event recorded	59	54	54	13	0	3	0	**433**	616
Total	112	230	92	29	14	22	10	445	954
c) **Overall**									
Locoregional recurrence	**35**	20	**12**	10	3	2	1	5	88
Distant recurrence	56	**306**	**55**	12	18	3	1	6	457
Contralateral breast cancer	5	2	0	**26**	0	0	0	1	34
Death from breast cancer	1	8	0	0	**3**	0	1	0	13
Non-breast cancer malignancy	5	11	1	1	0	**40**	0	14	72
Non-breast-cancer death	3	5	2	2	3	0	**20**	1	36
No event recorded	93	72	70	31	0	8	0	**956**	1230
Total	198	424	140	82	27	53	23	983	1930

Entries in the table in bold type and with shading indicate that the outcome using the algorithm based on the RCD and the outcome based on the AZURE Training Sample were judged to be in agreement.

Table S17. Internal validation exercise: Number of events and cumulative risks of distant recurrence by time since randomisation and AZURE trial arm, for routinely collected data (RCD) and AZURE trial data. Calculated for all 1930 women in the internal validation group.

			All time periods			>5 years after randomisation		
**Trial Arm**	**Dataset**	**Time since randomisation (years)**	**No. women at risk[a]**	**No. events[b]**	**Cumulative risk[c] % (95% CI)**	**No. women at risk[a]**	**No. events[b]**	**Cumulative risk[c] % (95% CI)**
**Control**	RCD	0-1	976	25	2.6 (1.7, 3.8)	-	-	-
		1-3	938	105	13.6 (11.6, 15.9)	-	-	-
		3-5	812	58	19.9 (17.5, 22.6)	-	-	-
		5-10	728	107	33.8 (30.7, 37.1)	728	107	17.4 (14.6, 20.6)
	Trial	0-1	976	24	2.5 (1.7, 3.7)	-	-	-
		1-3	933	111	14.2 (12.1, 16.6)	-	-	-
		3-5	803	53	20.0 (17.6, 22.7)	-	-	-
		5-10	707	88	32.7 (29.6, 36.1)	707	88	15.8 (13.1, 19.2)
**Intervention**	RCD	0-1	954	26	2.7 (1.9, 4.0)	-	-	-
		1-3	911	127	16.5 (14.3, 19.0)	-	-	-
		3-5	760	91	26.7 (23.9, 29.7)	-	-	-
		5-10	638	85	37.9 (34.7, 41.2)	638	85	15.2 (12.5, 18.5)
	Trial	0-1	954	27	2.9 (2.0, 4.1)	-	-	-
		1-3	905	89	12.6 (10.6, 14.9)	-	-	-
		3-5	789	57	19.0 (16.6, 21.7)	-	-	-
		5-10	700	87	31.4 (28.3, 34.8)	700	87	15.4 (12.6, 18.6)
**Total**	RCD	0-1	1930	51	2.7 (2.0, 3.5)	-	-	-
		1-3	1849	232	15.0 (13.5, 16.7)	-	-	-
		3-5	1572	149	23.2 (21.4, 25.2)	-	-	-
		5-10	1366	192	35.8 (33.6, 38.1)	1366	192	16.4 (14.4, 18.6)
	Trial	0-1	1930	51	2.7 (2.0, 3.5)	-	-	-
		1-3	1838	200	13.4 (11.9, 15.0)	-	-	-
		3-5	1592	110	19.5 (17.8, 21.4)	-	-	-
		5-10	1407	175	32.1 (29.8, 34.4)	1407	175	15.6 (13.6, 17.9)

[a] Number of women at risk at start of time category
[b] Number of events during time category
[c] Cumulative risk at end of time category

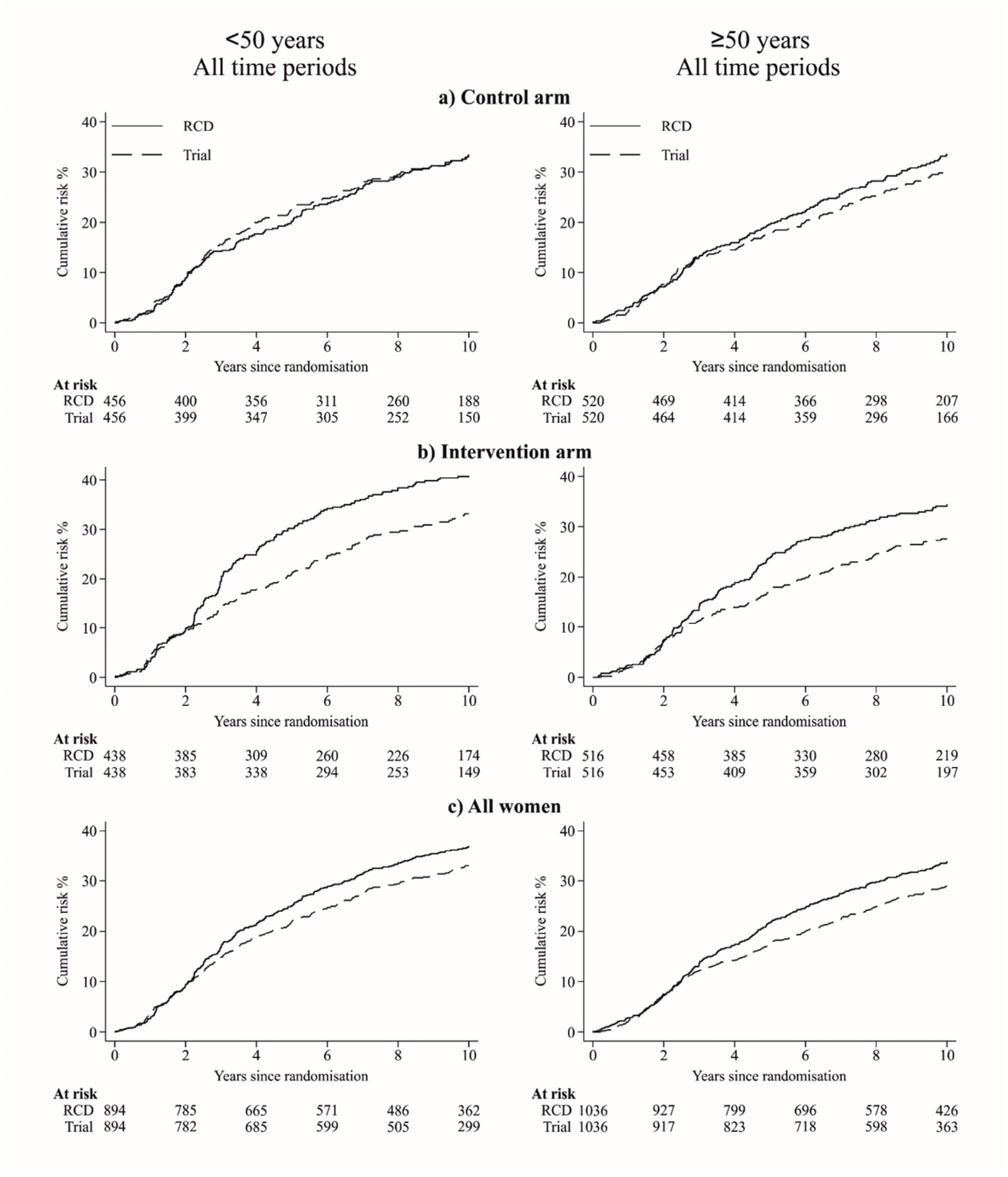

Figure S7. Internal validation exercise: Cumulative risk of distant recurrence in AZURE trial (dashed lines) and in the routinely collected data (RCD) (solid lines), split by age at randomisation. Analyses are by randomisation arm (a and b), and for all women included in the internal validation group (c).

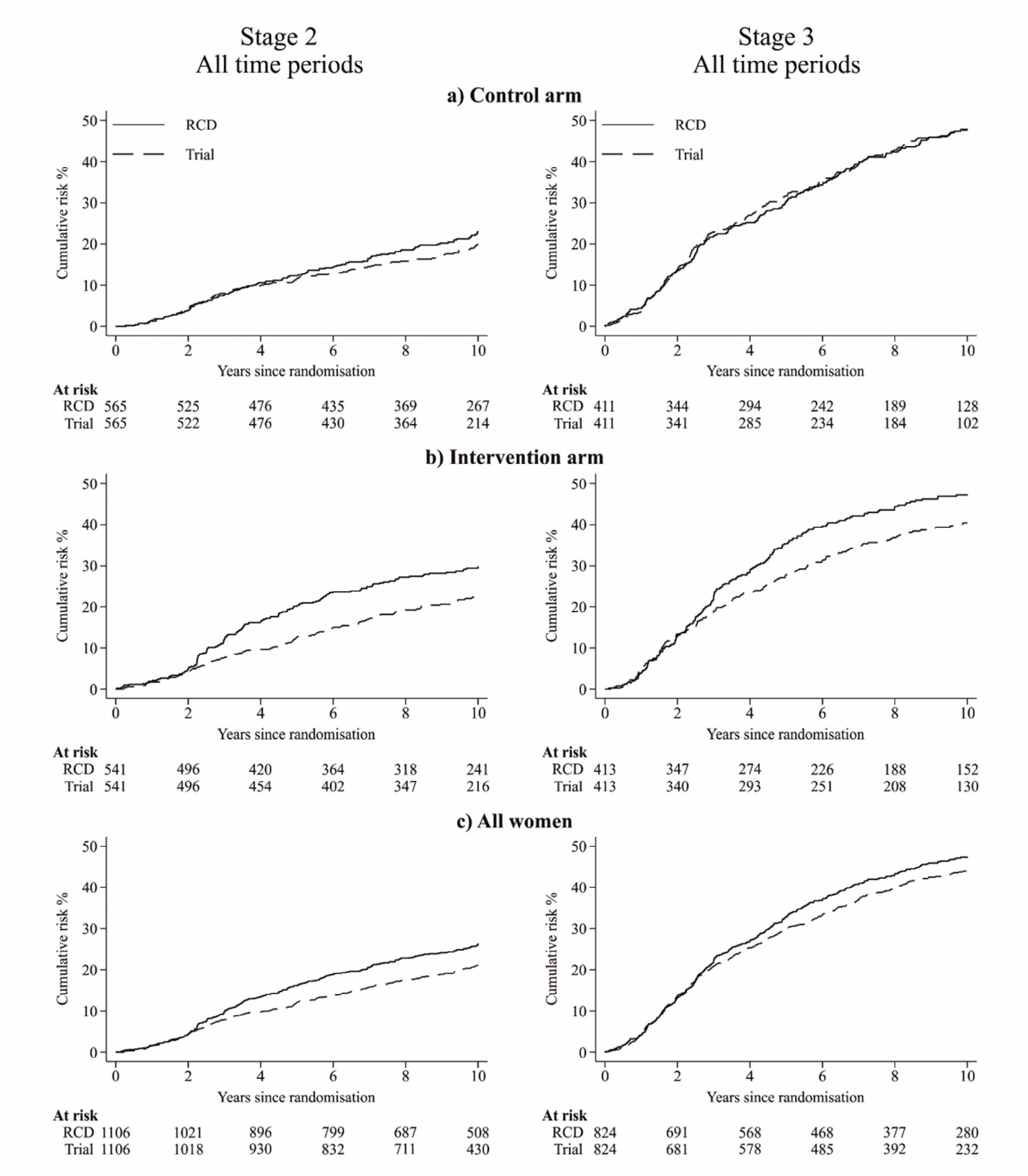

Figure S8. Internal Validation exercise: Cumulative risk of distant recurrence in AZURE trial (dashed lines) and in the routinely collected data (RCD) (solid lines), split by stage of disease. Analyses are by randomisation arm (a and b), and for all women included in the internal validation group (c).

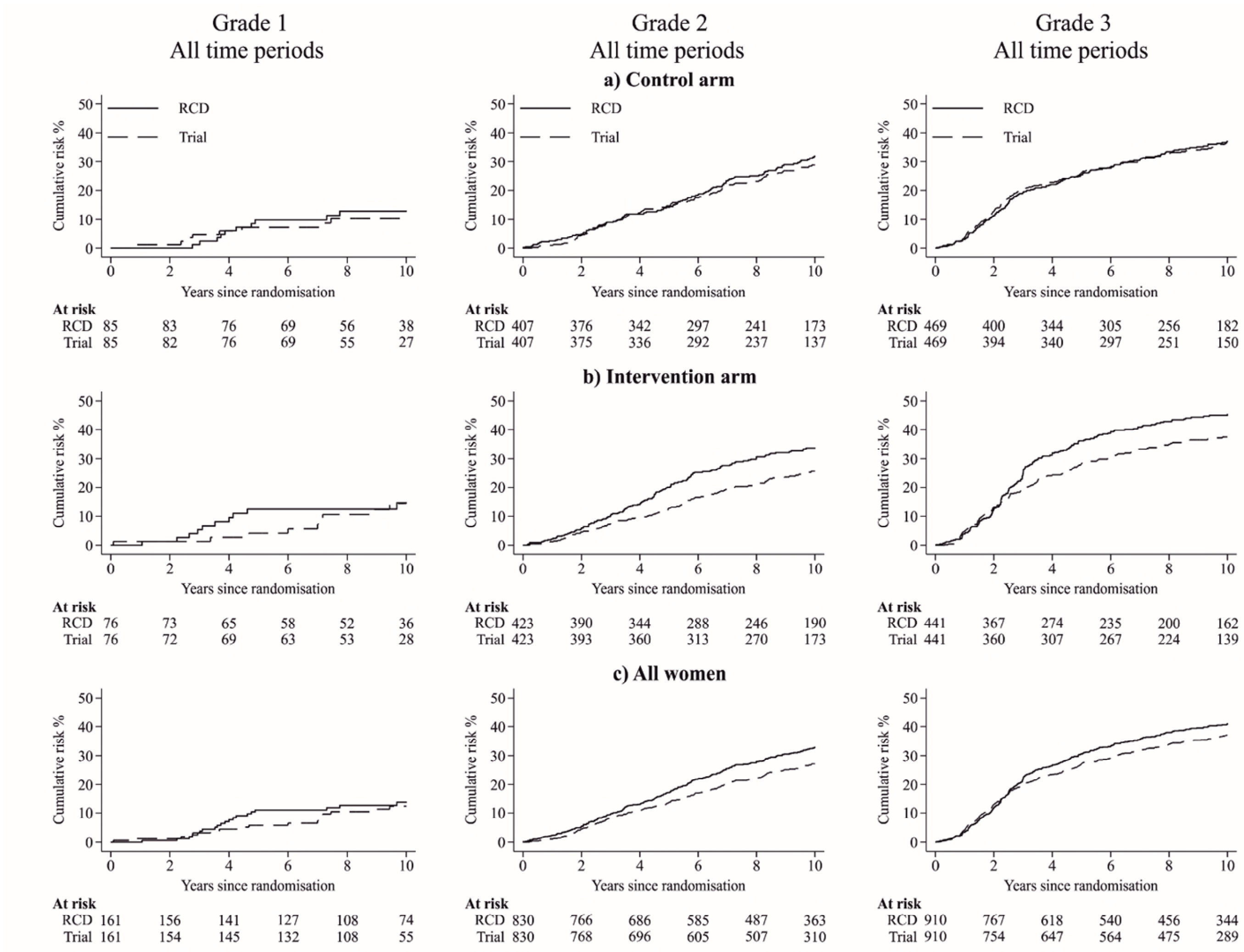

Figure S9. Internal Validation exercise: Cumulative risk of distant recurrence in AZURE trial (dashed lines) and in the routinely collected data (RCD) (solid lines), split by tumour grade. Analyses are by randomisation arm (a and b), and for all women included in the internal validation group (c). 29 women with unknown grade excluded from plots.

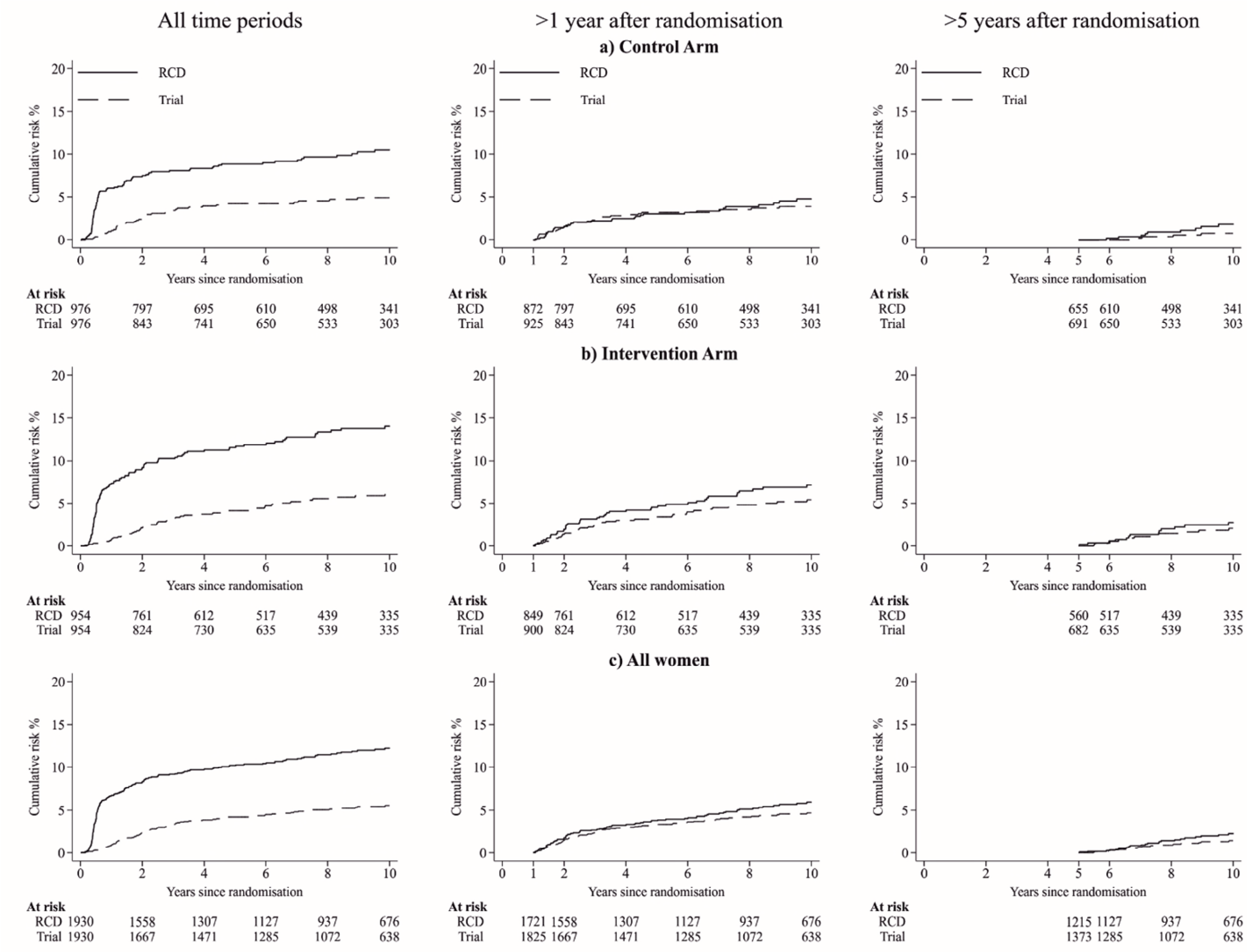

Figure S10. Internal validation exercise: Cumulative risk of locoregional recurrence in AZURE trial (dashed lines) and in routinely collected data (RCD) (solid lines). Analyses are by separate randomisation arm (a and b), and for all 1930 women included in the internal validation group (c). The outcome of locoregional recurrence is defined in Table S4. Plotted values at 1-, 3-, 5- and 10-years (and 95% confidence intervals) are in Table S18.

Table S18. Internal validation exercise: Number of events and cumulative risks of locoregional recurrence by time since randomisation and AZURE trial arm, for routinely collected data (RCD) and AZURE trial data. Calculated for all 1930 women in the internal validation group.

			All time periods			>1 year after randomisation			>5 years after randomisation		
**Trial Arm**	**Dataset**	**Time since randomisation (years)**	**No. women at risk[a]**	**No. events[b]**	**Cumulative risk[c] % (95% CI)**	**No. women at risk[a]**	**No. events[b]**	**Cumulative risk[c] % (95% CI)**	**No. women at risk[a]**	**No. events[b]**	**Cumulative risk[c] % (95% CI)**
**Control**	RCD	0-1	976	57	6.0 (4.6, 7.7)	-	-	-	-	-	-
		1-3	872	18	8.1 (6.5, 10.0)	872	18	2.2 (1.4, 3.5)	-	-	-
		3-5	736	6	8.9 (7.2, 10.9)	736	6	3.1 (2.1, 4.5)	-	-	-
		5-10	655	9	10.5 (8.6, 12.8)	655	9	4.8 (3.4, 6.7)	655	9	1.8 (0.9, 3.4)
	Trial	0-1	976	10	1.0 (0.6, 1.9)	-	-	-	-	-	-
		1-3	925	21	3.5 (2.4, 4.9)	925	21	2.4 (1.6, 3.7)	-	-	-
		3-5	782	6	4.2 (3.1, 5.8)	782	6	3.2 (2.2, 4.7)	-	-	-
		5-10	691	4	5.0 (3.7, 6.8)	691	4	4.0 (2.8, 5.7)	691	4	0.8 (0.3, 2.1)
**Intervention**	RCD	0-1	954	69	7.4 (5.9, 9.2)	-	-	-	-	-	-
		1-3	849	25	10.4 (8.5, 12.5)	849	25	3.2 (2.2, 4.7)	-	-	-
		3-5	673	9	11.7 (9.7, 14.0)	673	9	4.6 (3.3, 6.4)	-	-	-
		5-10	560	13	14.2 (11.9, 16.9)	560	13	7.4 (5.5, 9.8)	560	13	2.9 (1.7, 4.9)
	Trial	0-1	954	7	0.8 (0.4, 1.6)	-	-	-	-	-	-
		1-3	900	20	3.1 (2.1, 4.5)	900	20	2.4 (1.5, 3.6)	-	-	-
		3-5	772	8	4.2 (3.0, 5.8)	772	8	3.4 (2.4, 4.9)	-	-	-
		5-10	682	12	6.4 (4.8, 8.5)	682	12	5.7 (4.2, 7.7)	682	12	2.3 (1.3, 4.1)
**Total**	RCD	0-1	1930	126	6.7 (5.6, 7.9)	-	-	-	-	-	-
		1-3	1721	43	9.2 (8.0, 10.6)	1721	43	2.7 (2.0, 3.6)	-	-	-
		3-5	1409	15	10.2 (8.9, 11.7)	1409	15	3.8 (3.0, 4.9)	-	-	-
		5-10	1215	22	12.3 (10.8, 14.0)	1215	22	6.0 (4.8, 7.5)	1215	22	2.3 (1.5, 3.5)
	Trial	0-1	1930	17	0.9 (0.6, 1.4)	-	-	-	-	-	-
		1-3	1825	41	3.3 (2.5, 4.2)	1825	41	2.4 (1.8, 3.2)	-	-	-
		3-5	1554	14	4.2 (3.3, 5.3)	1554	14	3.3 (2.6, 4.3)	-	-	-
		5-10	1373	16	5.7 (4.6, 7.0)	1373	16	4.9 (3.8, 6.1)	1373	16	1.6 (1.0, 2.6)

[a] Number of women at risk at start of time category
[b] Number of events during time category
[c] Cumulative risk at end of time category

Table S19. Internal validation exercise: Agreement of routinely collected data (RCD) and AZURE trial data for locoregional recurrence. Calculations performed for all 1930 women in the internal validation group.

Locoregional recurrence[a]	All time periods			>1 year after randomisation[b]			>5 years after randomisation[b]		
	Control (N=976)	Intervention (N=954)	Total (N=1930)	Control (N=976)	Intervention (N=954)	Total (N=1930)	Control (N=976)	Intervention (N=954)	Total (N=1930)
					**No.**				
Event in both datasets	20	20	40	16	16	32	3	4	7
Event only in trial data	21	27	48	14	22	36	1	4	5
Event only in RCD	70	96	166	16	30	46	6	9	15
No event in either dataset	865	811	1676	812	762	1574	618	527	1145
**Censored before analysis period**									
Only in trial data	0	0	0	14	19	33	27	16	43
Only in RCD	0	0	0	67	70	137	63	138	201
In both datasets	0	0	0	37	35	72	258	256	514
**Time difference when event present in both datasets**					**No. (%)**				
<6 months	17 (85.0%)	19 (95.0%)	36 (90.0%)	14 (87.5%)	16 (100%)	30 (93.8%)	3 (100%)	4 (100%)	7 (100%)
6-12 months	2 (10.0%)	0 (0.0%)	2 (5.0%)	2 (12.5%)	0 (0.0%)	2 (6.2%)	0 (0.0%)	0 (0.0%)	0 (0.0%)
>1 year	1 (5.0%)	1 (5.0%)	2 (5.0%)	0 (0.0%)	0 (0.0%)	0 (0.0%)	0 (0.0%)	0 (0.0%)	0 (0.0%)
**Performance measures[c]**					**% (95% CI)**				
All time periods									
Sensitivity	48.8 (33.5, 64.1)	42.6 (28.4, 56.7)	45.5 (35.1, 55.9)	53.3 (35.5, 71.2)	42.1 (26.4, 57.8)	47.1 (35.2, 58.9)	75.0 (32.6, 100)	50.0 (15.4, 84.6)	58.3 (30.4, 86.2)
Specificity	92.5 (90.8, 94.2)	89.4 (87.4, 91.4)	91.0 (89.7, 92.3)	98.1 (97.1, 99.0)	96.2 (94.9, 97.5)	97.2 (96.4, 98.0)	99.0 (98.3, 99.8)	98.3 (97.2, 99.4)	98.7 (98.1, 99.4)
PPV	22.2 (13.6, 30.8)	17.2 (10.4, 24.1)	19.4 (14.0, 24.8)	50.0 (32.7, 67.3)	34.8 (21.0, 48.5)	41.0 (30.1, 51.9)	33.3 (2.5, 64.1)	30.8 (5.7, 55.9)	31.8 (12.4, 51.3)
NPV	97.6 (96.6, 98.6)	96.8 (95.6, 98.0)	97.2 (96.4, 98.0)	98.3 (97.4, 99.2)	97.2 (96.0, 98.3)	97.8 (97.0, 98.5)	99.8 (99.5, 100)	99.2 (98.5, 100)	99.6 (99.2, 99.9)
Within 6 months of trial data									
Sensitivity	41.5 (26.4, 56.5)	40.4 (26.4, 54.5)	40.9 (30.6, 51.2)	46.7 (28.8, 64.5)	42.1 (26.4, 57.8)	44.1 (32.3, 55.9)	75.0 (32.6, 100)	50.0 (15.4, 84.6)	58.3 (30.4, 86.2)
Specificity	92.5 (90.8, 94.2)	89.4 (87.4, 91.4)	91.0 (89.7, 92.3)	98.1 (97.1, 99.0)	96.2 (94.9, 97.5)	97.2 (96.4, 98.0)	99.0 (98.3, 99.8)	98.3 (97.2, 99.4)	98.7 (98.1, 99.4)
PPV	19.5 (11.2, 27.9)	16.5 (9.7, 23.3)	17.8 (12.5, 23.1)	46.7 (28.8, 64.5)	34.8 (21.0, 48.5)	39.5 (28.5, 50.5)	33.3 (2.5, 64.1)	30.8 (5.7, 55.9)	31.8 (12.4, 51.3)
NPV	97.3 (96.2, 98.4)	96.7 (95.4, 97.9)	97.0 (96.2, 97.8)	98.1 (97.1, 99.0)	97.2 (96.0, 98.3)	97.6 (96.9, 98.4)	99.8 (99.5, 100)	99.2 (98.5, 100)	99.6 (99.2, 99.9)

Analysis period and trial arm (spanning header over all columns)

PPV, Positive predictive value; NPV, Negative predictive value

[a] Women are censored at contralateral breast cancer, distant metastases, non-breast cancer malignancies and death (Table S4)

[b] Analysis period starts at 1 or 5 years after randomisation

[c] Performance measures defined in footnote of Table 3

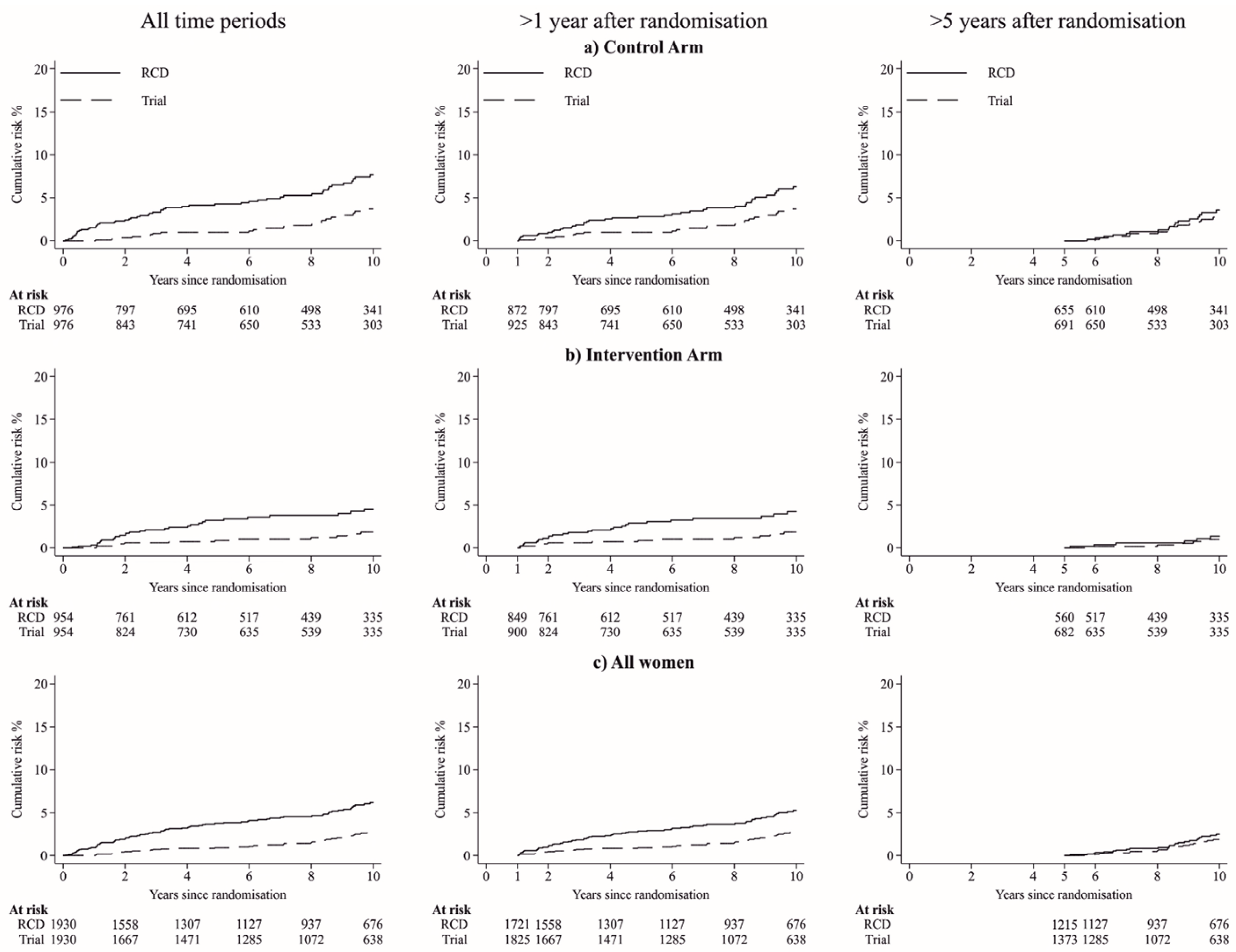

Figure S11. Internal validation exercise: Cumulative risk of contralateral breast cancer in AZURE trial (dashed lines) and in routinely collected data (RCD) (solid lines). Analyses are by separate randomisation arm (a and b), and for all 1930 women included in the internal validation group (c). The outcome of contralateral breast cancer is defined in Table S4. Plotted values at 1-, 3-, 5- and 10-years (and 95% confidence intervals) are in Table S20.

Table S20. Internal validation exercise: Number of events and cumulative risks of contralateral breast cancer by time since randomisation and AZURE trial arm, for routinely collected data (RCD) and AZURE trial data. Calculated for all 1930 women in the internal validation group.

			All time periods			>1 year after randomisation			>5 years after randomisation		
**Trial Arm**	**Dataset**	**Time since randomisation (years)**	**No. women at risk[a]**	**No. events[b]**	**Cumulative risk[c] % (95% CI)**	**No. women at risk[a]**	**No. events[b]**	**Cumulative risk[c] % (95% CI)**	**No. women at risk[a]**	**No. events[b]**	**Cumulative risk[c] % (95% CI)**
**Control**	RCD	0-1	976	14	1.5 (0.9, 2.5)	-	-	-	-	-	-
		1-3	872	15	3.3 (2.3, 4.8)	872	15	1.8 (1.1, 3.0)	-	-	-
		3-5	736	7	4.3 (3.1, 5.9)	736	7	2.8 (1.9, 4.3)	-	-	-
		5-10	655	17	7.5 (5.7, 9.8)	655	17	6.1 (4.5, 8.3)	655	17	3.4 (2.1, 5.3)
	Trial	0-1	976	0	0.0 (NA[d])	-	-	-	-	-	-
		1-3	925	7	0.8 (0.4, 1.7)	925	7	0.8 (0.4, 1.7)	-	-	-
		3-5	782	1	1.0 (0.5, 1.9)	782	1	1.0 (0.5, 1.9)	-	-	-
		5-10	691	14	3.7 (2.4, 5.6)	691	14	3.7 (2.4, 5.6)	691	14	2.8 (1.7, 4.6)
**Intervention**	RCD	0-1	954	3	0.3 (0.1, 1.0)	-	-	-	-	-	-
		1-3	849	14	2.1 (1.3, 3.4)	849	14	1.8 (1.1, 3.1)	-	-	-
		3-5	673	7	3.3 (2.2, 4.8)	673	7	2.9 (1.9, 4.5)	-	-	-
		5-10	560	6	4.5 (3.2, 6.5)	560	6	4.2 (2.9, 6.2)	560	6	1.3 (0.6, 2.9)
	Trial	0-1	954	0	0.0 (NA[d])	-	-	-	-	-	-
		1-3	900	5	0.6 (0.2, 1.4)	900	5	0.6 (0.2, 1.4)	-	-	-
		3-5	772	2	0.9 (0.4, 1.8)	772	2	0.9 (0.4, 1.8)	-	-	-
		5-10	682	5	1.8 (1.0, 3.3)	682	5	1.8 (1.0, 3.3)	682	5	1.0 (0.4, 2.3)
**Total**	RCD	0-1	1930	17	0.9 (0.6, 1.5)	-	-	-	-	-	-
		1-3	1721	29	2.7 (2.1, 3.7)	1721	29	1.8 (1.3, 2.6)	-	-	-
		3-5	1409	14	3.8 (2.9, 4.8)	1409	14	2.9 (2.1, 3.9)	-	-	-
		5-10	1215	23	6.1 (4.9, 7.5)	1215	23	5.2 (4.1, 6.6)	1215	23	2.4 (1.6, 3.6)
	Trial	0-1	1930	0	0.0 (NA[d])	-	-	-	-	-	-
		1-3	1825	12	0.7 (0.4, 1.2)	1825	12	0.7 (0.4, 1.2)	-	-	-
		3-5	1554	3	0.9 (0.5, 1.5)	1554	3	0.9 (0.5, 1.5)	-	-	-
		5-10	1373	19	2.8 (2.0, 3.9)	1373	19	2.8 (2.0, 3.9)	1373	19	1.9 (1.2, 2.9)

[a] Number of women at risk at start of time category [b] Number of events during time category [c] Cumulative risk at end of time category
[d] Unable to calculate confidence interval when no events are observed

Table S21. Internal validation exercise: Agreement of routinely collected data (RCD) and AZURE trial data for contralateral breast cancer. Calculations performed for all 1930 women in the internal validation group.

Contralateral breast cancer[a]	Analysis period and trial arm								
	All time periods			>1 year after randomisation[b]			>5 years after randomisation[b]		
	Control (N=976)	Intervention (N=954)	Total (N=1930)	Control (N=976)	Intervention (N=954)	Total (N=1930)	Control (N=976)	Intervention (N=954)	Total (N=1930)
					**No.**				
Event in both datasets	18	8	26	18	8	26	13	2	15
Event only in trial data	4	4	8	2	3	5	1	1	2
Event only in RCD	35	22	57	20	18	38	4	3	7
No event in either dataset	919	920	1839	818	801	1619	610	538	1148
**Censored before analysis period**									
Only in trial data	0	0	0	14	19	33	27	16	43
Only in RCD	0	0	0	67	70	137	63	138	201
In both datasets	0	0	0	37	35	72	258	256	514
**Time difference when event present in both datasets**					**No. (%)**				
<6 months	18 (100%)	8 (100%)	26 (100%)	18 (100%)	8 (100%)	26 (100%)	13 (100%)	2 (100%)	15 (100%)
6-12 months	0 (0.0%)	0 (0.0%)	0 (0.0%)	0 (0.0%)	0 (0.0%)	0 (0.0%)	0 (0.0%)	0 (0.0%)	0 (0.0%)
>1 year	0 (0.0%)	0 (0.0%)	0 (0.0%)	0 (0.0%)	0 (0.0%)	0 (0.0%)	0 (0.0%)	0 (0.0%)	0 (0.0%)
**Performance measures[c]**					**% (95% CI)**				
All time periods									
Sensitivity	81.8 (65.7, 97.9)	66.7 (40.0, 93.3)	76.5 (62.2, 90.7)	90.0 (76.9, 100)	72.7 (46.4, 99.0)	83.9 (70.9, 96.8)	92.9 (79.4, 100)	66.7 (13.3, 100)	88.2 (72.9, 100)
Specificity	96.3 (95.1, 97.5)	97.7 (96.7, 98.6)	97.0 (96.2, 97.8)	97.6 (96.6, 98.6)	97.8 (96.8, 98.8)	97.7 (97.0, 98.4)	99.3 (98.7, 100)	99.4 (98.8, 100)	99.4 (98.9, 99.8)
PPV	34.0 (21.2, 46.7)	26.7 (10.8, 42.5)	31.3 (21.3, 41.3)	47.4 (31.5, 63.2)	30.8 (13.0, 48.5)	40.6 (28.6, 52.7)	76.5 (56.3, 96.6)	40.0 (0.0, 82.9)	68.2 (48.7, 87.6)
NPV	99.6 (99.1, 100)	99.6 (99.1, 100)	99.6 (99.3, 99.9)	99.8 (99.4, 100)	99.6 (99.2, 100)	99.7 (99.4, 100)	99.8 (99.5, 100)	99.8 (99.5, 100)	99.8 (99.6, 100)
Within 6 months of trial data									
Sensitivity	81.8 (65.7, 97.9)	66.7 (40.0, 93.3)	76.5 (62.2, 90.7)	90.0 (76.9, 100)	72.7 (46.4, 99.0)	83.9 (70.9, 96.8)	92.9 (79.4, 100)	66.7 (13.3, 100)	88.2 (72.9, 100)
Specificity	96.3 (95.1, 97.5)	97.7 (96.7, 98.6)	97.0 (96.2, 97.8)	97.6 (96.6, 98.6)	97.8 (96.8, 98.8)	97.7 (97.0, 98.4)	99.3 (98.7, 100)	99.4 (98.8, 100)	99.4 (98.9, 99.8)
PPV	34.0 (21.2, 46.7)	26.7 (10.8, 42.5)	31.3 (21.3, 41.3)	47.4 (31.5, 63.2)	30.8 (13.0, 48.5)	40.6 (28.6, 52.7)	76.5 (56.3, 96.6)	40.0 (0.0, 82.9)	68.2 (48.7, 87.6)
NPV	99.6 (99.1, 100)	99.6 (99.1, 100)	99.6 (99.3, 99.9)	99.8 (99.4, 100)	99.6 (99.2, 100)	99.7 (99.4, 100)	99.8 (99.5, 100)	99.8 (99.5, 100)	99.8 (99.6, 100)

PPV, Positive predictive value; NPV, Negative predictive value

[a] Women are censored at locoregional recurrence, distant metastases, non-breast cancer malignancies and death (Table S4)

[b] Analysis period starts at 1 or 5 years after randomisation

[c] Performance measures defined in footnote of Table 3

Table S22. Internal validation exercise: Number of events and cumulative risks of any recurrence by time since randomisation and AZURE trial arm, for routinely collected data (RCD) and AZURE trial data. Calculated for all 1930 women in the internal validation group.

Trial Arm and Dataset	Time since random-isation (years)	All time periods			>1 year after randomisation			>5 years after randomisation			All time periods with post-validation adjustment		
		No. women at risk[a]	No. events[b]	Cumulative risk[c] % (95% CI)	No. women at risk[a]	No. events[b]	Cumulative risk[c] % (95% CI)	No. women at risk[a]	No. events[b]	Cumulative risk[c] % (95% CI)	No. women at risk[a]	No. events[b]	Cumulative risk[c] % (95% CI)
**Control**													
RCD	0-1	976	107	11.0 (9.2, 13.2)	-	-	-	-	-	-	976	27	2.8 (1.9, 4.0)
	1-3	857	128	24.5 (21.9, 27.3)	857	128	15.1 (12.9, 17.7)	-	-	-	936	132	16.7 (14.4, 19.2)
	3-5	709	56	30.5 (27.7, 33.6)	709	56	21.9 (19.3, 24.9)	-	-	-	783	65	23.7 (21.1, 26.5)
	5-10	631	100	43.6 (40.4, 47.0)	631	100	36.6 (33.3, 40.2)	631	100	18.8 (15.7, 22.4)	693	115	38.6 (35.4, 42.0)
Trial	0-1	976	32	3.3 (2.4, 4.6)	-	-	-	-	-	-	976	32	3.3 (2.4, 4.6)
	1-3	925	124	16.4 (14.2, 18.9)	925	124	13.5 (11.5, 15.9)	-	-	-	925	124	16.4 (14.2, 18.9)
	3-5	782	52	22.1 (19.6, 24.9)	782	52	19.4 (17.0, 22.2)	-	-	-	782	52	22.1 (19.6, 24.9)
	5-10	691	94	35.6 (32.4, 39.0)	691	94	33.4 (30.2, 36.8)	691	94	17.3 (14.4, 20.7)	691	94	35.6 (32.4, 39.0)
**Intervention**													
RCD	0-1	954	108	11.4 (9.5, 13.6)	-	-	-	-	-	-	954	28	3.0 (2.1, 4.3)
	1-3	835	181	30.8 (28.0, 33.9)	835	181	22.0 (19.3, 24.9)	-	-	-	909	166	20.9 (18.4, 23.7)
	3-5	633	97	41.6 (38.5, 44.9)	633	97	34.1 (31.0, 37.5)	-	-	-	719	117	34.0 (31.0, 37.2)
	5-10	512	87	52.9 (49.6, 56.2)	512	87	46.8 (43.3, 50.4)	512	87	19.2 (15.9, 23.2)	576	96	46.6 (43.3, 50.0)
Trial	0-1	954	33	3.5 (2.5, 4.9)	-	-	-	-	-	-	954	33	3.5 (2.5, 4.9)
	1-3	900	104	14.8 (12.7, 17.3)	900	104	11.7 (9.8, 14.0)	-	-	-	900	104	14.8 (12.7, 17.3)
	3-5	772	59	21.4 (18.9, 24.3)	772	59	18.6 (16.2, 21.3)	-	-	-	772	59	21.4 (18.9, 24.3)
	5-10	682	88	34.0 (30.8, 37.4)	682	88	31.6 (28.4, 35.0)	682	88	15.9 (13.1, 19.3)	682	88	34.0 (30.8, 37.4)
**Total**													
RCD	0-1	1930	215	11.2 (9.9, 12.7)	-	-	-	-	-	-	1930	55	2.9 (2.2, 3.7)
	1-3	1692	309	27.6 (25.7, 29.7)	1692	309	18.5 (16.7, 20.4)	-	-	-	1845	298	18.8 (17.1, 20.6)
	3-5	1342	153	36.0 (33.9, 38.2)	1342	153	27.9 (25.8, 30.2)	-	-	-	1502	182	28.8 (26.8, 30.9)
	5-10	1143	187	48.2 (45.8, 50.6)	1143	187	41.6 (39.2, 44.2)	1143	187	19.0 (16.7, 21.6)	1269	211	42.5 (40.2, 44.9)
Trial	0-1	1930	65	3.4 (2.7, 4.3)	-	-	-	-	-	-	1930	65	3.4 (2.7, 4.3)
	1-3	1825	228	15.6 (14.0, 17.3)	1825	228	12.6 (11.2, 14.3)	-	-	-	1825	228	15.6 (14.0, 17.3)
	3-5	1554	111	21.8 (20.0, 23.7)	1554	111	19.0 (17.3, 20.9)	-	-	-	1554	111	21.8 (20.0, 23.7)
	5-10	1373	182	34.8 (32.5, 37.1)	1373	182	32.5 (30.2, 34.9)	1373	182	16.6 (14.5, 18.9)	1373	182	34.8 (32.5, 37.1)

[a] Number of women at risk at start of time category [b] Number of events during time category [c] Cumulative risk at end of time category

Table S23. Internal validation exercise: Number of events and cumulative risks of breast cancer mortality and all-cause mortality by time since randomisation and AZURE trial arm, for routinely collected data (RCD) and AZURE trial data. Calculated for all 1930 women in the internal validation group.

Trial Arm	Dataset	Time since randomisation (years)	Breast cancer mortality: No. women at risk[a]	Breast cancer mortality: No. events[b]	Breast cancer mortality: Cumulative risk[c] % (95% CI)	All-cause mortality: No. women at risk[a]	All-cause mortality: No. events[b]	All-cause mortality: Cumulative risk[c] % (95% CI)
**Control**	RCD	0-1	976	9	0.9 (0.5, 1.8)	976	14	1.4 (0.9, 2.4)
		1-3	954	59	7.1 (5.6, 8.9)	954	64	8.1 (6.5, 10.0)
		3-5	878	64	14.0 (11.9, 16.4)	878	70	15.5 (13.4, 18.0)
		5-10	786	82	24.6 (21.8, 27.6)	786	105	28.6 (25.7, 31.7)
	Trial	0-1	976	8	0.8 (0.4, 1.6)	976	14	1.4 (0.9, 2.4)
		1-3	951	61	7.2 (5.8, 9.1)	951	65	8.2 (6.6, 10.1)
		3-5	876	61	13.8 (11.8, 16.2)	876	68	15.5 (13.3, 17.9)
		5-10	778	90	26.5 (23.6, 29.7)	778	105	29.8 (26.8, 33.0)
**Intervention**	RCD	0-1	954	9	1.0 (0.5, 1.8)	954	13	1.4 (0.8, 2.3)
		1-3	930	58	7.2 (5.7, 9.1)	930	63	8.1 (6.5, 10.1)
		3-5	846	49	12.7 (10.7, 15.0)	846	58	14.5 (12.4, 16.9)
		5-10	768	95	24.9 (22.1, 28.0)	768	111	28.3 (25.4, 31.5)
	Trial	0-1	954	7	0.7 (0.4, 1.5)	954	12	1.3 (0.7, 2.2)
		1-3	929	60	7.2 (5.7, 9.1)	929	64	8.1 (6.6, 10.1)
		3-5	844	51	12.9 (10.9, 15.3)	844	58	14.5 (12.4, 17.0)
		5-10	767	98	26.6 (23.7, 29.9)	767	112	29.7 (26.7, 33.0)
**Total**	RCD	0-1	1930	18	0.9 (0.6, 1.5)	1930	27	1.4 (1.0, 2.0)
		1-3	1884	117	7.2 (6.1, 8.4)	1884	127	8.1 (7.0, 9.4)
		3-5	1724	113	13.3 (11.9, 15.0)	1724	128	15.0 (13.5, 16.7)
		5-10	1554	177	24.7 (22.8, 26.9)	1554	216	28.5 (26.4, 30.7)
	Trial	0-1	1930	15	0.8 (0.5, 1.3)	1930	26	1.4 (0.9, 2.0)
		1-3	1880	121	7.2 (6.2, 8.5)	1880	129	8.2 (7.0, 9.5)
		3-5	1720	112	13.4 (11.9, 15.0)	1720	126	15.0 (13.5, 16.7)
		5-10	1545	188	26.6 (24.5, 28.8)	1545	217	29.8 (27.6, 32.0)

[a] Number of women at risk at the start of the time category
[b] Number of events during time category
[c] Cumulative risk at end of time category

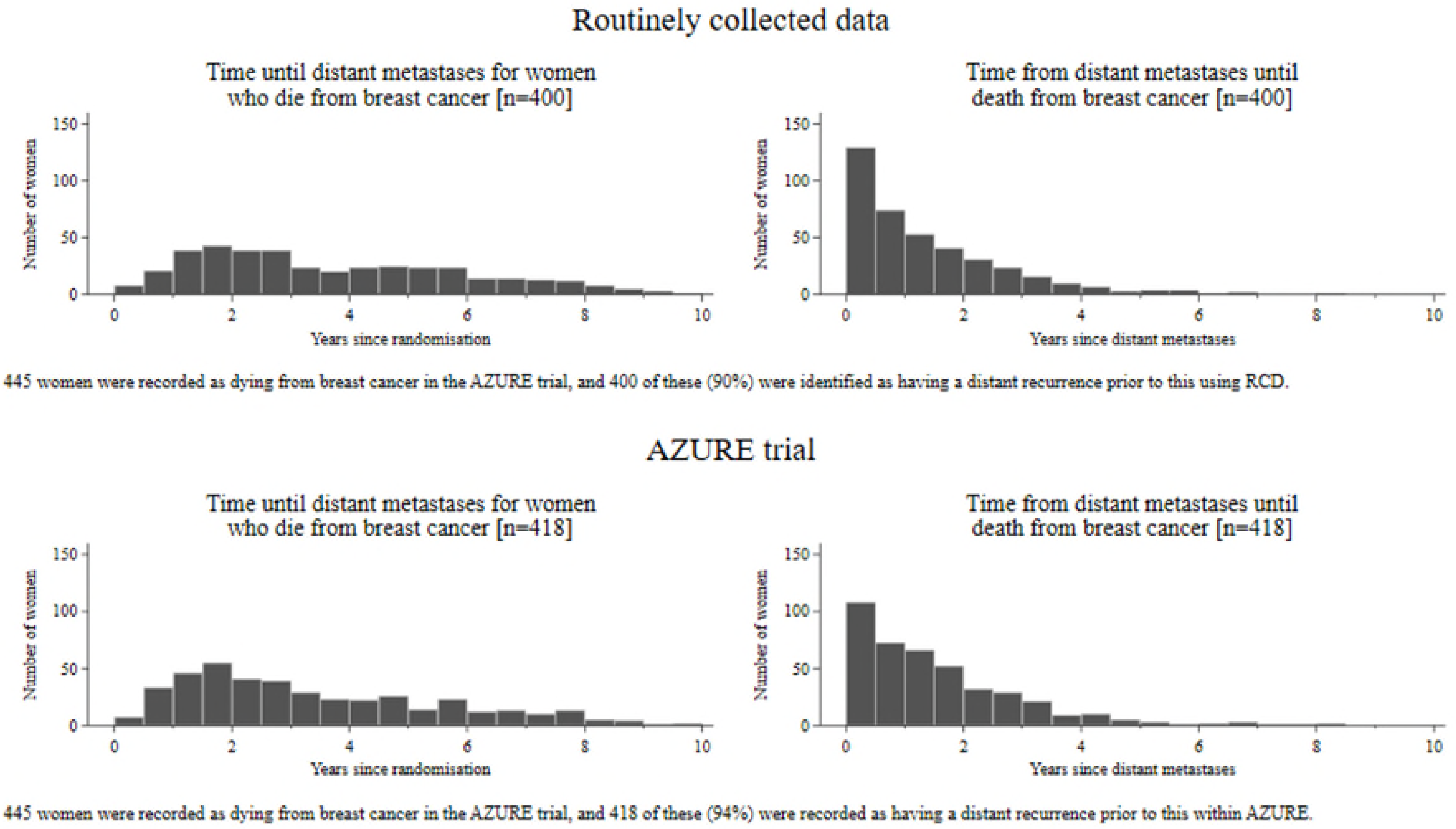

Figure S12. Internal validation exercise: Distribution of time until distant metastasis and then to death from breast cancer for 445 women recorded as dying from breast cancer in trial data.

Table S24. Internal validation exercise: Agreement of routinely collected data (RCD) and AZURE trial data for breast cancer mortality and all-cause mortality, by AZURE trial arm. Calculations performed for all 1930 women in the internal validation group.

	**Outcome and trial arm**					
	**Breast cancer mortality[a]**			**All-cause mortality**		
	**Control (N=976)**	**Intervention (N=954)**	**Total (N=1930)**	**Control (N=976)**	**Intervention (N=954)**	**Total (N=1930)**
			**No.**			
Event in both datasets	210	210	420	260	250	510
Event only in trial data	17	8	25[b]	0	1	1[c]
Event only in RCD	10	4	14[d]	1	1	2[e]
No event in either dataset	739	732	1471	715	702	1417
**Time difference when event present in both datasets**			**No. (%)**			
<6 months	210 (100%)	209 (99.5%)	419 (99.8%)	260 (100%)	249 (99.6%)	509 (99.8%)
6-12 months	0 (0.0%)	0 (0.0%)	0 (0.0%)	0 (0.0%)	0 (0.0%)	0 (0.0%)
>1 year	0 (0.0%)	1 (0.5%)	1 (0.2%)	0 (0.0%)	1 (0.4%)	1 (0.2%)
**Performance measures[f]**			**% (95% CI)**			
All time periods						
Sensitivity	92.5 (89.1, 95.9)	96.3 (93.8, 98.8)	94.4 (92.2, 96.5)	100 (100, 100)	99.6 (98.8, 100)	99.8 (99.4, 100)
Specificity	98.7 (97.8, 99.5)	99.5 (98.9, 100)	99.1 (98.6, 99.5)	99.9 (99.6, 100)	99.9 (99.6, 100)	99.9 (99.7, 100)
PPV	95.5 (92.7, 98.2)	98.1 (96.3, 99.9)	96.8 (95.1, 98.4)	99.6 (98.9, 100)	99.6 (98.8, 100)	99.6 (99.1, 100)
NPV	97.8 (96.7, 98.8)	98.9 (98.2, 99.7)	98.3 (97.7, 99.0)	100 (100, 100)	99.9 (99.6, 100)	99.9 (99.8, 100)
Within 6 months of trial data						
Sensitivity	92.5 (89.1, 95.9)	95.9 (93.2, 98.5)	94.2 (92.0, 96.3)	100 (100, 100)	99.2 (98.1, 100)	99.6 (99.1, 100)
Specificity	98.7 (97.8, 99.5)	99.5 (98.9, 100)	99.1 (98.6, 99.5)	99.9 (99.6, 100)	99.9 (99.6, 100)	99.9 (99.7, 100)
PPV	95.5 (92.7, 98.2)	98.1 (96.3, 99.9)	96.8 (95.1, 98.4)	99.6 (98.9, 100)	99.6 (98.8, 100)	99.6 (99.1, 100)
NPV	97.8 (96.7, 98.8)	98.8 (98.0, 99.6)	98.3 (97.6, 98.9)	100 (100, 100)	99.7 (99.3, 100)	99.9 (99.7, 100)

PPV, Positive predictive value; NPV, Negative predictive value

[a] Women are censored at non-breast-cancer deaths (Table S4)

[b] All had a non-breast-cancer death identified in RCD within 3 weeks of trial death: 14 were classified as death from non-breast cancer; 7 as death from cause other than cancer; and 4 of unknown cause.

[c] Trial data recorded death from cause other than cancer and the same event was identified in RCD >3 months after trial (so censored before death).

[d] 13 had a non-breast-cancer death recorded in trial data within 3 weeks of RCD breast death: 1 was a death from non-breast cancer; 6 as death from cause other than cancer; and 6 of unknown cause. 1 had no death recorded in trial data (see footnote ZZ).

[e] 1 had breast death identified in RCD after date of withdrawal from trial, and the other had death from non-breast cancer >10 years after randomisation (study endpoint). Both deaths were within the 3 month window for RCD.

[f] Performance measures defined in footnote of Table 3

Table S25. Previous work in England comparing outcomes in routine data held by the former National Cancer Data Repository with those reported in the TACT (Taxotere and Adjuvant ChemoTherapy) trial. Based on Kilburn et al.[1]

	**Local recurrence (N = 140)**	**Distant recurrence (N=691)**	**New breast disease[a] (N = 67)**	**Any breast disease[b] (N=898)**
Agreement	98	425	54	577
Event reported in TACT but not in NCDR	17	76	2	95
Disagreement in number of sites and/or diagnosis time	23	168	10	201
Event occurred after 31 March 2010 (HES extract date)	2	22	1	25
Sensitivity excluding events where there was disagreement in number of sites and/or diagnosis time[c]	71.0%	63.5%	81.8%	66.1%
Sensitivity including events where there was disagreement in number of sites and/or diagnosis time[c]	87.9%	88.6%	97.0%	89.1%

TACT Taxotere and Adjuvant ChemoTherapy, *NCDR* National Cancer Data Repository, *HES* Hospital Episode Statistics

[a] i.e. New breast second primary cancer, including contralateral breast cancer

[b] i.e. Local recurrence, distant recurrence, or new breast disease.

[c] Denominators exclude events occurring after 31 March 2010 (HES extract date).

Reference

1. Kilburn LS, Aresu M, Banerji J, Barrett-Lee P, Ellis P, Bliss JM. Can routine data be used to support cancer clinical trials? A historical baseline on which to build: retrospective linkage of data from the TACT (CRUK 01/001) breast cancer trial and the National Cancer Data Repository. *Trials* 2017; **18**(1): 561.
